# ShennongAlpha: an AI-driven sharing and collaboration platform for intelligent curation, acquisition, and translation of natural medicinal material knowledge

**DOI:** 10.1038/s41421-025-00776-2

**Published:** 2025-04-01

**Authors:** Zijie Yang, Yongjing Yin, Chaojun Kong, Tiange Chi, Wufan Tao, Yue Zhang, Tian Xu

**Affiliations:** 1 Fudan University, Shanghai, China; 2 Key Laboratory of Growth Regulation and Translational Research of Zhejiang Province, School of Life Sciences, Westlake University, Hangzhou, Zhejiang China; 3 Westlake Laboratory of Life Sciences and Biomedicine, Hangzhou, Zhejiang China; 4 Research Center for Industries of the Future, Westlake University, Hangzhou, Zhejiang China; 5 Shennong Program, Westlake University, Hangzhou, Zhejiang China; 6 School of Engineering, Westlake University, Hangzhou, Zhejiang China; 7 Changping Laboratory, Beijing, China

**Keywords:** Biological techniques, Bioinformatics

## Abstract

Natural Medicinal Materials (NMMs) have a long history of global clinical applications and a wealth of records and knowledge. Although NMMs are a major source for drug discovery and clinical application, the utilization and sharing of NMM knowledge face crucial challenges, including the standardized description of critical information, efficient curation and acquisition, and language barriers. To address these, we developed ShennongAlpha, an artificial intelligence (AI)-driven sharing and collaboration platform for intelligent knowledge curation, acquisition, and translation. For standardized knowledge curation, the platform introduced a Systematic Nomenclature to enable accurate differentiation and identification of NMMs. More than fourteen thousand Chinese NMMs have been curated into the platform along with their knowledge. Furthermore, the platform pioneered chat-based knowledge acquisition, standardized machine translation, and collaborative knowledge updating. Together, our study represents the first major advance in leveraging AI to empower NMM knowledge sharing, which not only marks a novel application of AI for science, but also will significantly benefit the global biomedical, pharmaceutical, physician, and patient communities.

## Introduction

Natural Medicinal Materials (NMMs) have been a rich reservoir of therapeutic agents^1^. Their importance is highlighted by the diversity and biological relevance of the compounds they produce^2,3^. The compounds isolated from NMMs or their derivatives have been instrumental in the treatment of various pathological conditions, ranging from infectious diseases to cancer, and continue to serve as a prolific source of novel drug leads^4,5^. In fact, around 50% of the FDA-approved drugs are natural products or related molecules^1^. Furthermore, NMMs have been directly used for therapeutic purposes throughout human history and continue to play a significant role in different societies. The broad clinical application of NMMs has been documented in various countries and regions, including China^6^, Japan^7^, South Korea^8^, India^9^, Iran^10^, Europe^11,12^, the Americas^13,14^, Africa^15,16^, and the Arab region^17,18^. For example, Chinese historical records alone documented nearly ten thousand NMMs and their applications^19^. This vast repository of knowledge has proven to be valuable in discovering new therapies^20–22^. Therefore, the global utilization, sharing, and collaboration of NMM knowledge are crucial for advancing biomedical and pharmaceutical research and applications.

**Fig. 1:**
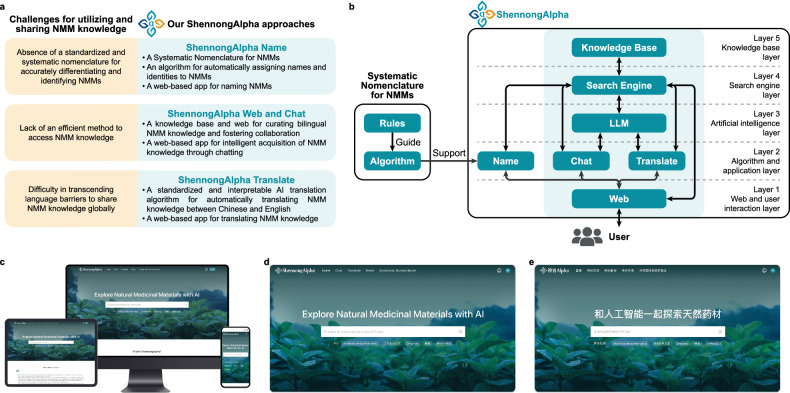
ShennongAlpha: an artificial intelligence (AI)-driven sharing and collaboration platform for intelligent curation, acquisition, and translation of NMM knowledge. **a** The challenges for utilizing and sharing NMM knowledge and our ShennongAlpha approaches. **b** Architecture of ShennongAlpha. ShennongAlpha applies the Systematic Nomenclature for NMMs (Fig. 2) and integrates ShennongName (Fig. 3) with a hexa-domain modular system to form its structure. The hexa-domain system is outlined in the light teal block. ShennongAlpha is structured into five layers, from shallow to deep. Layer 1: web and user interaction layer. In this layer, users can access the system via our ShennongAlpha Web (Fig. 4). Layer 2: algorithm and application layer. In this layer, we have specifically developed three applications customized for NMMs: ShennongName, ShennongChat (Fig. 5), and ShennongTranslate (Fig. 6). Users can access these applications on the corresponding pages of the ShennongAlpha Web. Layer 3: AI layer. In this layer, we have integrated the ShennongAlpha LLM system, allowing the ShennongAlpha to process and respond to data from different layers intelligently. Layer 4: search engine layer. In this layer, we have integrated the ShennongAlpha Search Engine customized for NMM-related data. Layer 5: knowledge base layer. In this layer, we have integrated the ShennongAlpha Knowledge Base to curate NMM knowledge. Arrows represent the allowed data interactions between different layers. **c** Cross-platform and user-friendly design of the ShennongAlpha. **d** The English homepage of the ShennongAlpha Web. **e** The Chinese homepage of the ShennongAlpha Web.

However, the great potential of NMMs has not been fully explored despite their diverse chemical reservoirs, rich history of human usage, and vast amounts of accumulated records and knowledge. The biomedical, pharmaceutical, physician, and patient communities face several challenges in utilizing and sharing NMM knowledge (Fig. 1a). At the center of the challenges is the conspicuous absence of a standardized and Systematic Nomenclature capable of accurately differentiating and identifying each NMM. This magnifies the difficulty of efficiently procuring accurate and reliable knowledge about NMMs. The enriched information and records for NMMs further pose challenges for curation and acquisition. Finally, language barriers also hinder the global dissemination and utilization of NMM knowledge.

**Fig. 2:**
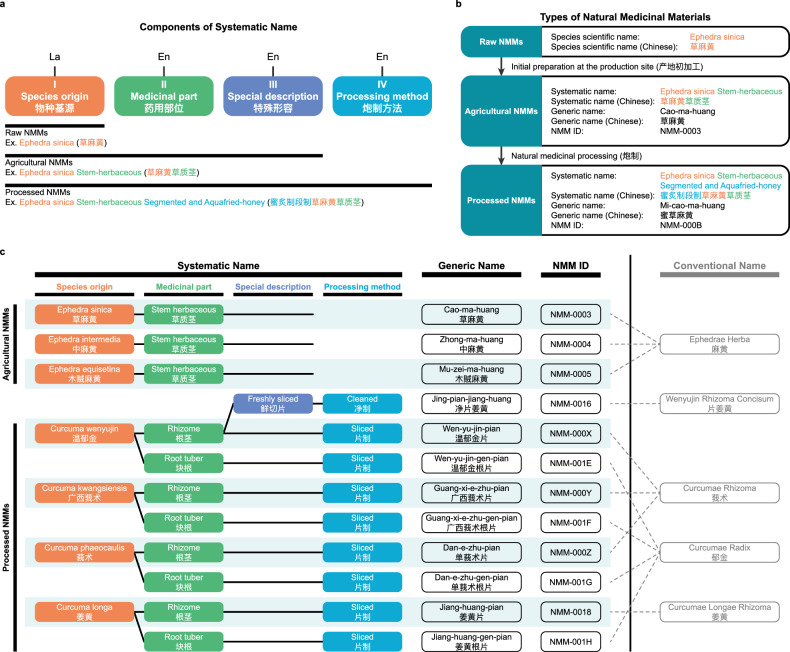
Systematic Nomenclature for NMMs. The Systematic Nomenclature assigns each NMM a unique Systematic Name, Generic Name, and NMM ID. **a** Components of the Systematic Name. It consists of four components: I. Species origin, including species names in Latin; II. Medicinal part; III. Special description for initial preparations or specific characteristics; and IV. Processing method. **b** NMM types. Raw NMMs are initially prepared at the production sites to produce Agricultural NMMs. Agricultural NMMs are often further processed to produce Processed NMMs. **c** Examples of traditional Chinese NMMs in Systematic Nomenclature. Conventional names often lead to confusion by collectively referring to multiple NMMs that are not identical, due to missing or incorrect information about species origin, medicinal part, special description, and processing method. For example, the illustration shows three Agricultural NMMs from the *Ephedra* genus with the herbaceous stem as the medicinal part, conventionally named “Ephedrae Herba” (“麻黄”), leading to ambiguity. Similarly, nine Processed NMMs from the *Curcuma* genus, with different medicinal parts, initial preparations and processing methods, are collectively referred to by four names: “Wenyujin Rhizoma Concisum” (“片姜黄”), “Curcumae Rhizoma” (“莪术”), “Curcumae Radix” (“郁金”), and “Curcumae Longae Rhizoma” (“姜黄”). In contrast, our Systematic Nomenclature accurately assigns distinct Systematic Names, Generic Names, and NMM IDs to these twelve different Agricultural and Processed NMMs, eliminating ambiguity. The dashed lines connect the conventional names to the different NMMs they collectively represent.

The problem of lacking standardized and Systematic Nomenclature for NMMs can be exemplified by the discovery of the anti-malaria drug artemisinin, which was awarded the 2015 Nobel Prize in Physiology or Medicine. This discovery emerged from historically documented malaria-treating NMM with the Chinese name “Qing Hao”. However, this name could refer to at least six different plants in the genus *Artemisia*. The artemisinin molecule was eventually found only in *Artemisia annua* with the Chinese name of “Huang Hua Hao”, but not in five other plants, including the modern-day botanic plant with the Chinese name of “Qing Hao” (*Artemisia caruifolia*)^21^. Although the *Chinese Pharmacopoeia (2020 edition)* now specifies *Artemisia annua* as the sole NMM suitable for medicinal use^23^, over three-quarters of the NMMs in the current edition still have ambiguous names (Supplementary Table S1). For example, the NMM “Ma Huang” (also known as “Ephedra” or “Ephedrae Herba”) corresponds to three species: *Ephedra sinica*, *Ephedra intermedia*, or *Ephedra equisetina* (Fig. 2c). The same problem exists for NMMs in other countries. In the *Indian Pharmacopoeia*, for instance, the name “Acacia” pertains to multiple species within the *Acacia* genus^24^. Similar ambiguity problems also exist regarding the descriptions of the parts used for medicinal purposes and the preparation processes of NMMs (Supplementary Table S1).

The absence of a standardized and Systematic Nomenclature for defining NMMs further complicates both the curation of related information and the acquisition of reliable and consistent knowledge about these NMMs. For example, Wikipedia often provides misleading entries for NMMs. The entry for “Ephedra” or “Ma Huang” misleadingly describes “Ephedra as a medicinal preparation from the plant *Ephedra sinica*” (Supplementary Fig. S1). Chat-based platforms like ChatGPT also exacerbate these inaccuracies, as demonstrated by their incorrect assertion that *Ephedra sinica* is the sole species origin for Ephedra (Supplementary Fig. S2). The root cause of these issues lies in the current absence of a standardized and systematic approach to accurately differentiate and identify each unique NMM with the information regarding its species origin, medicinal part, special description, and processing method, and to correctly catalog its related knowledge. Given the vast number of NMMs, along with their extensive and yet dispersed records, it is, therefore, a daunting task to curate the related information using traditional approaches and to facilitate efficient knowledge acquisition.

The lack of a multilingual platform for NMMs presents a significant barrier for the global biomedical and pharmaceutical communities, as well as physicians and patients. The absence of a standardized and Systematic Nomenclature also impedes the global sharing and dissemination of knowledge about NMMs. This issue is evident when translating the Chinese sentence regarding the NMM entry “Ma Huang” (麻黄是一种天然药材) via Google Translate. The resulting translation of “Ephedra is a natural medicine” implies a single plant rather than the multi-species intricacies of “Ma Huang” (Supplementary Fig. S3). Such mistranslations could misguide English-speaking users to use “Ephedra” for further knowledge searches and potentially lead them to the aforementioned misleading Wikipedia page.

To systematically tackle these challenges, we have developed an AI-driven, web-based sharing platform named ShennongAlpha for intelligent curation, acquisition, and translation of NMM knowledge (Fig. 1). The platform is also an open, collaborative, and evolving hub, which enables the global community to participate and contribute to NMM knowledge. As the first major advance in leveraging AI to empower NMM knowledge sharing, the ShennongAlpha platform currently contains knowledge of more than 14,000 Chinese NMMs. By presenting an exemplary case in this novel AI for science field, our study has not only propelled AI methodologies and applications but also will significantly accelerate the NMM research and applications for the global biomedical, pharmaceutical, and physician and patient communities.

## Results

### Systematic Nomenclature defines unique identities and critical information for NMMs

In order to accurately differentiate and identify each NMM, we introduced a Systematic Nomenclature to define unique identities and critical information for NMMs (Supplementary Texts C, D). The Systematic Nomenclature specifies each NMM with a Systematic Name, a Generic Name, and a unique NMM ID (Fig. 2). The Systematic Name is an authoritative designation for NMMs to ensure academic accuracy. It comprises four components: I. the Latin name that defines the species origin of NMM; II. the part of the organism used for medicinal purposes; III. the special description providing information about the initial preparation at the production site and other specific characteristics; and IV. the processing method to produce the NMM for medicinal use (Fig. 2a; Supplementary Fig. S4). Information for the special description (III) and the processing method (IV) is included when this critical information is necessary. The Generic Name follows a commonly used traditional name, facilitating everyday communication and aiding physicians in prescribing. The unique ID is a 4-digit base-36 code with the prefix “NMM-”, providing a unique identity for each NMM on the digital platform.

For instance, the Systematic Name for “Jing Pian Jiang Huang” (净片姜黄) is “Curcuma wenyujin Rhizome Freshly-sliced Cleaned”^25^ (Fig. 2c). This indicates that the NMM “Jing Pian Jiang Huang” is derived from the species *Curcuma wenyujin*, which utilizes the rhizome as its medicinal part, and is freshly sliced at the production site and then further processed by specific cleaning methods for medicinal use. The Generic Name uses its traditional Chinese name, “Jing-pian-jiang-huang”. The assigned unique ID is “NMM-0016”. Another example of a Systematic Name is “Ephedra sinica Stem-herbaceous Segmented and Aquafried-honey” for “Mi Cao Ma Huang” (蜜草麻黄)^26^ (Fig. 2b). It contains the species origin, medicinal part, and processing method, but no critical information for the special description.

For the species origin, some NMMs come from more than one species. In this case, the Systematic Name will incorporate the Latin names of all pertinent species to ensure clarity. For example, the Systematic Name for “Ma Huang” (麻黄) includes the species of *Ephedra equisetina*, *Ephedra intermedia*, and *Ephedra sinica* (“Ephedra equisetina vel intermedia vel sinica Stem-herbaceous”)^27^. If, in the historical record, the species origin for an NMM is not clear, the term “unspecified” will be included in the Systematic Name. For example, “Pu Gong Ying” (蒲公英) in the *Chinese Pharmacopoeia (2020 edition)* is described as an NMM using its whole plant. However, it could originate from any species within the *Taraxacum* genus, which includes thousands of species^28^. It is unclear which single or multiple species within *Taraxacum* have the medicinal effect and can be attributed to “Pu Gong Ying”. Therefore, we designate the Systematic Name for “Pu Gong Ying” as “Taraxacum unspecified Herb”^29^. For the medicinal part, different parts of the same species can potentially be used as NMMs with varying medicinal properties. Therefore, it is necessary to specify the exact part used for medicine in the Systematic Name to ensure clarity. For example, both the herbaceous stem and the root of *Ephedra sinica* can be used medicinally; hence, their Systematic Names are designated as “Ephedra sinica Stem-herbaceous”^30^ and “Ephedra sinica Root”^31^, respectively.

The information for local production and processing methods for medicinal use is also critical for NMM differentiation and identification. NMMs often exist in three types (Fig. 2b): Raw NMMs, Agricultural NMMs, and Processed NMMs. Raw NMMs are unprocessed, representing NMMs in their natural state. Agricultural NMMs are derived from Raw NMMs through initial preparation at the production site and are not intended for direct medicinal use. Agricultural NMMs are often regulated as agricultural products. Processed NMMs are further processed using specific methods for medicinal use. These processing methods or procedures often change the molecular nature and composition of the NMMs, which are critical for medicinal efficacy. Processed NMMs are typically regulated as pharmaceuticals. In countries like China^23^, Agricultural and Processed NMMs are subjected to different regulatory frameworks due to their distinct applications in agriculture and pharmaceuticals. Historically, the information for the initial preparation and processing method is not a part of the identities of NMMs. Due to this, consumers often mistakenly purchase Agricultural NMMs instead of the necessary medicinal-grade Processed NMMs. This oversight also results in regulatory challenges for industry and government (Supplementary Texts C, D).

**Fig. 3:**
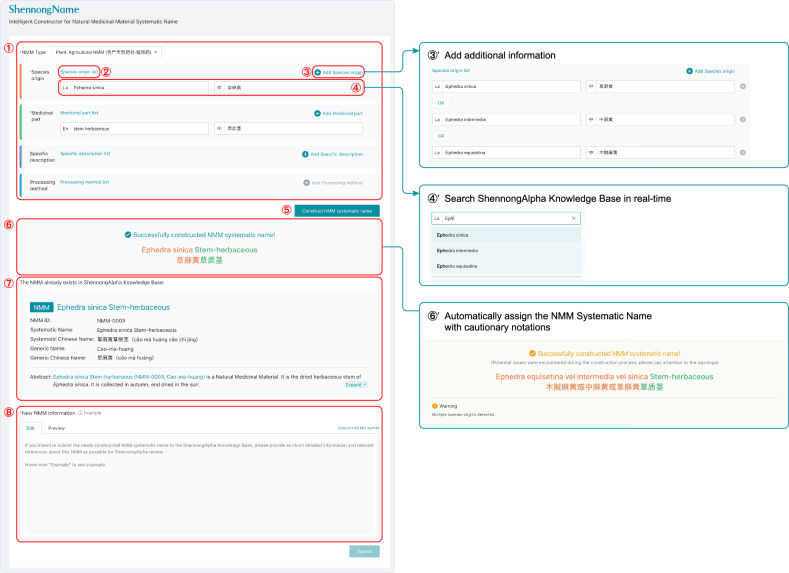
Using ShennongName to automatically construct NMM Systematic Names. Users select the NMM type and provide information for the four name components in area ①. By clicking on hyperlinks like ②, users can view entries for the four name components already cataloged in the ShennongAlpha Knowledge Base. For each name component, users can add additional information by clicking on plus buttons like ③ (③’). When users begin entering name component information in text boxes like ④, ShennongName performs a real-time search in the Knowledge Base for relevant matching entries to enable auto-completion (④’). After users have entered the necessary naming information for the NMM, they can click on the “Construct NMM Systematic Name” button (⑤), allowing ShennongName to automatically construct the Systematic Name using the algorithm. If the construction is successful, the generated information is displayed with a green background (⑥). If any issues arise during construction, the relevant information is displayed with an orange background (⑥’). For successfully constructed Systematic Names, ShennongName will also automatically perform a search for them in the Knowledge Base; if a matching NMM is found, users will be informed that the NMM is already recorded in the Knowledge Base, eliminating the need for redundant construction (⑦). After successfully constructing a new Systematic Name, if users wish to add it to the Knowledge Base, they can provide relevant details about the NMM in the textbox in area ⑧ and submit it. Once reviewed and approved by ShennongAlpha, the entry will be incorporated into the Knowledge Base.

To implement the Systematic Nomenclature for a large number of NMMs for the researcher and consumer communities, we developed an algorithm and its online web application, ShennongName, for interactive utility (Fig. 3; Supplementary Method E, https://shennongalpha.westlake.edu.cn/name). Researchers and users can contribute new NMM entries or modify existing ones through ShennongName, which will be integrated into the AI platform after review (Fig. 3 ⑧; also see below). The Systematic Nomenclature, accompanied by the ShennongName algorithm and application, provides a standardized, unambiguous, and efficient platform for differentiating and identifying NMMs.

### ShennongAlpha: an AI-driven sharing and collaboration platform for intelligent curation, acquisition, and translation of NMM knowledge

Following Systematic Nomenclature, we embarked on a comprehensive process to collect and curate NMM knowledge (for detailed data processing steps, refer to Supplementary Method G). The initial phase of the endeavor described here involved gathering a total of 14,256 Chinese NMM entries (Supplementary Table S2), as they are the largest collection of NMMs. These NMM entries were sourced from authoritative data sources, especially the 2020 and 2015 editions of the *Chinese Pharmacopeia*^23,32^, and the Chinese Medicinal Information Platform^33^. After collecting these NMM entries, we applied a series of data processing steps to extract and standardize the critical information associated with the four components of the Systematic Name. For example, to standardize species origin, we developed an automated data processing program that retrieves the correct species names from the authoritative “SP2000 China Node” species database^34^, ensuring both accuracy and reliability. Then, we applied the Systematic Nomenclature and ShennongName to compute the relevant information and generated the Systematic Name, Generic Name, and ID for each NMM. Additionally, to provide a knowledge base with richer internal relationships and more comprehensive knowledge, we annotated the hierarchical parent-child relationships among NMMs; we also collected and organized information related to the ingredients contained in NMMs, as well as their related targets and diseases, from other traditional Chinese medicine databases such as HERB^35^ and HIT^36^. Finally, all this collected knowledge was digitalized, structured, reviewed, and curated into an NMM Knowledge Base (Supplementary Fig. S5). To share this valuable Knowledge Base with worldwide users and facilitate its utilization and collaboration, we developed an AI-powered web platform named “ShennongAlpha” for the efficient and intelligent curation, acquisition, and translation of related NMM knowledge.

ShennongAlpha, named after the pioneer of Chinese medicine, is an advanced AI platform characterized by its comprehensive integration. It combines ShennongName with a hexa-domain modular system to establish its foundational architecture. This architecture encompasses a custom-designed NMM Web, a Knowledge Base, a Search Engine, and a Large Language Model (LLM) system, along with an application for chat-based human-machine interaction and an application for standardized machine translation (Fig. 1b).

We believe that the cornerstone of an excellent AI platform lies in its user-friendliness and accessibility. With this in mind, ShennongAlpha has been crafted to ensure that even users with no background in AI, programming, or algorithms can easily navigate and utilize the platform. In the following sections, we will illustrate how users can effortlessly and intelligently curate, acquire, and translate NMM-related knowledge through the straightforward process of accessing and harnessing the power of ShennongAlpha via the Web. The sophisticated technical details, implementations, and algorithms that underpin ShennongAlpha are discussed extensively in the “Materials and methods” and “Supplementary Information” sections, showcasing the substantial work and expertise that have gone into developing such an accessible yet advanced platform.

### Users access the curated NMM knowledge on ShennongAlpha

To allow users to access and interact with the curated NMM knowledge easily, we launched the ShennongAlpha Web (https://shennongalpha.westlake.edu.cn). The Web serves as the primary portal for users to engage with ShennongAlpha, distinguished by its modern and user-centric web design, allowing a consistently superior user experience regardless of device screen dimensions (Fig. 1c). The Web was crafted to be bilingual, toggling seamlessly between English and Chinese, thereby catering to both Chinese and international users (Fig. 1d, e). The bilingual feature of ShennongAlpha, for the first time, enables the international biomedical, pharmaceutical, physician, and patient communities to access the knowledge of Chinese NMMs, as this knowledge has largely been limited to the local community in the past. The standardized and structured Knowledge Base in ShennongAlpha is critical for correctly understanding and utilizing NMM knowledge. Therefore, ShennongAlpha is particularly significant for future research and application of NMMs.

To access the NMM knowledge through the ShennongAlpha Web, users can easily browse different NMM entries through distinct knowledge pages. The URLs of these pages are structured by unique NMM IDs, as exemplified below: https://shennongalpha.westlake.edu.cn/knowledge/<nmm-id>

For instance, “Ma Huang” is assigned the NMM ID: “NMM-0006”, leading to the knowledge page: https://shennongalpha.westlake.edu.cn/knowledge/nmm-0006

**Fig. 4:**
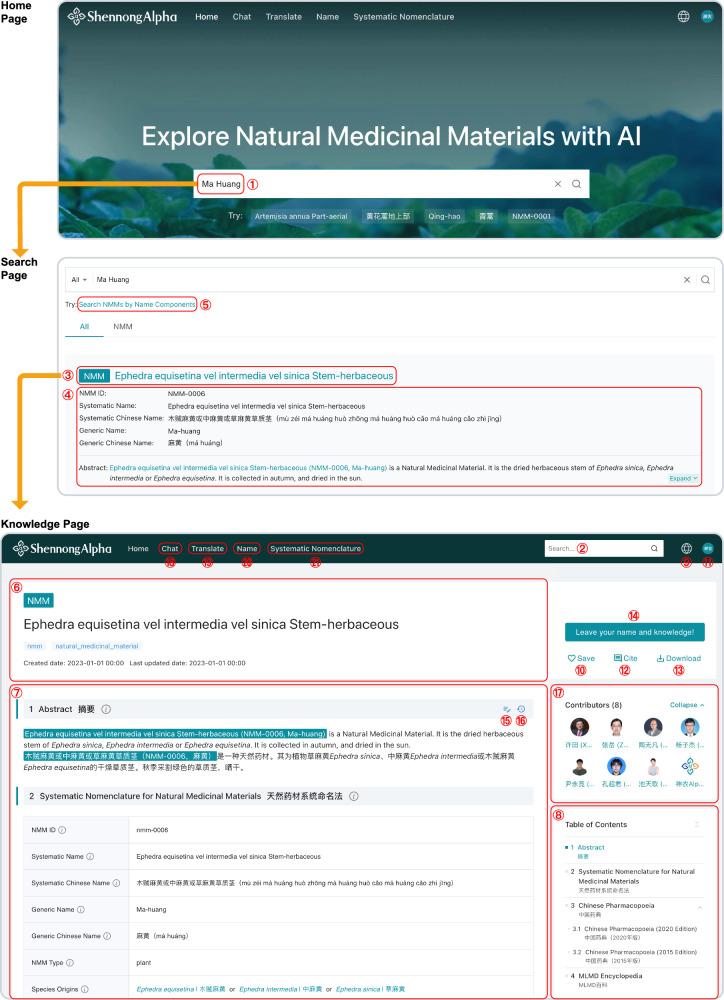
Browsing NMM knowledge on the ShennongAlpha Web. Users can initiate their exploration of NMM knowledge by using the search bar located either on the homepage (①) or atop other pages (②). Post-search, users are directed to the search page where they can glance through the title (③) and summarized information (④) of each NMM entry to ascertain its relevance. The search page also offers an advanced search mode (⑤), allowing users to quickly find NMMs that share common components of their Systematic Names (Supplementary Fig. S6). By clicking on the title of an entry, users are navigated to a detailed knowledge page dedicated to that specific NMM. The header of this knowledge page (⑥) displays the Systematic Name of the NMM, while the main content area (⑦) is organized in a structured, section-by-section layout (Supplementary Figs. S7, S8). The “Table of Contents” sidebar (⑧) enables swift navigation between sections. To facilitate cross-language accessibility for global users, the Web offers four display modes (⑨): Bilingual (Chinese-English), Bilingual (English-Chinese), Chinese only, and English only (Supplementary Fig. S9). The “Save” button (⑩) allows users to bookmark the knowledge page to their user profile page (⑪; Supplementary Fig. S10). To encourage academic references, the “Cite” button (⑫) offers citation formats in styles such as APA, MLA, GB/T 7714-2015, and BibTeX. The “Download” button (⑬) enables users to download the knowledge page’s content in JSON format. Furthermore, with the “Leave your name and knowledge!” button (⑭), users can propose new or revised NMM-related knowledge. Contributions can also be made directly via the “Edit Content” button (⑮), allowing users to modify the content of each section and submit it to ShennongAlpha for review (Supplementary Fig. S12). To view past modifications, the “Show Edit History” button (⑯) provides access to all historical changes. Approved user contributions are then integrated into the ShennongAlpha Knowledge Base, and contributors are recognized and acknowledged in the “Contributors” area (⑰), where their usernames and avatars are displayed. Users can navigate to the ShennongChat (⑱; Fig. 5), ShennongTranslate (⑲; Fig. 6), and ShennongName (⑳; Fig. 3) applications in ShennongAlpha, as well as the detailed rules of the Systematic Nomenclature for NMMs (), directly from the homepage or through the header navigation bar on any page of the Web.

Besides accessing information via direct URLs, users can leverage a search function. This feature is prominently available within a search box on the homepage (Fig. 4 ①) or at the top navigation bar of every other page (Fig. 4 ②), all powered by the ShennongAlpha Search Engine. Upon executing a search, results are presented on a dedicated search page, where each entry contains pertinent details, including the NMM ID, Systematic Name, Generic Name, and brief introductory abstract (Fig. 4 ④). This information aids users in discerning the relevance of their search results.

To further enhance the efficiency of searching for NMMs, we developed an advanced search module called “Search NMMs by Name Components” (Fig. 4 ⑤; also accessible via the following URL: https://shennongalpha.westlake.edu.cn/search-nmms-by-name-components). Using “Search NMMs by Name Components”, users can quickly find NMMs in the Knowledge Base that share common characteristics and then obtain relevant knowledge. For example, by setting the species origin to “Ephedra sinica”, users can rapidly find NMMs such as “Ephedra sinica Stem-herbaceous”, “Ephedra equisetina vel intermedia vel sinica Stem-herbaceous”, and “Ephedra sinica Root” (Supplementary Fig. S6a). If users further constrain the search by specifying both the species origin as “Ephedra sinica” and the medicinal part as “stem herbaceous”, the search results narrow down to “Ephedra sinica Stem-herbaceous” and “Ephedra equisetina vel intermedia vel sinica Stem-herbaceous”, excluding “Ephedra sinica Root” (Supplementary Fig. S6b), since the latter’s medicinal part is root, which does not meet the search criteria.

Knowledge pages within ShennongAlpha utilize a hierarchical tree structure, with content organized into sequenced sections and allowing for nested subsections (Fig. 4 ⑥, ⑦; Supplementary Figs. S7, S8). The “Table of Contents” in the sidebar navigation facilitates users in swiftly pinpointing and navigating to distinct knowledge sections (Fig. 4 ⑧).

In our bid to ensure knowledge accessibility across language barriers, we designed a new text format known as Multilingual Markdown (MLMD) (Supplementary Method F) for curating multilingual NMM knowledge. This approach allows ShennongAlpha to universally support four distinct language display modes: Chinese-English (zh-en), English-Chinese (en-zh), Chinese (zh), and English (en) (Supplementary Fig. S9). Users can effortlessly switch between these modes using the language button (Fig. 4 ⑨). The functionalities of these modes are detailed below:

- zh-en: The Platform interface is in Chinese, with the Knowledge Base content rendered in a bilingual Chinese-English format.
- en-zh: The Platform interface is in English, with the Knowledge Base content rendered in a bilingual English-Chinese format.
- zh: Both the Platform interface and the Knowledge Base content are exclusively in Chinese.
- en: Both the Platform interface and the Knowledge Base content are exclusively in English.

Furthermore, within knowledge pages, users are afforded the option to save (Fig. 4 ⑩) the page to their user profile (Supplementary Fig. S10); cite (Fig. 4 ⑫) it; or download (Fig. 4 ⑬) the content of the page. The downloadable content is structured in JSON format, enabling users to programmatically utilize the original NMM knowledge for advanced processing and applications.

### Users contribute NMM knowledge through ShennongAlpha

Unlike traditional knowledge platforms, ShennongAlpha is uniquely designed as an open and collaborative hub. It invites global users to contribute their invaluable knowledge and findings about NMMs. On every knowledge page, users can find a prominent “Leave your name and knowledge!” button (Fig. 4 ⑭). This feature encourages users to submit new information or propose modifications to existing content. Each section of NMM knowledge, such as “Abstract”, “Chinese Pharmacopoeia”, and “MLMD Encyclopedia” is accessible for enhancement or amendment through the “Edit Content” button (Fig. 4 ⑮). These user-generated modifications will be integrated into the Knowledge Base upon review and approval by ShennongAlpha (Supplementary Fig. S12). This feature will continually advance the Knowledge Base by leveraging and empowering the community.

Furthermore, the “Show Edit History” button (Fig. 4 ⑯) provides a tracking method and a transparent view of all historical modifications. Notably, once a user’s contribution is included in the Knowledge Base, it will be acknowledged. Their names and avatars are prominently displayed under the “Contributors” section (Fig. 4 ⑰), not only as a token of appreciation but also as a badge of honor for their collaborative efforts. By pooling the collective expertise of the global NMM community, ShennongAlpha will rapidly evolve, driven by shared knowledge, collaborative spirit, and global community dedication.

### Chat-based NMM knowledge acquisition on ShennongAlpha

**Fig. 5:**
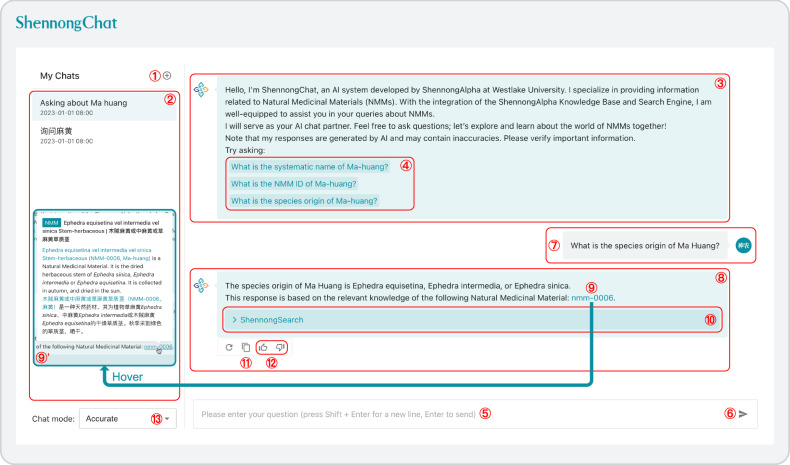
Chat-based NMM knowledge acquisition through ShennongChat. Users can initiate a new chat by clicking the plus button (①). The chat history area (②) retains previous chat sessions, allowing users to navigate between historical chat sessions or delete unwanted chat sessions if needed. Within each chat session, the guidance section (③) highlights the unique features and capabilities of ShennongChat. To quickly experience the chatting features, users can select from a set of sample questions in the ④ area. By inputting a question in the chat box (⑤) and hitting the send button (⑥), the user’s question (⑦) is relayed to ShennongChat for a response. In formulating its response, ShennongChat integrates the ShennongAlpha Search Engine to retrieve standardized knowledge about the NMM mentioned in the user’s question from the ShennongAlpha Knowledge Base, providing a retrieval-augmented response (⑧). ShennongChat’s responses indicate the relevant NMM IDs of the referenced NMM knowledge (⑨). When users hover over the NMM ID, ShennongChat displays a tooltip with a summary of the knowledge about that NMM (⑨’). Users can quickly navigate to the knowledge page of the relevant NMM by clicking the title within the tooltip. Users can also expand the search dropdown (⑩) for details about the search results. The copy button (⑪) enables users to duplicate the text of ShennongChat’s response. Feedback on the responses (either positive or negative) can be provided by the user (⑫), which helps in improving the quality of ShennongChat’s responses. Depending on their preference, users can toggle between ShennongChat’s chat modes: accurate or fast (⑬).

In addition to the structured knowledge pages for a comprehensive overview, ShennongAlpha also heralds a major shift in NMM knowledge acquisition with the introduction of its AI-powered Chat application (ShennongChat, Fig. 5). By integrating the advanced LLM system into the Chat application, users are empowered with chat-based acquisition for NMM knowledge. Users can pose their questions to the Chat in natural language, eliminating the need for a technical background or specific search syntax. Leveraging the power of the Search Engine, which is enhanced with our newly developed Coreference-based Graph Search (CGS) algorithm (Supplementary Fig. S13), and employing a Retrieval-Augmented Generation (RAG) architecture, the Chat automatically searches the Knowledge Base in real time and articulates the retrieved knowledge into natural language responses for users (Supplementary Fig. S17).

Users can access the Chat via the following URL on the ShennongAlpha Web: https://shennongalpha.westlake.edu.cn/chat.

The Chat supports multi-turn conversations. For example, a user poses the following question (Fig. 5 ⑦):

What is the species origin of Ma Huang?

Then, the Chat interprets the user’s intent, automatically searches the Knowledge Base, and provides the following response (Fig. 5 ⑧):

The species origin of Ma Huang is Ephedra equisetina, Ephedra intermedia, or Ephedra sinica.

This response is based on the relevant knowledge of the following Natural Medicinal Material: nmm-0006.

It is noteworthy that this answer aptly highlights the multi-species character of “Ma Huang”, distinguishing it from the response given by GPT-4 (Supplementary Fig. S2).

Furthermore, as an AI conversational system in the scientific domain, it is crucial to provide professional and scientifically rigorous responses. To this end, the Chat has been specially designed in three aspects to enhance the interpretability of responses:

1. User guidance: in the introductory section of the Chat (Fig. 5 ③), we provide appropriate prompts to inform users that AI-generated responses may contain inaccuracies and that they should verify important information.
2. Transparency of information sources: when the Chat’s responses utilize relevant knowledge retrieved from the Knowledge Base, it declares the associated NMM IDs in the answers and highlights these IDs (Fig. 5 ⑨). Search results are embedded within the Chat’s answers in a collapsible format, allowing users to expand these sections for deeper insights into the background knowledge supporting the answers (Fig. 5 ⑩).
3. Interactive knowledge exploration: when users hover the cursor over an NMM ID (Fig. 5 ⑨), the Chat interface automatically displays a tooltip with a summary of the relevant NMM knowledge (Fig. 5 ⑨’). By clicking on the tooltip, users can quickly navigate to the corresponding NMM knowledge page. This feature enables users to efficiently explore related NMM knowledge and facilitates quick verification of important information contained in the AI’s responses.

Moreover, leveraging the advanced intelligence of the LLM system, the Chat supports interactions in multiple languages. Users can specify their preferred response style, whether in a different language or a particular structural format (Supplementary Fig. S18). This adaptability enhances the overall user experience, broadens knowledge accessibility, and promotes global knowledge sharing.

### Standardized and interpretable translation of NMM knowledge on ShennongAlpha

**Fig. 6:**
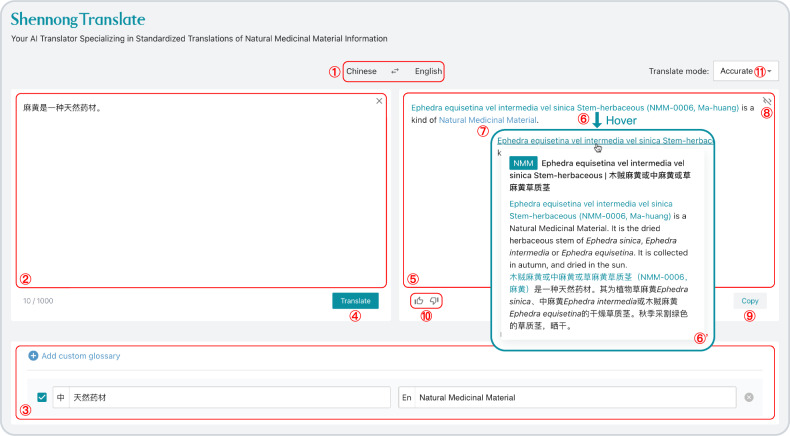
Standardized and interpretable translation of NMM text through ShennongTranslate. Users can toggle between Chinese and English translation directions using ①. After inputting the NMM text to be translated in the text entry area (②) and adding any user-customized glossary (③, optional), users can click the “Translate” button (④) to submit the text for translation. The user-customized glossaries are saved, and users can add/delete, activate/deactivate these glossaries as needed, allowing ShennongTranslate to become increasingly tailored to their translation preferences. During the translation process, ShennongTranslate automatically uses the ShennongAlpha Search Engine to identify NMM terms in the text and then retrieve their Primary Terms and standardized translations from the ShennongAlpha Knowledge Base. In the translation results (⑤). standardized translations of NMM terms (⑥) and user-customized terms (⑦) are highlighted in teal and blue, respectively. Hovering over a standardized translation of an NMM term (⑥) prompts ShennongTranslate to display a tooltip (⑥’) containing its introductory abstract; clicking on the tooltip directs users to the knowledge page of the NMM. The button ⑧ allows users to toggle between plain text and MLMD source code displays. In either display mode, users can copy the content by clicking the “Copy” button (⑨). Feedback on the translation (either positive or negative) can be provided by the user (⑩), helping to improve ShennongTranslate’s performance. Depending on their preference, users can toggle between ShennongTranslate’s translate modes: accurate or fast (⑪).

Another important factor in NMM research and applications is language barriers, as much of the accumulated experience and knowledge is often recorded in a monolingual format. Therefore, a key feature of the ShennongAlpha platform is an AI-based standardized and interpretable Translate application for NMM knowledge (ShennongTranslate, Fig. 6). The Translate application is underpinned by an AI translation algorithm that we developed (Neural Machine Translation based on Coreference Primary Term, NMT-CPT, Supplementary Fig. S20). It addresses language barriers and also prevents semantic drift caused by non-standardized translations.

Users can access the Translate via the following URL on the ShennongAlpha Web: https://shennongalpha.westlake.edu.cn/translate.

The Translate upholds consistency in translations from Chinese to English or vice versa. For example, a user submits the following text to be translated (Fig. 6 ②):

麻黄是一种天然药材。

Then, the Translate automatically searches the Knowledge Base for the related NMM entities in the text, retrieves their standardized translations, and generates the final translation for the sentence to ensure it is standardized and interpretable for both language and NMM knowledge requirements (Fig. 6 ⑤):

Ephedra equisetina vel intermedia vel sinica Stem-herbaceous (NMM-0006, Ma-huang) is a kind of Natural Medicinal Material.

Here, “麻黄” is translated using its standardized NMM nomenclature: “Ephedra equisetina vel intermedia vel sinica Stem-herbaceous (NMM-0006, Ma-huang)”. Similarly, “天然药材” incorporates a user-defined glossary as “Natural Medicinal Material”.

Uniquely, within the Translate’s interface, distinct color highlights are applied to both the standardized translation “Ephedra equisetina vel intermedia vel sinica Stem-herbaceous (NMM-0006, Ma-huang)” (Fig. 6 ⑥) and the user-defined glossary “Natural Medicinal Material” (Fig. 6 ⑦). This offers users instant clarity on specialized terms, thus enhancing the standardization and interpretability of the translation results. Even more significantly, the Translate automatically identifies the NMM associated with the standardized translation, enabling access to its knowledge page on ShennongAlpha. By merely hovering over the standardized translation term, users are presented with a bilingual summary tooltip (Fig. 6 ⑥’). Clicking the term within the tooltip directly navigates to its detailed knowledge page. This interactive approach not only provides users with standardized translations but also fosters further exploration of related knowledge, thereby enhancing understanding. It extends the traditional role of the Translate beyond mere translation, transforming it into an application for knowledge exploration, learning, and sharing.

## Discussion

Global knowledge sharing is particularly important for specialized fields such as NMMs, which are traditionally rich with historical records documented in local texts and formats. The emergence of large language models (LLMs) offers unique opportunities to facilitate knowledge sharing. However, it also raises concerns that incorrect information could be spread by advanced AI technologies at unprecedented rates. Furthermore, LLMs trained with misinformation could undermine knowledge integrity in specialized fields. Therefore, it is urgent to correctly curate and translate knowledge in such fields, thereby enabling intelligent acquisition and further dissemination through AI methods.

A critical issue for sharing NMM knowledge and promoting research and applications is the lack of standardized and Systematic Nomenclature. This deficiency hampers accurate differentiation and identification of NMMs, and obstructs effective communication and collaboration within the field. In comparison, the pharmaceutical sector has established Systematic Nomenclature for chemical drugs to ensure accuracy and consistency in chemical identification and research. Specifically, the chemical systematic naming system includes a systematic name for accurately describing the chemical structure of each drug (the International Union of Pure and Applied Chemistry nomenclature^37^); a generic name for convenient usage (the International Nonproprietary Names^38^); and a unique identification number for digital platforms (the Chemical Abstracts Service registry number^39^, the PubChem Compound ID number^40^).

In this study, we have introduced a Systematic Nomenclature for NMMs. The Systematic Name describes an NMM by its species origin, medicinal part, special description, and processing method. The Generic Name provides convenience for NMM usage and also observes local tradition. The NMM ID facilitates use on digital platforms. Therefore, for the first time, the Systematic Nomenclature provides a standardized system that uniquely differentiates and identifies each NMM. Using the Systematic Nomenclature and its companion ShennongAlpha platform (see below), we have assigned identities for more than 14,000 Chinese NMMs and curated their related knowledge in the Knowledge Base. The Systematic Nomenclature could also be applied to additional NMMs from other regions of the world.

To systematically empower NMM knowledge sharing, we have developed the AI-driven platform ShennongAlpha. First, ShennongAlpha automates the implementation of the Systematic Nomenclature for NMMs through the tailored algorithm and application. Second, to address language barriers, all knowledge entries in ShennongAlpha are curated bilingually in Chinese and English, making them accessible to both Chinese and global users. This is supported by our Translate application, which provides standardized and interpretable translation for the cross-lingual sharing of NMM knowledge. Although the current platform primarily supports English and/or Chinese interface displays and bidirectional text translation, the MLMD syntax and the NMT-CPT algorithm allow the platform to be easily expanded for multilingual presentation and translation into other languages. Third, by leveraging the LLM/RAG-based architecture, ShennongAlpha also offers the Chat application for NMM knowledge acquisition. Its conversational interface enables users to obtain standardized NMM knowledge in an interactive, chat-based manner. While existing RAG methods^41–43^ are typically designed for general-domain question-answering, our NMM-specific Q&A scenarios present unique challenges that demand high standards of academic accuracy and explainability. To address these, we implemented three specialized approaches: 1. customized RAG over the dynamically updated, standardized NMM Knowledge Base; 2. the CGS search algorithm to accurately recognize and extract NMM entities from user queries, facilitating precise retrieval of corresponding NMM knowledge; 3. the integration of responses with the retrieved knowledge (Fig. 5 ⑩) and explicitly reference the relevant NMM IDs (Fig. 5 ⑨), providing interactive features where users can hover to display knowledge summaries and click to navigate directly to detailed pages (Fig. 5 ⑨’). These approaches not only enhance the accuracy and explainability of the Chat application but can also be integrated into existing RAG methods, offering valuable insights for developing future domain-specific RAG frameworks. Fourth, ShennongAlpha has introduced a unique collaboration mechanism for updating NMM knowledge. By encouraging global users to contribute their valuable new information and findings about NMMs, the Knowledge Base within ShennongAlpha will continue to grow. This represents not only a mechanism for sharing new knowledge but also fosters collaboration within the global NMM community. Finally, despite its sophisticated architecture, algorithms, and implementation, ShennongAlpha offers an integrated, user-friendly interface through the ShennongAlpha Web. This design skillfully conceals the underlying complexities, making it easily accessible for users.

Despite the significant progress made with the current version of the ShennongAlpha platform, there are still limitations. First, although we have included over 14,000 NMMs, making it the largest NMM knowledge base to date, the sources are mainly Chinese NMMs. Since NMMs are widely used globally, we plan to include more NMMs from other countries and regions in the future to enhance the platform’s global applicability. Second, the types of standardized and structured knowledge in the ShennongAlpha Knowledge Base are still relatively limited. Currently, the Knowledge Base mainly includes text-based information and some important data related to NMMs, such as ingredients, related targets, and diseases (Supplementary Fig. S8). However, it has not yet incorporated experimental data, which are useful in the field of NMMs because they provide direct evidence for knowledge and discoveries. The absence of experimental data is primarily due to two factors: 1. experimental data are often unstructured; 2. a large number of new papers providing experimental data and insights about NMMs are published globally every day. Therefore, timely and effectively standardizing, structuring, and integrating the latest experimental data into the Knowledge Base is a significant challenge. In the future, we plan to develop more advanced AI methods to achieve this and ultimately build an encyclopedic resource in the field of NMMs.

In summary, ShennongAlpha epitomizes the exciting fusion of AI with NMM knowledge sharing in the novel AI for science field. ShennongAlpha’s hexa-domain modular architecture (Fig. 1b) also offers a model for knowledge sharing in other specialized fields. ShennongAlpha also provides the world’s largest NMM Knowledge Base with standardized and curated knowledge, serving as a unique resource for NMM researchers, physicians, patients, and users, as well as for future LLM training. We believe that as AI increasingly emerges as a primary knowledge provider in scientific research^44^, our platform will significantly promote global knowledge sharing and collaboration in the NMM field, ultimately benefiting global health and human well-being.

## Materials and methods

### Systematic Nomenclature for NMMs with its algorithm and application

Detailed rules for Systematic Nomenclature for NMMs (SNNMM) can be found in Supplementary Texts C, D.

SNNMM Algorithm (SNNMMA) is coded in Python, with its algorithm stringently following the rules of SNNMM. It is open-source, and the source code is available on GitHub: https://github.com/shennong-program/shennongname. The associated Python package for SNNMMA is published and released on PyPI: https://pypi.org/project/shennongname.

For a concise overview of the data structure and algorithms of SNNMMA, please refer to Supplementary Method E.

### Multilingual Markdown

MLMD is a newly designed lightweight markup language explicitly tailored for managing multilingual text.

The essential syntax of MLMD can be found in Supplementary Method F.

The MLMD HTML parser is built using TypeScript. It is open-source, with the source code accessible on GitHub: https://github.com/shennong-program/mlmd. The associated TypeScript package is published and released on npm: https://www.npmjs.com/package/mlmd.

### ShennongAlpha Knowledge Base (ShennongKB)

ShennongKB represents the core knowledge base within the ShennongAlpha and resides in the 5th layer known as the knowledge base layer (Fig. 1b).

ShennongKB, built on the MongoDB document-oriented database, integrates and organizes a diverse dataset. The current version of ShennongKB predominantly sources its data from the 2020^23^ and 2015^32^ editions of the *Chinese Pharmacopoeia*, and the Chinese Medicinal Information Platform^33^. This NMM-associated data is organized into several interrelated collections, including SNNMM, Text, Knowledge, Glossary, and Relation (Supplementary Fig. S5). This organization follows a thorough process of collection, structuring, standardization, and review, all carried out by our ShennongAlpha team. For detailed methods on the collection, processing, and curation of the knowledge included in ShennongKB, please refer to Supplementary Method G.

### ShennongAlpha Web

The ShennongAlpha Web serves as the principal interface for users with the ShennongAlpha, situated within ShennongAlpha’s 1st layer, the web and user interaction layer (Fig. 1b).

Embracing a responsive design approach, ShennongAlpha Web assures users of a uniform and superior user experience on screens of any dimension. ShennongAlpha Web is built on TypeScript, employing Next.js as its principal framework. It amalgamates the ShennongAlpha Knowledge Base and Search Engine. Additionally, it incorporates applications such as ShennongName, ShennongChat, and ShennongTranslate. ShennongAlpha takes advantage of a microservices architecture, facilitating the decoupling of diverse service units for enhanced manageability and extensibility. The backend for applications like ShennongName, ShennongChat, and ShennongTranslate is developed using Python, leveraging the Flask or FastAPI framework. These applications interface with ShennongAlpha through APIs that adhere to RESTful standards, fostering overall system stability and maintainability. For deployment, ShennongAlpha exploits Docker for container orchestration and Docker Compose technology for integrating and managing multiple containerized applications.

### ShennongAlpha Search Engine (ShennongSearch)

ShennongSearch (Supplementary Fig. S13) is the core of knowledge retrieval in the ShennongAlpha, located in the 4th layer referred to as the search engine layer (Fig. 1b).

ShennongSearch can be seen as a data highway within the ShennongAlpha. To accommodate a wide array of queries and guarantee efficient and precise information retrieval, ShennongSearch is customized and designed with three advanced search methods: CGS (Supplementary Fig. S13a), vector search (Supplementary Fig. S13b) and full-text search (Supplementary Fig. S13c). Each search method possesses distinct strengths and can be employed individually or collectively to yield accurate and thorough search results, thereby enhancing the overall usability of ShennongSearch. The three advanced search methods of ShennongSearch are primarily applied in the knowledge search process of ShennongChat. In the publicly accessible ShennongChat, knowledge searches apply the CGS method, because it excels in accurately searching for NMM terms (Supplementary Figs. S14, S15; Supplementary Method H).

#### Coreference-based Graph Search

The CGS algorithm (detailed in Supplementary Method H), designed and developed by this project, centers on dispatching relationships between different terms within the relation collection of ShennongKB, automatically constructing a Coreference Primary Term Graph (CPTG) (Supplementary Fig. S13a). CPTG essentially operates as a directed acyclic graph. Given a term/named entity, graph search within CPTG locates its corresponding Primary Term. This Primary Term then allows querying its corresponding standard information within other collections in ShennongKB (such as Knowledge or Translation). This method is especially useful when standardizing queries. We construct the CPTG using the Python networkx package^45^. The associated Python implementation for CGS is available on GitHub (https://github.com/shennong-program/pycgs) or PyPI (https://pypi.org/project/pycgs).

#### Vector search

The method utilizes a vector-based approach, transforming search queries and documents within ShennongKB into vector embeddings (Supplementary Fig. S13b). A unique strength of this method lies in its ability to accept entire sentences or paragraphs as search input. By converting this text into vector space through embedding, the semantic essence is captured within a vector representation. Subsequently, by evaluating the similarity between the query text vector embedding and the archived text vector embeddings within ShennongKB, information that is semantically relevant to the query text can be swiftly identified and retrieved. We use the text-embedding-3-small model for vectorizing NMM-related texts because it provides a suitable balance between embedding quality and reduced storage/computational requirements (Supplementary Fig. S15. The dataset and code for this evaluation are available on GitHub: https://github.com/shennong-program/shennongllm-evals). Each text within ShennongKB is preprocessed with text-embedding-3-small, generating a 1536-dimensional vector embedding. These embeddings and their original texts are then stored within ShennongKB. Upon each new search, the query text is processed with text-embedding-3-small to generate a new vector embedding. Then, the search engine uses cosine similarity to measure the similarity between this new embedding and the vector embeddings archived in ShennongKB, returning the text corresponding to the most similar vector embedding as the search result. ShennongSearch utilizes MongoDB’s $vectorSearch operator for its vector search functionality.

#### Full-text search

This method is rooted in full-text fuzzy searching, using a tokenization and inverted index process (Supplementary Fig. S13c). Operating by breaking down documents and search queries in ShennongKB into individual words or “tokens”, this method allows efficient searching and retrieval of pertinent information, accommodating approximate matches, variations in phrasing, typos, misspellings, or alternate spellings. This method notably enhances the system’s flexibility and user-friendliness by leveraging the tokenization and inverted index. ShennongSearch utilizes MongoDB’s $text and $search operators for its full-text search functionality. Documents within MongoDB are tokenized using Jieba^46^, after which a full-text inverted index is constructed. During queries, the text to be searched is also tokenized using Jieba before the full-text search is executed.

### ShennongAlpha Large Language Model (ShennongLLM)

Situated within the 3rd layer, the AI layer, ShennongLLM acts as the pivotal AI hub for the ShennongAlpha (Fig. 1b). Built on a foundation of LLMs, the hub is designed for adaptability, supporting both open-source and proprietary LLMs like Llama 2/3^47^, GPT-4/4o/4o-mini^48^, Qwen^49,50^, among others. This versatility is accessible either through local deployment or API calls.

In the current version of LLM-powered applications within ShennongAlpha, including ShennongChat and ShennongTranslate (detailed below), we did not retrain the LLMs. The primary reason is that ShennongAlpha’s unique design allows users to contribute and modify knowledge, leading to daily updates in the information stored in ShennongKB. Continually retraining LLMs to incorporate the latest NMM-related knowledge would be impractical in terms of both cost and timeliness. Therefore, these applications adopt a RAG architecture^51,52^, integrating LLMs with the Search Engine (ShennongSearch) and the Knowledge Base (ShennongKB). This integration enables ShennongChat and ShennongTranslate to fully utilize the latest standardized knowledge and translations recorded in ShennongKB. An additional advantage of this design is that, as both open-source and proprietary LLMs rapidly advance in capability, the ShennongLLM layer can flexibly integrate the latest LLMs into the ShennongAlpha platform. This ensures that ShennongAlpha’s AI capabilities continue to improve, keeping the platform at the forefront of AI technology.

In the current version of ShennongChat and ShennongTranslate, the “accurate” and “fast” modes use proprietary Qwen series LLMs (e.g., qwen-max, qwen-turbo). If the user opts to switch to open-source LLMs (Supplementary Fig. S11 ⑦’), these modes will then utilize LLMs from the Qwen open-source series (e.g., qwen2.5-14b-instruct, qwen2.5-7b-instruct).

### ShennongAlpha Chat application (ShennongChat)

Acquiring knowledge through direct chat with LLMs heralds a transformative paradigm in information retrieval. However, LLMs often produce inaccurate or spurious outputs, especially in unfamiliar domains^53–55^. Given the fact that NMM knowledge had not been standardized prior to this study, the standardized knowledge we curated was unavailable for training LLMs. Therefore, even state-of-the-art LLMs like GPT-4 tend to provide misleading responses (Supplementary Fig. S2).

We further illustrated this limitation through an intuitive experiment. By constructing a series of factual questions about NMMs, such as “What is the NMM ID of Ma-huang?”, we asked the advanced model GPT-4o to answer them. GPT-4o might respond, “The NMM ID of Ma-huang is NMM0001722”. However, this answer is incorrect; the correct NMM ID of Ma-huang in ShennongKB is “NMM-0006”. We tested GPT-4o with 4000 similar questions and found its accuracy was 0% (Supplementary Fig. S16). The fundamental reason is that concepts and standardized knowledge like NMM IDs are being introduced for the first time in our study and did not exist in any database prior to our research. Since GPT-4o was trained on data up to October 2023 and ShennongAlpha was publicly released after this date, GPT-4o could not have learned this new standardized knowledge and naturally cannot provide correct answers to these factual questions.

However, when we provided the LLM with the relevant background knowledge needed to answer each question, its accuracy significantly increased. For example, when the question was “What is the NMM ID of Ma-huang?”, we injected the knowledge related to Ma-huang’s NMM ID into the conversation context. With this background knowledge support, GPT-4o’s accuracy rapidly increased to 99%. Even less capable proprietary or open-source models like GPT-4o-mini, Qwen-Turbo, Qwen2 7B, and Llama 3.1 8B achieved accuracies of 98%, 99%, 99%, and 99%, respectively (Supplementary Fig. S16). The dataset and code for both this test and the previous one without background knowledge are available on GitHub: https://github.com/shennong-program/shennongllm-evals).

Recognizing this, we adopt the RAG architecture in the design of the ShennongChat, substantially enhancing the accuracy of LLM responses.

ShennongChat utilizes the prompt chain technique^41,56^, a method that uses a step-by-step process to ensure the LLM’s responses to user queries are based on retrieved standardized NMM knowledge. This technique enhances the relevance and reliability of the knowledge acquired during chats.

For illustrative purposes, let’s examine the user query mentioned in the “Results” section (Fig. 5; Supplementary Fig. S17):


What is the species origin of Ma Huang?


Upon receiving a query, ShennongChat proactively uses ShennongSearch to conduct a knowledge search. This process involves dispatching ShennongSearch to retrieve relevant standardized knowledge from ShennongKB. From this search, we glean:


nmm_id: nmm-0006species_origins: Ephedra equisetina, or, Ephedra intermedia, or, Ephedra sinica


This contextual information is then amalgamated with the user’s query and submitted to ShennongLLM to formulate an accurate response:


The species origin of Ma Huang is Ephedra equisetina, Ephedra intermedia, or Ephedra sinica.This response is based on the relevant knowledge of the following Natural Medicinal Material: [[nmm-0006]].


To ensure the professionalism and academic rigor of ShennongChat, ShennongChat’s responses explicitly state the NMM IDs of the referenced NMM knowledge. The syntax “[[…]]” is a specialized notation of MLMD, which the ShennongChat interface can automatically parse to highlight the enclosed NMM IDs (Fig. 5 ⑨). Furthermore, when users hover over an NMM ID, the interface automatically displays a tooltip summarizing the corresponding NMM knowledge page (Fig. 5 ⑨’).

In addition, we have improved ShennongChat’s academic rigor through prompt engineering in the following ways:

To ensure that ShennongChat appropriately informs users when its responses lack support from relevant background knowledge (Supplementary Fig. S19), we added the following system prompt:


To ensure scientific accuracy and rigor in responses, if ShennongSearch does not retrieve relevant knowledge that directly supports answering the user's latest question, begin your response with the following statement: I could not find relevant knowledge from the search to directly answer your question, but I will do my best to answer it based on my internal knowledge.


To ensure that ShennongChat generates consistent responses throughout multi-turn conversations, we added the following system prompt:


In multi-turn conversations, when responding to a new question from the user, you should:1. Maintain Continuity: Ensure that responses are logically connected and the conversation flows smoothly.2. Avoid Contradictions: Do not contradict previous responses.3. Ensure Consistency: Provide consistent and accurate knowledge across all interactions.


### ShennongAlpha Translate application (ShennongTranslate)

To enhance the quality of standardized translations, particularly in the field of NMMs, capturing the original text’s essence and employing standardized terminology in the target language is imperative. This way, we can ensure a coherent linguistic and terminological framework, facilitating standardized scholarly communication. However, despite the considerable advancements in Neural Machine Translation (NMT) technologies^57–59^, current NMT algorithms fall short of fulfilling our specialized requirements. To tackle this limitation, we introduced a new translation algorithm named “Neural Machine Translation Based on Coreference Primary Term (NMT-CPT)”.

At the heart of the NMT-CPT is a dual-purpose strategy. When a user submits a translation request via ShennongTranslate, the system proactively identifies standardized terms within the text recorded in ShennongKB, referencing their Primary Terms and standardized translations in the target language. Subsequently, using the specialized annotation syntax of MLMD, ShennongTranslate, powered by ShennongLLM, produces a translation that incorporates these standardized translations.

To illustrate, let’s examine the translation request mentioned in the “Results” section (Fig. 6; Supplementary Fig. S20):


Text for translation:麻黄是一种天然药材。Translation direction:zh -> enUser-customized dictionary:天然药材 -> Natural Medicinal Material


Once ShennongTranslate receives the translation request, it utilizes ShennongSearch’s CGS to identify potential NMMs mentioned within the text and search out the corresponding Primary Terms. In this case, the term “麻黄” is detected, associated with the corresponding Primary Term “nmm-0006”. Based on the Primary Term (“nmm-0006”) and the target language code (“en”), we can probe the ShennongKB’s glossary collection, ultimately obtaining its standardized translation in the “NMM Systematic Name (NMM ID, NMM Generic Name)” format:


Ephedra equisetina vel intermedia vel sinica Stem-herbaceous (NMM-0006, Ma-huang)


These standardized and user-customized translations are then formatted according to the unique MLMD syntax used in the NMT-CPT algorithm: “[[xxx | yyy]]”. After processing, the dictionary is formatted as follows:


麻黄 -> [[nmm-0006 | Ephedra equisetina vel intermedia vel sinica Stem-herbaceous (NMM-0006, Ma-huang)]]天然药材 -> [[Natural Medicinal Material]]


In the MLMD syntax, the term “麻黄” is translated as “[[nmm-0006 | Ephedra equisetina vel intermedia vel sinica Stem-herbaceous (NMM-0006, Ma-huang)]]”. Here, “nmm-0006” (i.e., the “xxx” part of the annotation) signifies the Primary Term (or NMM ID) for the term “麻黄”, as determined through a ShennongSearch’s CGS query within ShennongKB. Meanwhile, “Ephedra equisetina vel intermedia vel sinica Stem-herbaceous (NMM-0006, Ma-huang)” (i.e., the “yyy” part) represents its standardized translation in the target language. For translations in the user-customized dictionary, as they do not need to apply a ShennongSearch query to find the Primary Terms, the annotation adopts the form “[[yyy]]” without the “xxx” component.

By forwarding the user’s text to be translated, the translation direction, and the formatted dictionary to ShennongLLM, we obtain the following translation with MLMD syntax:


[[nmm-0006 | Ephedra equisetina vel intermedia vel sinica Stem-herbaceous (NMM-0006, Ma-huang)]] is a kind of [[Natural Medicinal Material]].


ShennongTranslate further parses this MLMD-styled translation to provide functionalities for term highlighting (Fig. 6 ⑥, ⑦) and tooltips (Fig. 6 ⑥’).

ShennongTranslate leverages the LangChain framework^41^ to construct translation prompt templates. This approach guarantees that translations adhere to a strict structure and consistent formatting. By employing a few-shot learning technique^60^, the system can generalize to the new translation syntax without requiring extensive model fine-tuning.

### User login and privacy

While most functionalities in ShennongAlpha are accessible without logging in, certain advanced features require users to log in (supporting login via the OAuth 2.0 protocol) to ensure optimal performance and user experience. These features include:

- Knowledge saving, contribution, and editing: users must log in to save knowledge pages (Fig. 4 ⑩) to their user profiles (Supplementary Fig. S10), or to contribute (Fig. 4 ⑭) or edit (Fig. 4 ⑮) knowledge. This allows us to properly acknowledge their contributions (Fig. 4 ⑰).
- Accessing conversation history: ShennongChat enables users to quickly access their conversation history (Fig. 5 ②), which is linked to their accounts.
- Custom glossary: ShennongTranslate permits users to add a custom glossary (Fig. 6 ③), which needs to be stored and retrieved based on user accounts.

Without user login, these personalized features cannot function effectively.

To fully ensure the security of user data and protect user privacy, we have implemented the following measures:

- Privacy first: we prioritize user privacy in all aspects of data handling.
- Data isolation: user data is stored in a separate, secure database, completely isolated from the public data in ShennongKB.
- Advanced encryption: we employ encryption technologies to ensure a high level of data security.
- Access control: within ShennongAlpha, only highly authorized personnel can access sensitive user data.
- User consent and awareness: for ShennongChat and ShennongTranslate, users’ conversation and translation records may be used to train and improve AI models. However, we provide a prominent option and notification on the user profile settings page (Supplementary Fig. S11 ⑥’), allowing users to freely choose whether to permit ShennongAlpha to use their data for improving AI models.
- Data anonymization: if users consent to allow us to use their data to improve AI models, all data will be strictly anonymized to remove any personally identifiable information.

By implementing these measures, we ensure that while users benefit from personalized features and contribute to the enhancement of our AI models, their privacy and data security remain uncompromised.

### User contribution and modification of NMM knowledge in ShennongAlpha

Upon logging into ShennongAlpha, users can modify existing knowledge entries by clicking the “Edit Content” button on NMM knowledge pages (Fig. 4 ⑮; Supplementary Fig. S12a ①) and making the desired changes (Supplementary Fig. S12b, c). After completing their edits, users submit their modifications for review by ShennongAlpha (Supplementary Fig. S12c ⑨). These submissions appear in the “Submissions” section on the user’s profile page (Supplementary Fig. S10 ①). At this stage, the modification request holds a “Review pending” status (Supplementary Fig. S12d).

New review requests are forwarded to ShennongAlpha’s internal knowledge review team, which employs a combination of manual evaluation and AI-assisted methods to assess submissions. The review focuses on:

- Factual accuracy: verifying that the modifications are relevant to the specific NMM and conform to scientific facts; supporting modifications with appropriate literature references is preferred.
- Textual accuracy: ensuring compliance with formatting guidelines (e.g., adherence to the MLMD syntax specification) and checking for typographical errors.

The review process results in two possible outcomes:

- Acceptance: for approved submissions, the user’s profile displays “Review accepted” along with the reviewer’s username (Supplementary Fig. S12e). The review records are accessible to all users to maintain transparency. The accepted modifications are integrated into ShennongKB and are immediately reflected on the corresponding NMM knowledge page (Supplementary Fig. S12g), ensuring that all platform users have access to the latest updates. The names of the contributor and reviewer, along with submission and review timestamps, are displayed, and their user avatars are featured in the “Contributors” section (Fig. 4 ⑰) to acknowledge and express gratitude for their valuable contributions. Historical versions of the content are also accessible via the “Show Edit History” button (Fig. 4 ⑯; Supplementary Fig. S12), ensuring traceability and transparency of the submission history. the "22"here is in a wrong format.
- Rejection: for rejected submissions, the reviewer’s name and specific reasons for rejection are displayed on the user’s profile page to help the user understand the rationale (Supplementary Fig. S12f). Users can revise and resubmit their modifications based on the feedback provided (Supplementary Fig. S12f’), repeating this process until the submission is accepted.

## Supplementary information

Supplementary information

Supplementary Table 1: Problems in the names of 616 NMMs in the Chinese Pharmacopoeia: 2020 Edition: Volume I.

## Data Availability

Data from ShennongKB, as well as the web and applications of the ShennongAlpha, can be accessed through the ShennongAlpha Web (https://shennongalpha.westlake.edu.cn).

## Code availability

The SNNMMA and ShennongName suite can be obtained on GitHub (https://github.com/shennong-program/shennongname). The relevant Python package has been published and released on PyPI (https://pypi.org/project/shennongname). The MLMD parser can be obtained on GitHub (https://github.com/shennong-program/mlmd). The relevant TypeScript package has been published and released on npm (https://www.npmjs.com/package/mlmd). The Python implementation of CGS can be obtained on GitHub (https://github.com/shennong-program/pycgs) or PyPI (https://pypi.org/project/pycgs). The dataset and code used for evaluating the search performance of CGS can be accessed on GitHub (https://github.com/shennong-program/pycgs). The dataset and code for evaluating (1) embedding models for vector search in NMM-related texts and (2) LLMs’ accuracy in answering NMM-related factual questions are available on GitHub (https://github.com/shennong-program/shennongllm-evals).
