# Supplementary material for: ShennongAlpha: an AI-driven sharing and collaboration platform for intelligent curation, acquisition, and translation of natural medicinal material knowledge
Source: Cell Discov. 2025 Apr 1;11:32. doi: 10.1038/s41421-025-00776-2 (PMC11961663; doi:10.1038/s41421-025-00776-2)
Supplement: Supplementary file 1 — Supplementary information [file 41421_2025_776_MOESM1_ESM.pdf]

# Supplementary Information

# A Supplementary Figures

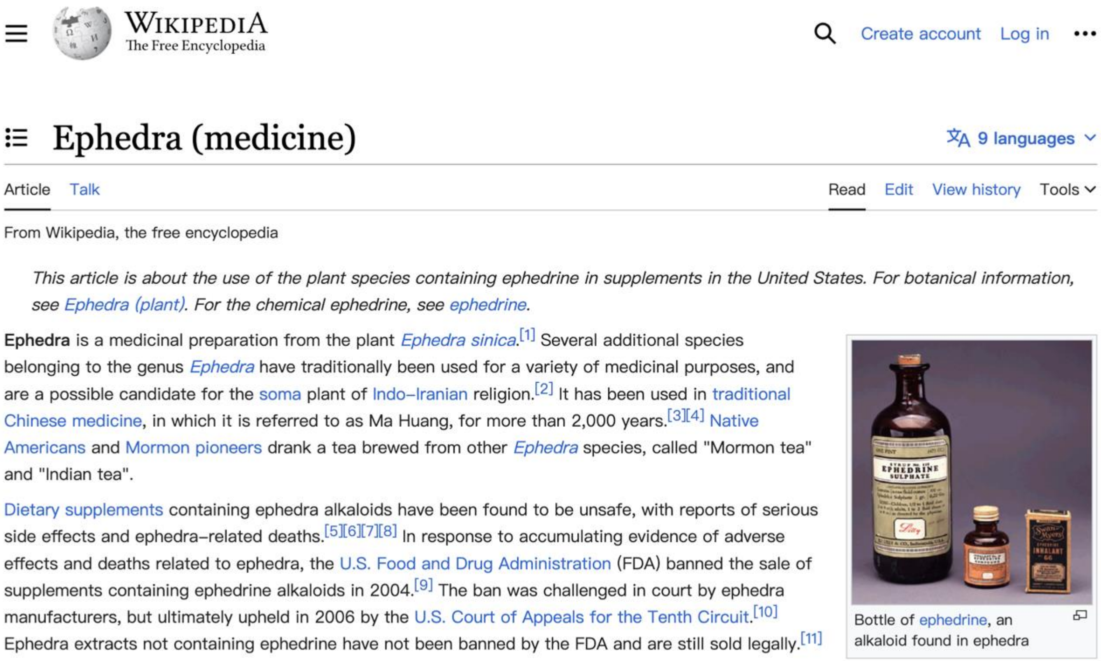

WIKIPEDIA
The Free Encyclopedia

Create account Log in

## Ephedra (medicine)

9 languages

Article Talk Read Edit View history Tools

From Wikipedia, the free encyclopedia

*This article is about the use of the plant species containing ephedrine in supplements in the United States. For botanical information, see Ephedra (plant). For the chemical ephedrine, see ephedrine.*

**Ephedra** is a medicinal preparation from the plant *Ephedra sinica*.[1] Several additional species belonging to the genus *Ephedra* have traditionally been used for a variety of medicinal purposes, and are a possible candidate for the soma plant of Indo-Iranian religion.[2] It has been used in traditional Chinese medicine, in which it is referred to as Ma Huang, for more than 2,000 years.[3][4] Native Americans and Mormon pioneers drank a tea brewed from other *Ephedra* species, called "Mormon tea" and "Indian tea".

Dietary supplements containing ephedra alkaloids have been found to be unsafe, with reports of serious side effects and ephedra-related deaths.[5][6][7][8] In response to accumulating evidence of adverse effects and deaths related to ephedra, the U.S. Food and Drug Administration (FDA) banned the sale of supplements containing ephedrine alkaloids in 2004.[9] The ban was challenged in court by ephedra manufacturers, but ultimately upheld in 2006 by the U.S. Court of Appeals for the Tenth Circuit.[10] Ephedra extracts not containing ephedrine have not been banned by the FDA and are still sold legally.[11]

Bottle of ephedrine, an alkaloid found in ephedra

**Supplementary Fig. S1: Wikipedia entry: Ephedra.**

Wikipedia states that the species origin of Ephedra is *Ephedra sinica* and claims that it is known as “Ma Huang” in traditional Chinese medicine. However, this information is inaccurate. According to China’s most authoritative pharmaceutical reference, *Chinese Pharmacopoeia (2020 Edition)*, Ephedra is a multi-species NMM. Its species origins include *Ephedra sinica*, *Ephedra intermedia*, or *Ephedra equisetina*.

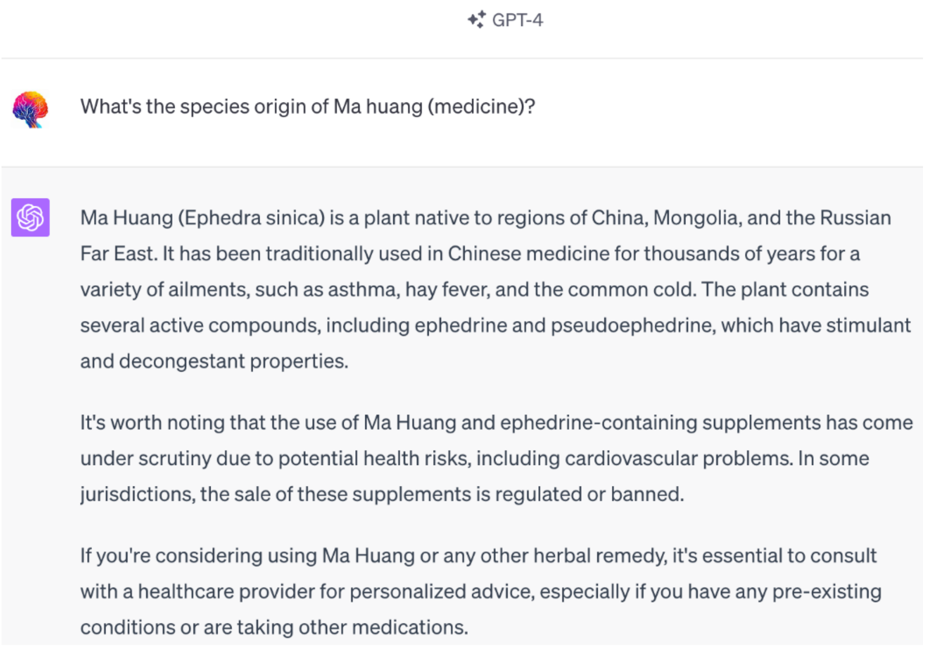


**Supplementary Fig. S2: GPT-4's answer on the species origin of Ma Huang.**

GPT-4 incorrectly asserts that Ma Huang is a single-species NMM based solely on *Ephedra sinica*.

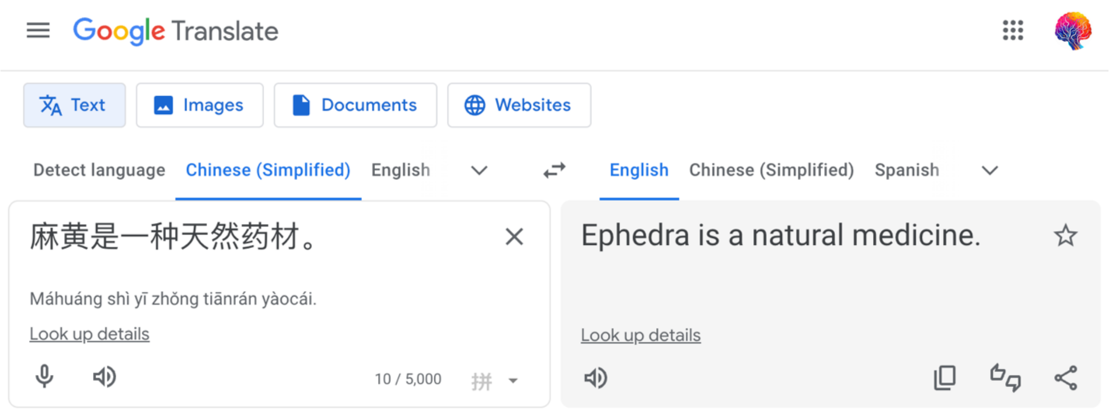


**Supplementary Fig. S3: Google Translate's translation of text related to "Ma Huang" (麻黄).**

Due to the lack of a systematic nomenclature for NMMs, Google Translate simplifies the translation of "麻黄" to "Ephedra", failing to reflect the multi-species intricacies of "麻黄". This mistranslation could lead English-speaking users to incorrectly use the term "Ephedra" for further retrieval of knowledge, potentially directing them to the aforementioned Wikipedia entry containing inaccurate information, thereby hindering the globalization of NMM knowledge.

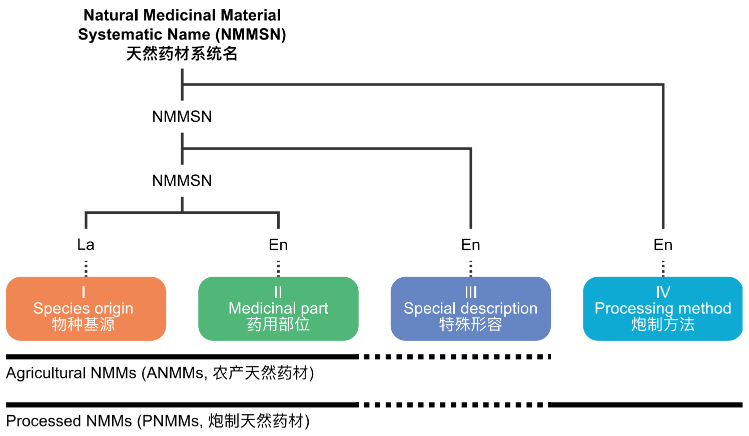


**Supplementary Fig. S4: Parsing structure of the NMM Systematic Name.**

The parsing structure of a valid NMM Systematic Name (NMMSN) involves a lawful combination of four naming components. At a minimum, an NMMSN includes components I and II. For Agricultural NMMs, an NMMSN may include components I, II, and III; for Processed NMMs, it may include components I, II, III, and IV. A solid line indicates that these components are required for that type of NMM; a dashed line indicates that component III is optional.

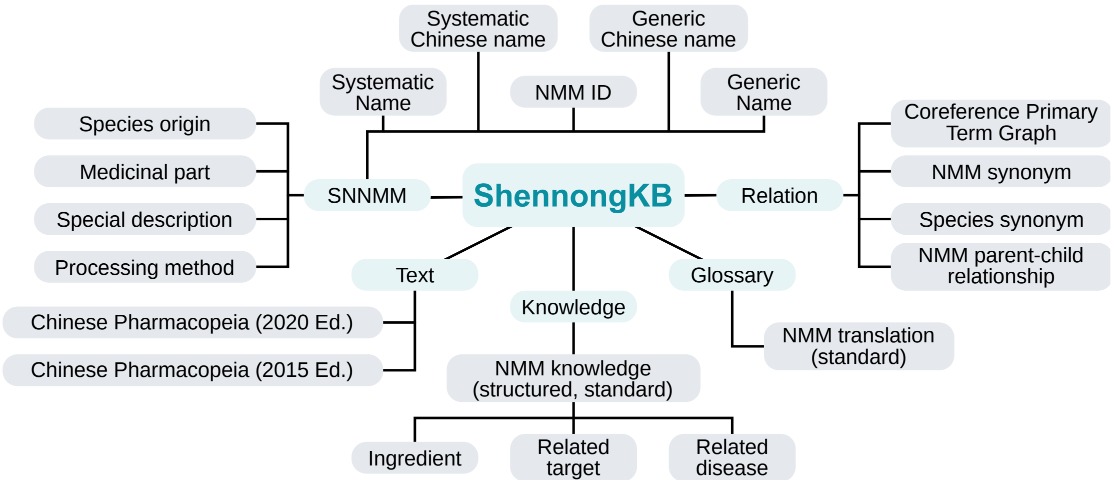


**Supplementary Fig. S5: NMM knowledge curated in the ShennongAlpha Knowledge Base.**

The ShennongAlpha Knowledge Base (ShennongKB) encompasses five major categories of information, including the Systematic Nomenclature for Natural Medicinal Materials (SNNMM), text, knowledge, glossary, and relation.

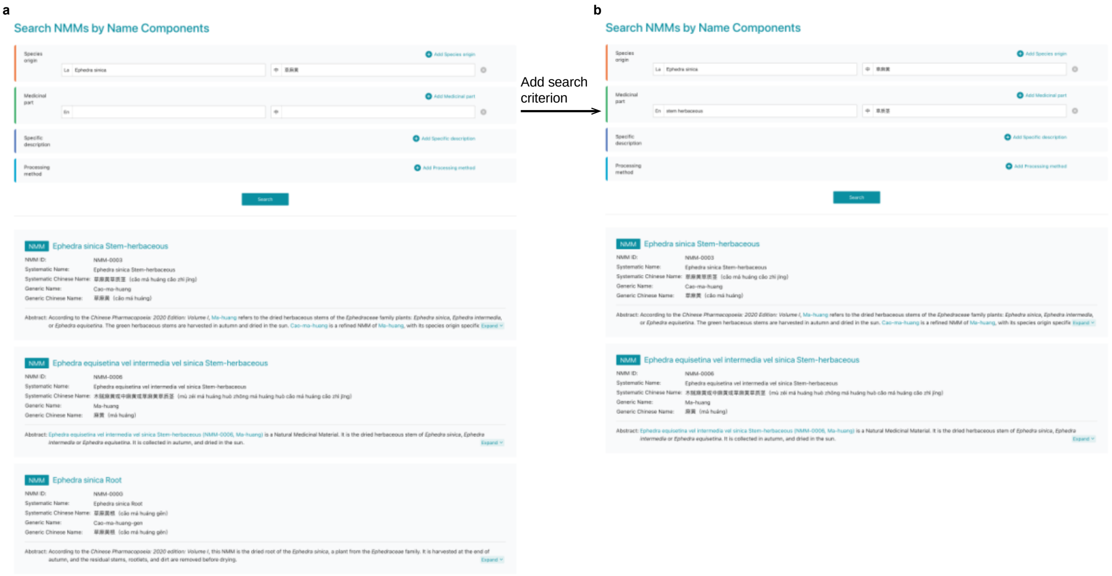


**Supplementary Fig. S6: Advanced search mode “Search by NMM Components”.**

**a.** Search results when the search criterion is species origin: “Ephedra sinica”. The search retrieves NMMs such as “Ephedra sinica Stem-herbaceous”, “Ephedra equisetina vel intermedia vel sinica Stem-herbaceous”, and “Ephedra sinica Root” because their species origin is *Ephedra sinica*. **b.** Search results when adding the search criterion medicinal part: “stem herbaceous” on top of **a**. In this case, “Ephedra sinica Root” is no longer included in the search results because its medicinal part is “root”, which does not meet the search criterion “stem herbaceous”.

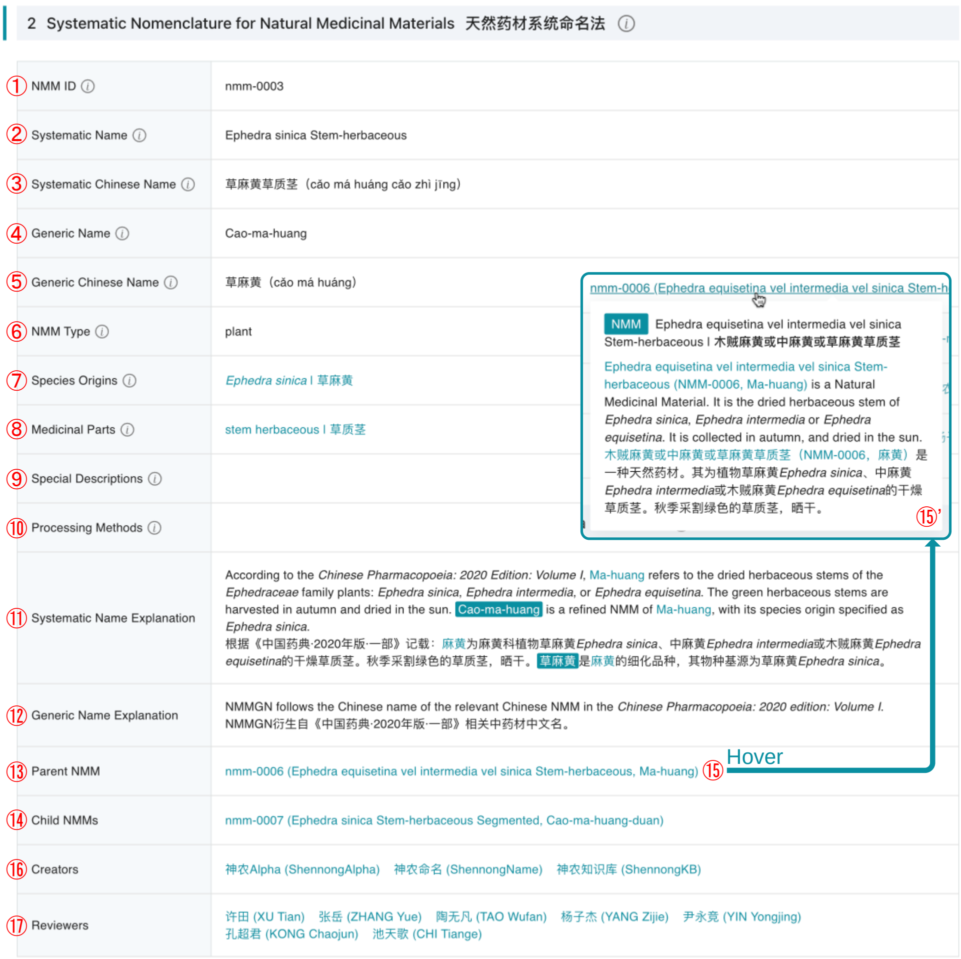


**Supplementary Fig. S7: “Systematic Nomenclature for Natural Medicinal Materials” section of the ShennongAlpha knowledge page.**

This section displays each NMM along with its identifiers as defined by the Systematic Nomenclature for Natural Medicinal Materials (SNNMM), including the NMM ID (①), NMM Systematic Name (②), NMM Systematic Chinese Name (③), NMM Generic Name (④), and NMM Generic Chinese Name (⑤). It specifies the NMM’s type (⑥) within the SNNMM framework and provides detailed key information corresponding to its four naming components (⑦-⑩), accompanied by explanations for the formation of both the Systematic Name (⑪) and the Generic Name (⑫). Additionally, information about the parent (⑬) and child (⑭) NMMs is displayed; each NMM has at most one parent NMM but may have multiple child NMMs. When users hover over an NMM ID of the parent or child NMMs (⑮), a tooltip appears (⑮’), providing a summary of the knowledge related to that NMM. The section also showcases the creators (⑯) and reviewers (⑰) of the NMM’s SNNMM information, helping users assess the source and accuracy of the information while acknowledging and recognizing the contributions of these individuals.

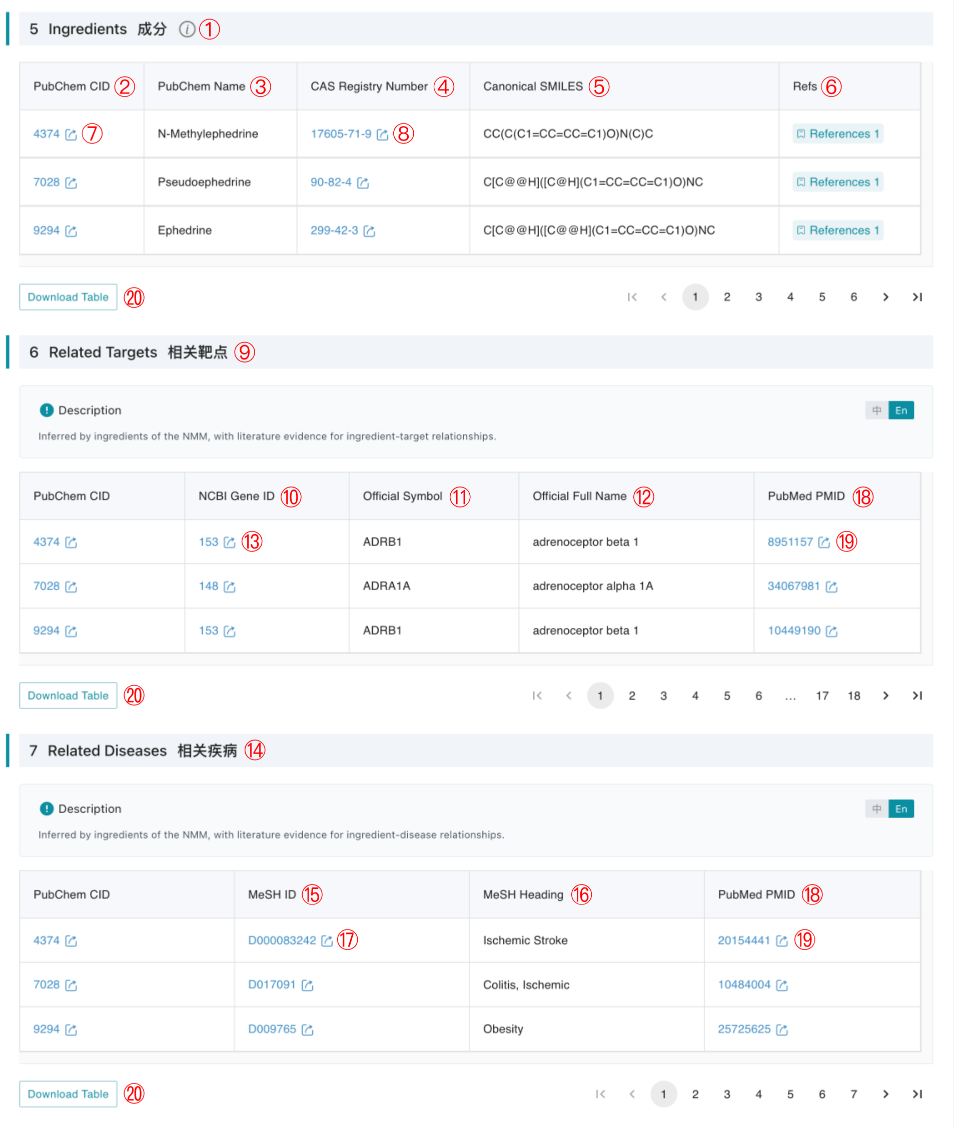


**Supplementary Fig. S8: The "Ingredients", "Related Targets", and "Related Diseases" sections of the ShennongAlpha knowledge page.**

In the "Ingredients" section (①), each NMM ingredient is standardized based on the PubChem database. For each ingredient, standardized information is provided, including the PubChem CID (②), PubChem Name (③), CAS Registry Number (④), and Canonical SMILES (⑤), along with references to data sources (⑥). Clicking on the hyperlinks for PubChem CID (⑦) and CAS Registry Number (⑧) redirects users to the corresponding pages in the PubChem and CAS databases. In the "Related Targets" section (⑨), each NMM-related gene target is standardized using the NCBI Gene database. Each gene entry includes the standardized NCBI Gene ID (⑩), Official Gene Symbol (⑪), and Official Gene Full Name (⑫). Clicking on the NCBI Gene ID hyperlink (⑬) takes users to the corresponding NCBI Gene database page. In the "Related Diseases" section (⑭), each NMM-related disease is standardized according to the MeSH database, providing the MeSH ID (⑮) and MeSH Heading (⑯) for each

disease. Clicking on the MeSH ID hyperlink (⑰) directs users to the corresponding page in the MeSH database. For both related targets and diseases, corresponding literature evidence is provided, including PubMed PMIDs (⑱). Clicking on the PubMed PMID hyperlinks (⑲) leads to the relevant entries in the PubMed database. Each section offers a download button (⑳), allowing users to download the tabulated data in CSV format for further analysis.

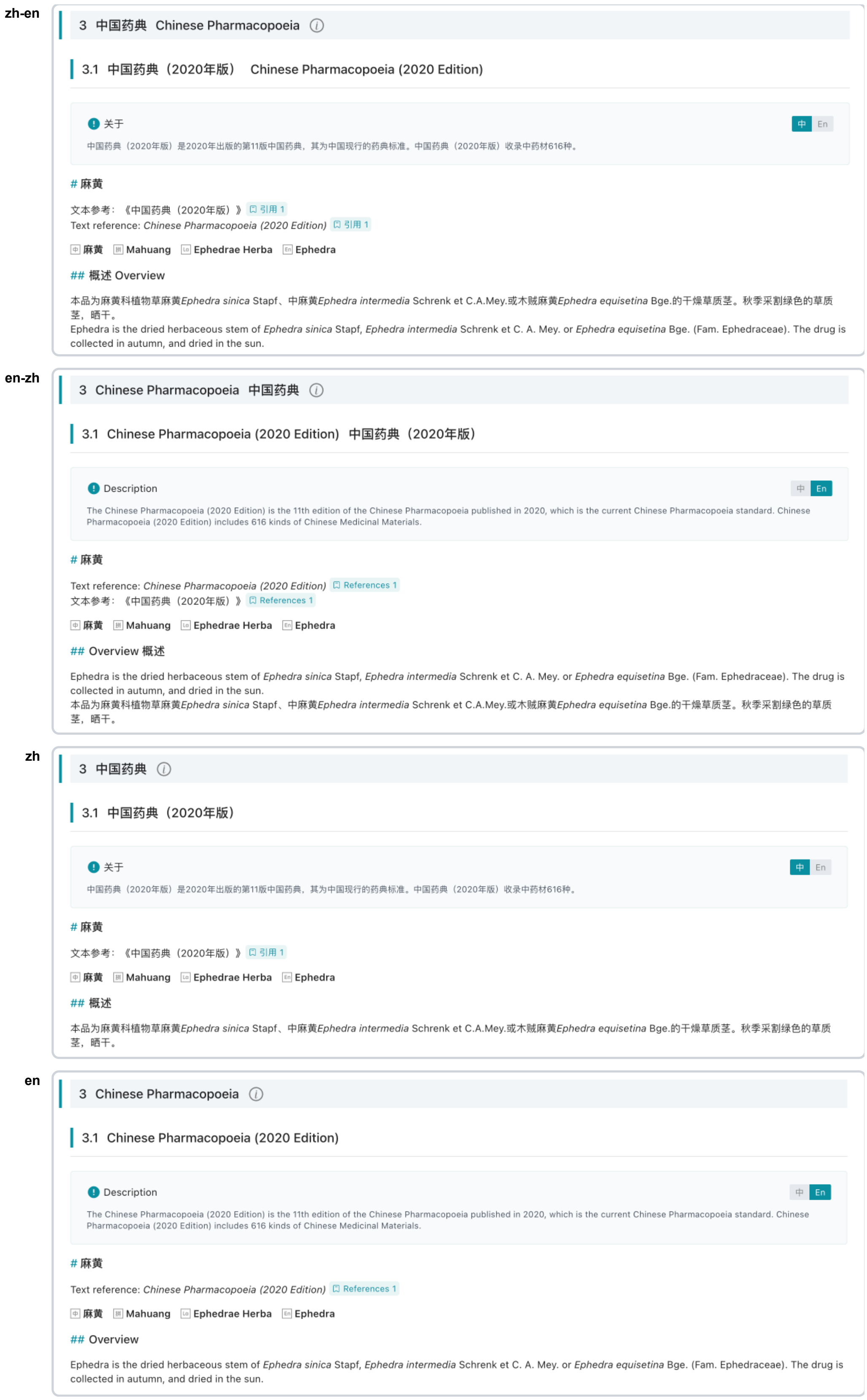


**Supplementary Fig. S9: Language display modes in the ShennongAlpha Web.**

The ShennongAlpha Web adopts a bilingual design in both Chinese and English, utilizing MLMD to curate parallel Chinese and English texts within a single document. ShennongAlpha supports four language display modes: zh-en, en-zh, zh, and en. Notably, in the zh-en and en-zh modes, both the section titles and text of the knowledge are displayed bilingually, differing only in their display order (in the zh-en mode, Chinese precedes English, whereas in en-zh, English comes before Chinese). In the zh and en modes, both the section titles and text of the knowledge are displayed solely in Chinese or English, respectively.

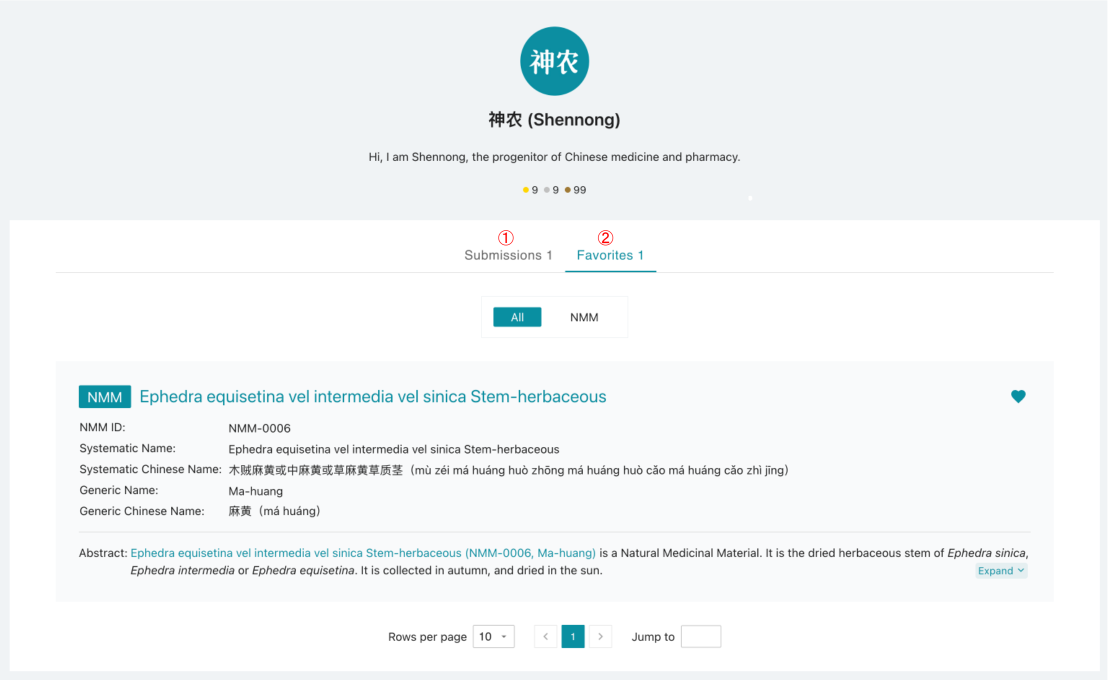


**Supplementary Fig. S10: User profile page of ShennongAlpha.**

On this page, users can view a user's (either themselves or another user) username, self-introduction, and contribution points; as well as all of the user's submissions related to knowledge additions and modifications in ShennongAlpha (①); or the user's Favorites (②).

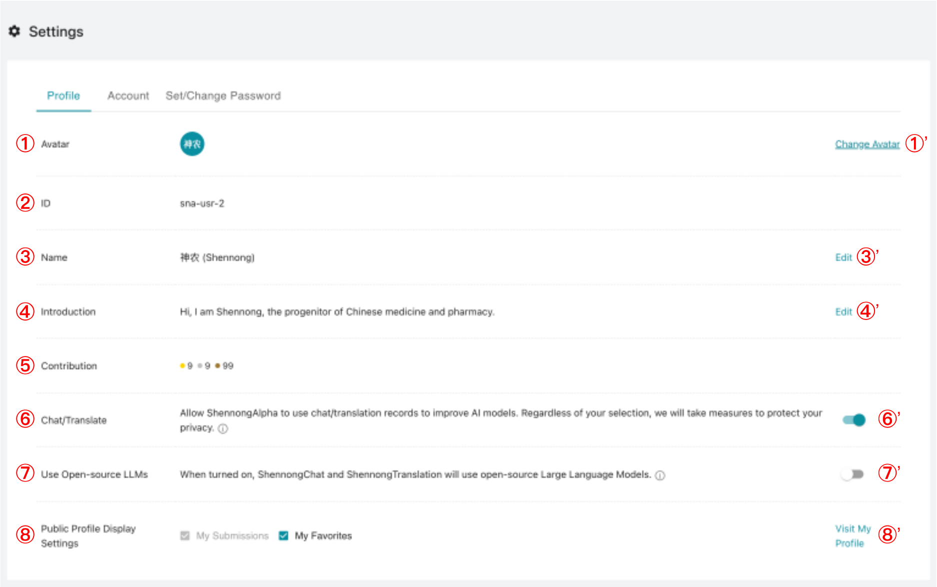


**Supplementary Fig. S11: User settings page of ShennongAlpha.**

On this page, users can view their avatar (①), unique user ID (②), username (③), self-introduction (④), contribution points (accumulated when users add, edit, or review knowledge) (⑤), settings for the Chat/Translate applications (⑥, ⑦), and settings for the content sections displayed on their user profile page (⑧). Users can freely change their avatar (①'), username (③'), or self-introduction (④'). They can also toggle the setting to enable or disable the use of their Chat/Translate data for potentially improving AI models (⑥'), or whether to use open-source LLMs (⑦'). Clicking the hyperlink at (⑧') redirects to the user's profile page (**Supplementary Fig. S10**).

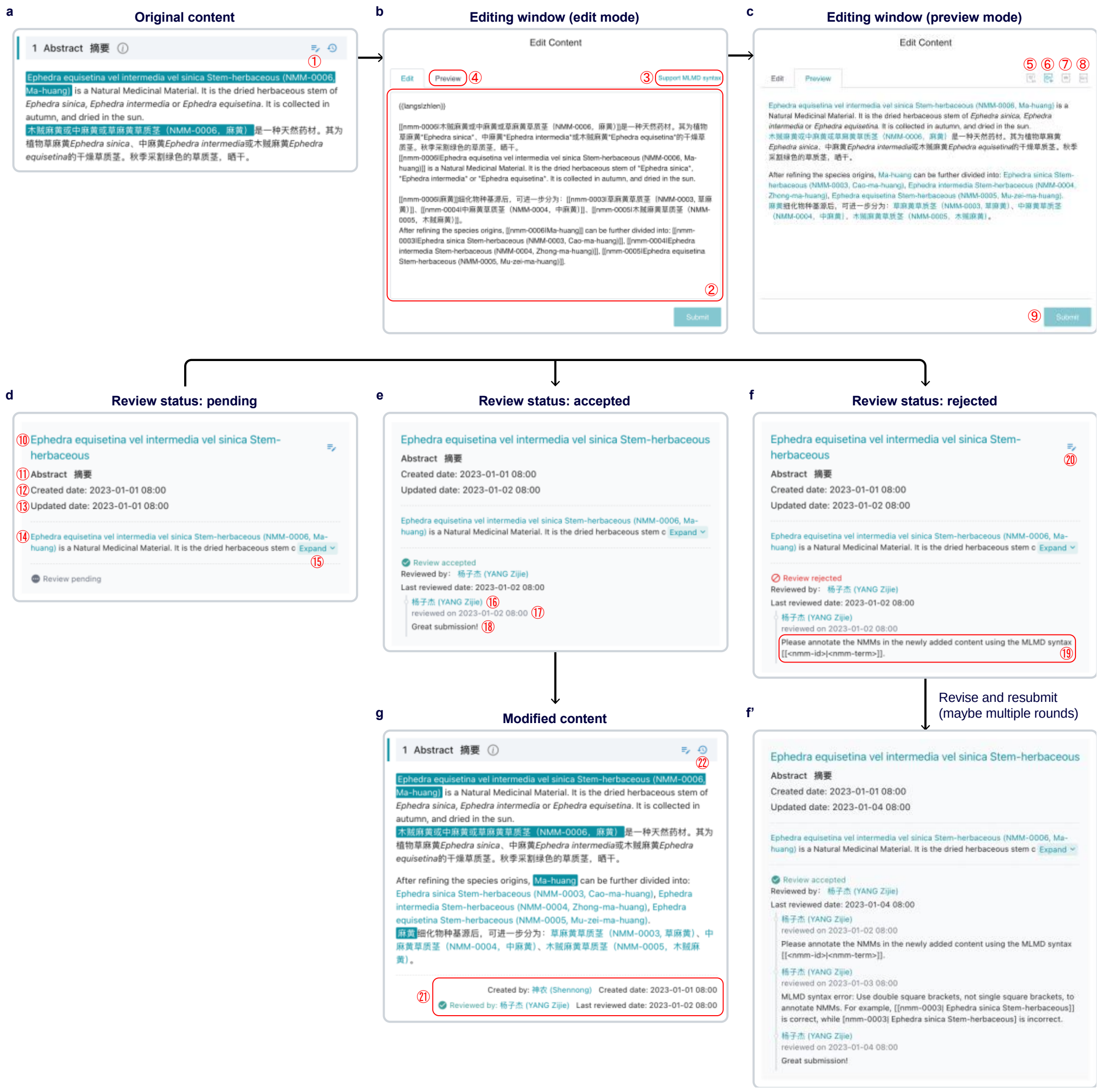


**Supplementary Fig. S12: ShennongAlpha knowledge modification and review mechanism.**

**a**. A content segment pending modification from the "Abstract" section (**Fig. 4** ⑦) of the knowledge page for "Ephedra equisetina vel intermedia vel sinica Stem-herbaceous" (NMM-0006). When the user clicks the "Edit Content" button (①), a content editing pop-up window appears (**b**). In the editing pop-up window, users can modify or add content in the editing box (②). The content in ShennongAlpha is managed in MLMD format; users can click ③ to view detailed MLMD syntax guidelines. After editing, users can click the "Preview" button (④), switching the editing pop-up to preview mode (**c**). Users can click buttons (⑤-⑧) to switch the display modes of the content among "zh-en", "en-zh", "zh", and "en", respectively. After confirming the content is correct, users can click the "Submit" button (⑨) to proceed. The user's new submission appears in the "Submissions" section on their profile page (**Supplementary Fig. S10** ①) with the status "Review pending" (**d**). The submission displays the modified NMM entry (⑩), the corresponding section (⑪), the submission creation time (⑫), the

update time (⑬), and the submitted content (⑭). Users can click the "Expand" button (⑮) to view the full submission content. The new submission is sent to ShennongAlpha's internal review team for evaluation. The review results can have two outcomes: accepted (**e**) or rejected (**f**). For accepted submissions (**e**), the reviewer's name (⑯), review time (⑰), and review comments (⑱) are displayed to ensure transparency. For rejected submissions (**f**), the reviewer provides the reasons for rejection (⑲) to help the user understand the cause. Based on the review comments, users can click ⑳ to revise the previous submission and resubmit, repeating this process until the submission is accepted (**f'**; multiple rounds are allowed). Accepted submissions are immediately updated in the corresponding section of the relevant NMM (**g**), allowing all users to access the latest content. The names of the submitter and reviewer are displayed (㉑), and their avatars appear in the "Contributors" section (**Fig. 4 ⑰**) to acknowledge their valuable contributions. Users can click the "Show Edit History" button (㉒) to view historical versions of the content, ensuring traceability and transparency of edits.

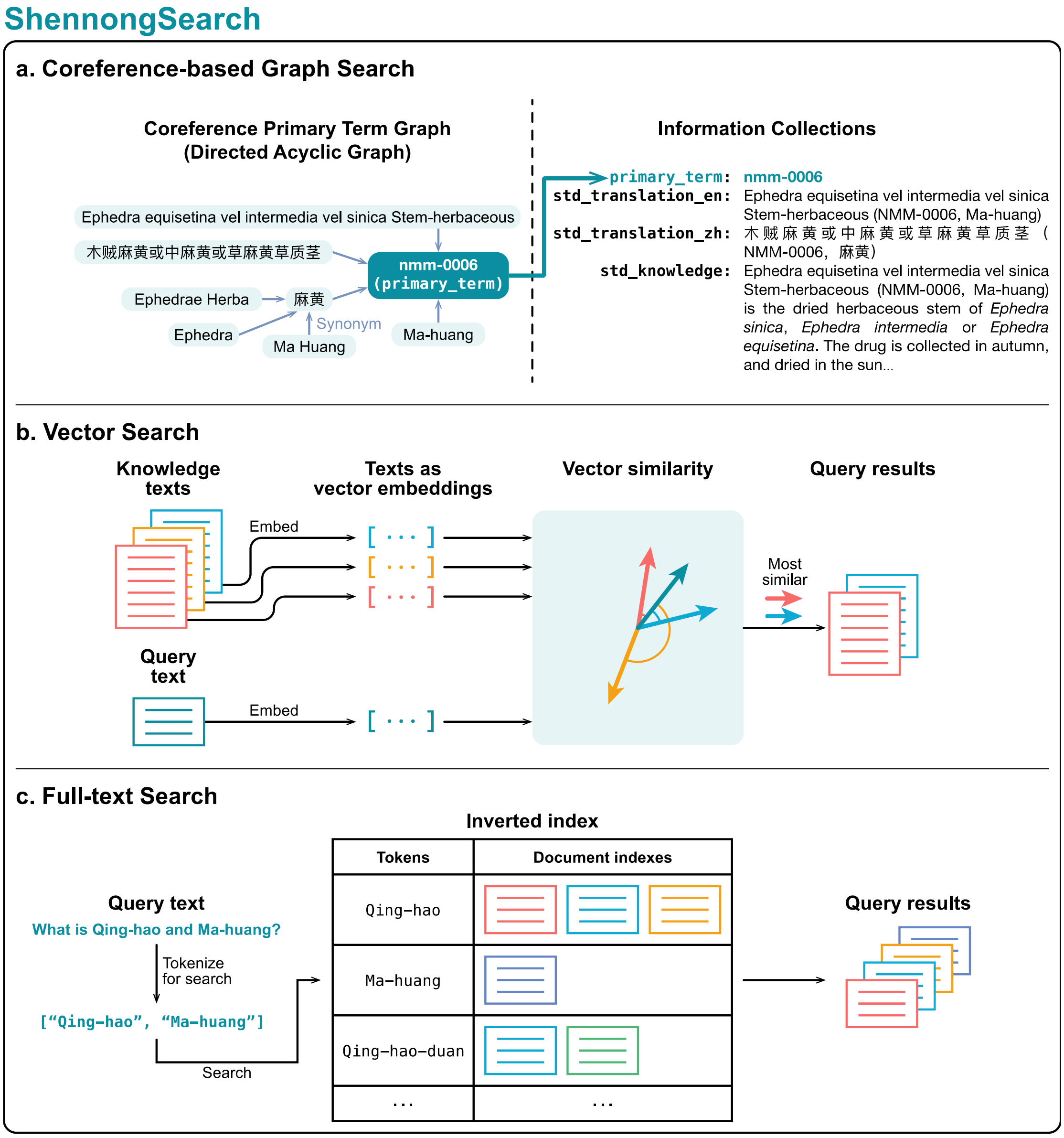


**Supplementary Fig. S13: Advanced search methods in the ShennongAlpha Search Engine.**

The ShennongAlpha Search Engine (ShennongSearch) has been designed to offer three distinct advanced search methods to cater to the specific search needs associated with NMMs: **a**. CGS, utilizing a Coreference Primary Term Graph (CPTG) to depict the relationships among NMMs' synonyms, thereby facilitating the retrieval of standardized knowledge and translations for a given NMM name; **b**. Vector search, which evaluates the similarity between the vector embeddings of queries and the texts archived in ShennongKB, allowing for semantic matches; **c**. Full-text search, employing tokenization and an inverted index to pinpoint and yield the pertinent fuzzy results.

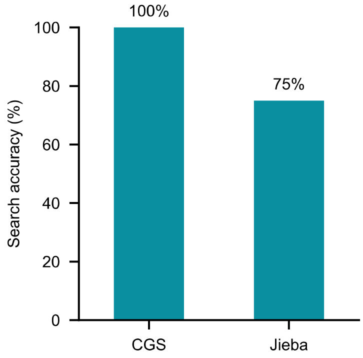


**Supplementary Fig. S14: Accurate extraction of NMM terms from text using CGS.**

We tested the accuracy of detecting NMM terms in 40,000 texts containing NMM terms (e.g., “Artemisia annua Part-aerial is a kind of Natural Medicinal Material.”), which included both Chinese and English texts with various NMM terms in both languages. Two methods were used: the CGS Primary Term Extractor algorithm (**Supplementary Method H, Algorithm 3**, represented by the “CGS” bar) and the Jieba-based segmentation method (represented by the “Jieba” bar). In the given example, the NMM term is “Artemisia annua Part-aerial”. The CGS method accurately identified all NMM terms present in the texts, achieving a 100% detection rate. In contrast, the Jieba-based method achieved a detection rate of only 75%.

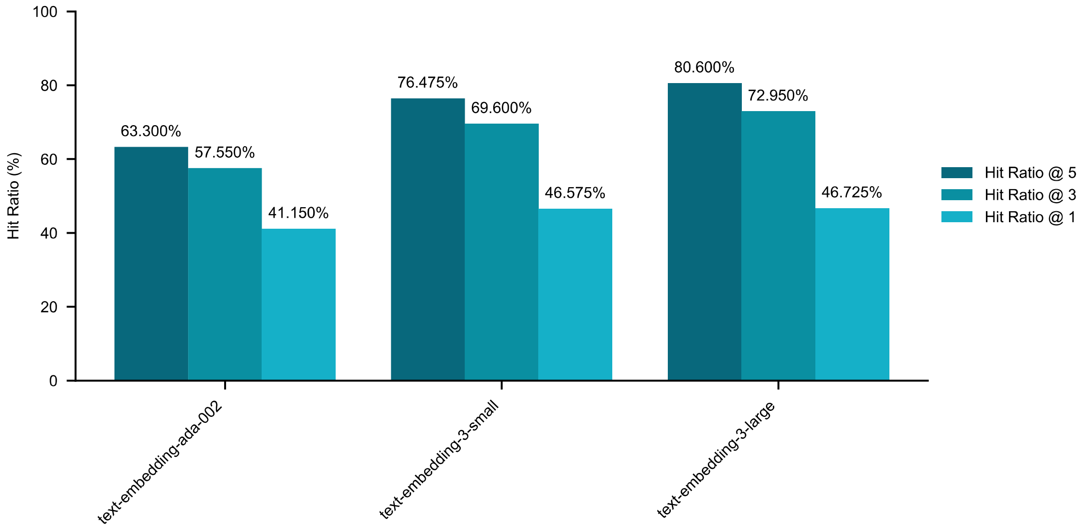


**Supplementary Fig. S15: Performance of text embedding models for encoding NMM-related texts and vector search.**

We tested the performance of three mainstream text embedding models (text-embedding-ada-002, text-embedding-3-small, and text-embedding-3-large) used to encode factual NMM-related texts into vector representations for subsequent vector search. These models each encode a text into vectors of dimensions 1536, 1536, and 3072, respectively. This implies that the text-embedding-3-large model requires twice as much storage space compared to the other two models and incurs additional computational cost for each vector similarity calculation. We constructed 4,000 queries related to 1,000 different NMMs (e.g., "What is the Natural Medicinal Material ID (NMM ID) of Ephedra equisetina vel intermedia vel sinica Stem-herbaceous?"), and for each of these 1,000 NMMs, we generated one factual text (e.g., "NMM ID: nmm-0006. NMM Systematic Name: Ephedra equisetina vel intermedia vel sinica Stem-herbaceous. NMM Systematic Chinese Name: 木贼麻黄或中麻黄或草麻黄草质茎. NMM Generic Name: Ma-huang. NMM Generic Chinese Name: 麻黄."). Therefore, for each query, there is only one relevant text among the 1,000 NMM texts that answers the query. The goal of the vector search evaluation is to determine whether the correct NMM text can be retrieved from the 1,000 texts for each query. During the testing of different models, embeddings for each query and all NMM texts were generated using the same model, and cosine similarity was computed between each query and every text. The texts were then ranked by similarity. We evaluated the search accuracy and performance by calculating the Hit Ratio @ N at three values of N (5, 3, 1) for the search results of the 4,000 queries. The Hit Ratio @ N measures the percentage of queries for which the top N most similar texts contain the corresponding target NMM text. For example, if 3,000 of 4,000 queries retrieve the target text among the top 5 search results, the model's Hit Ratio @ 5 is 75%.

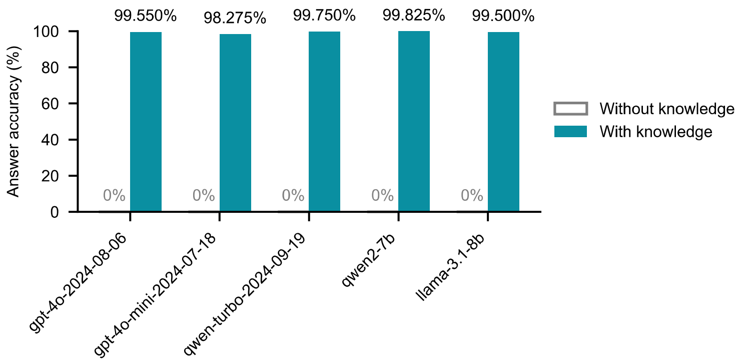


**Supplementary Fig. S16: Accuracy of different LLMs in answering NMM-related factual questions with and without background knowledge.**

We evaluated the accuracy of various proprietary models (gpt-4o-2024-08-06, gpt-4o-mini-2024-07-18, qwen-turbo-2024-09-19) and open-source models (qwen2-7b, llama-3.1-8b) in answering 4,000 NMM-related factual questions. An example of such a

question is: “What is the Natural Medicinal Material ID (NMM ID) of Ma-huang?” These NMM factual knowledge pieces are introduced for the first time in our research and do not exist in any publicly available training datasets. Therefore, none of these models could have learned the relevant knowledge during training and thus cannot correctly answer these questions. For example, gpt-4o-2024-08-06 generates a hallucinated answer: “The Natural Medicinal Material ID (NMM ID) of Ma-huang is NMM0001722.” However, when we injected the relevant background knowledge into the models’ context and then asked them to answer, their factual accuracy significantly improved. For example, we provided the information that the NMM ID of Ma-huang is “NMM-0006”. With this background, both proprietary models and smaller-parameter open-source models achieved factual accuracies close to 100%.

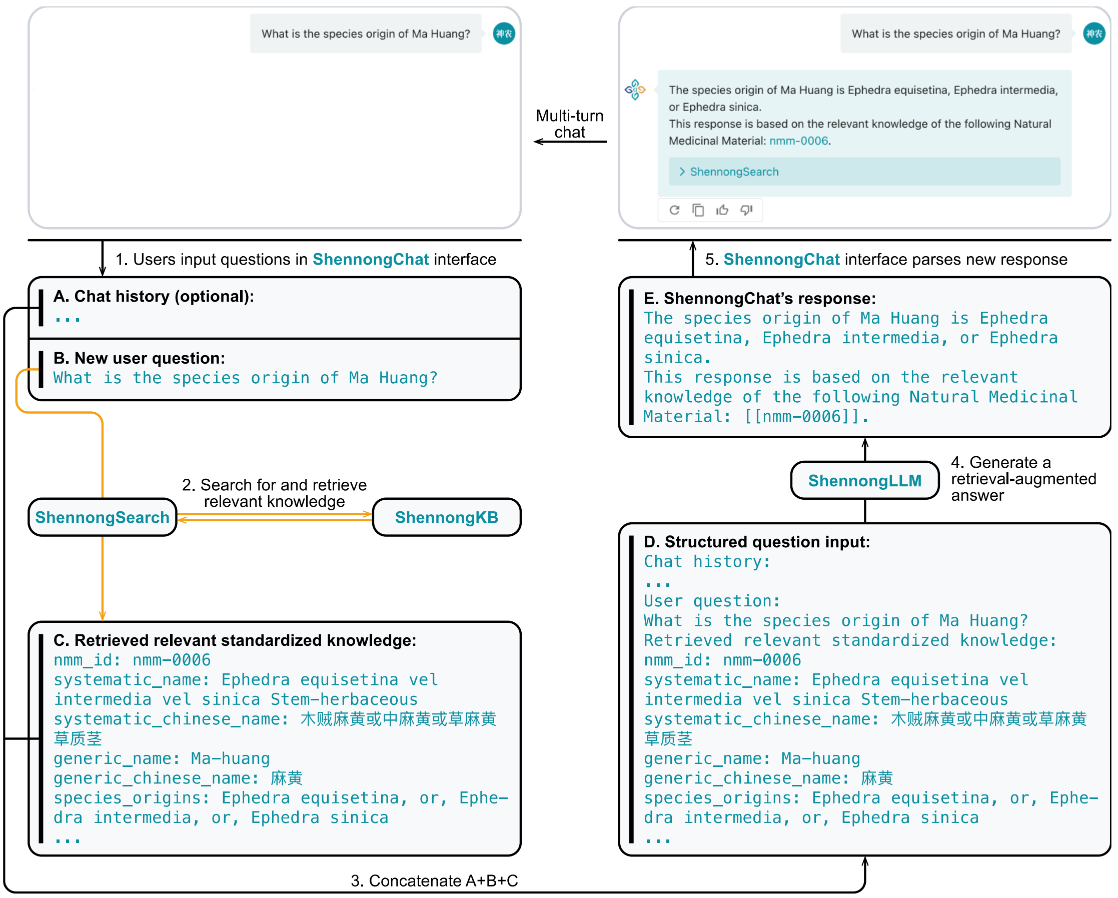


**Supplementary Fig. S17: Algorithm behind ShennongChat.**

Upon receiving a new question from a user, ShennongChat proactively uses ShennongSearch to query ShennongKB to find relevant standardized knowledge. Specifically, knowledge related to the species origin of “Ma Huang” is retrieved. ShennongChat then integrates the user’s previous chat history, the new question, and the retrieved standardized knowledge to generate a retrieval-augmented answer. In the

response, ShennongChat also indicates the NMM IDs of the relevant NMM knowledge referenced. This answer is subsequently displayed on the ShennongChat user interface. The interactive interface parses the special MLMD syntax "[[…]]" included in the response to provide highlighting of the NMM IDs (**Fig. 5** ⑨) and the functionality of displaying a knowledge summary tooltip when hovering over them (**Fig. 5** ⑨').

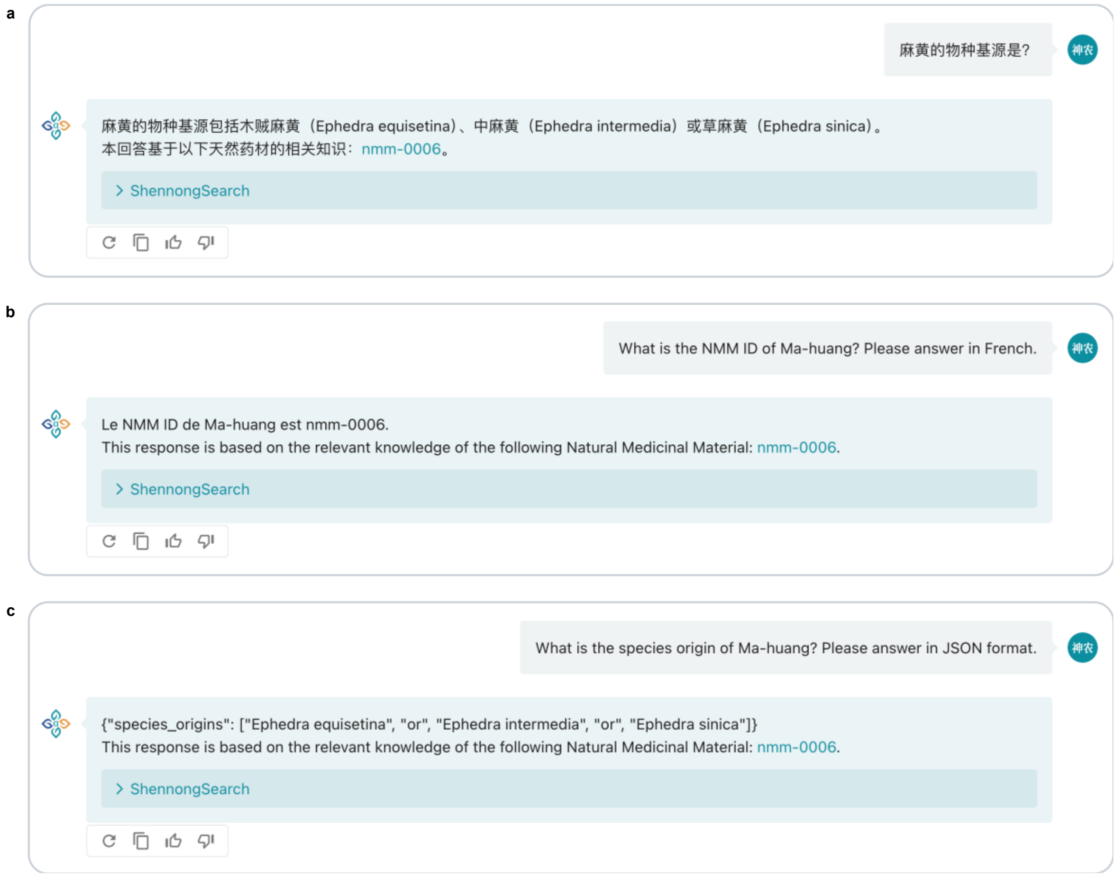


**Supplementary Fig. S18: ShennongChat offers personalized questioning and answering styles for users.**

Users can use their preferred language (such as Chinese) to question ShennongChat and receive answers in the same language (**a**). Users can also specify their desired answer style by describing it in natural language in their question, such as opting for a particular language (**b**) or a specific format (**c**). ShennongChat will tailor its answers to users' indicated styles.

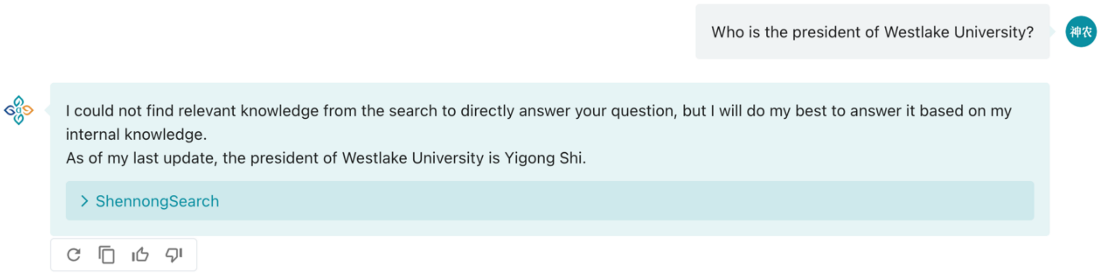


**Supplementary Fig. S19: ShennongChat attempts to answer using internal knowledge when no relevant knowledge is found.**

In the current version, ShennongKB does not contain information about the president of Westlake University. Therefore, ShennongSearch cannot retrieve any corresponding relevant knowledge. In such cases, ShennongChat will attempt to answer the user's question using the internal knowledge of the LLM employed within ShennongChat. It is important to note that when ShennongChat provides an answer without support from relevant knowledge in ShennongKB, it will state that no relevant knowledge was found and that it is using internal knowledge to answer. This approach allows ShennongChat not only to answer questions based on the knowledge included in ShennongKB but also to utilize its internal LLM's knowledge to respond to potentially broad user inquiries—even when the answers are not directly supported by ShennongKB. This increases the flexibility and adaptability of ShennongChat's responses, providing users with a better experience while also ensuring the academic rigor of the response.

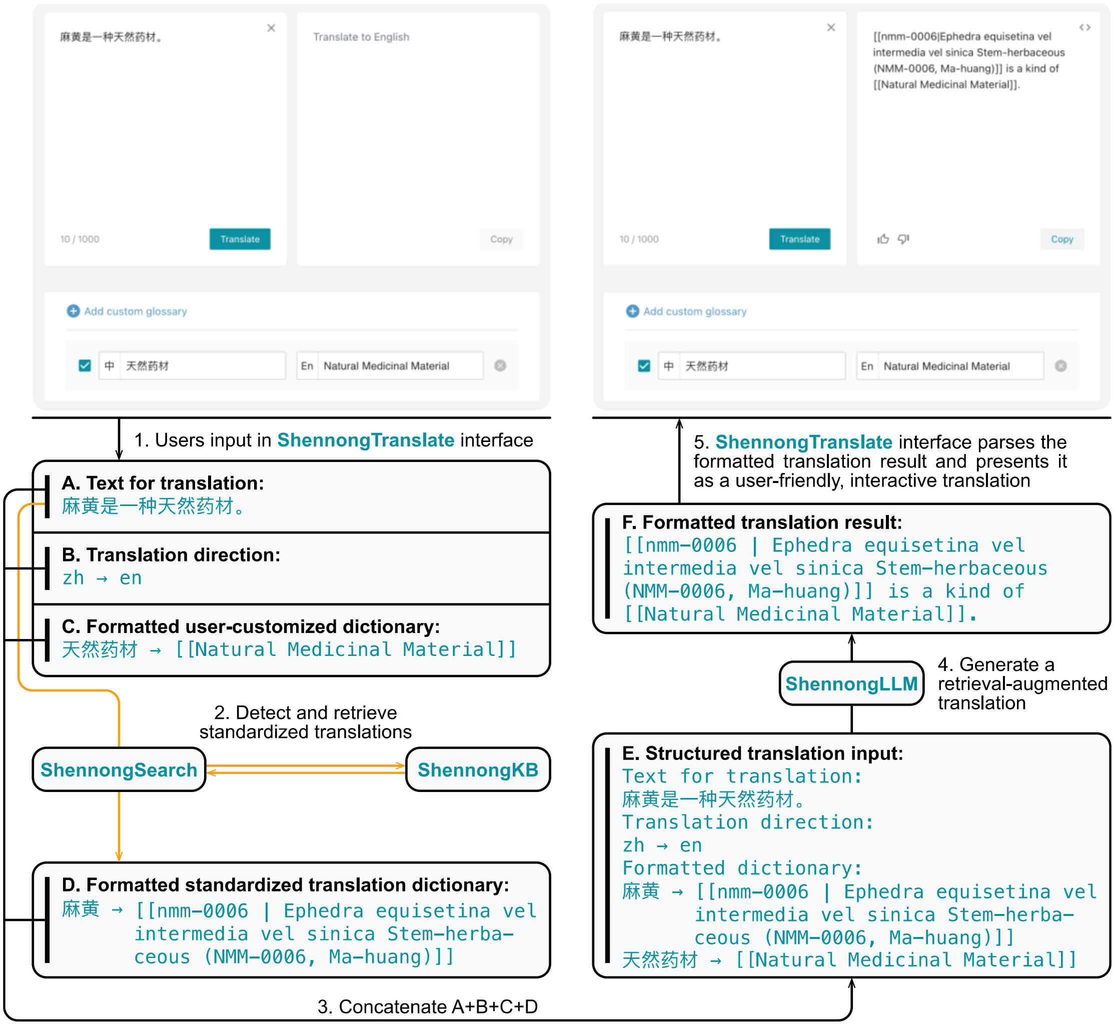


**Supplementary Fig. S20: Algorithm behind ShennongTranslate.**

ShennongTranslate employs the translation algorithm we proposed, named "Neural Machine Translation based on Coreference Primary Term (NMT-CPT)". Following a sequence from steps 1-5, ShennongTranslate generates a standardized translation with the format shown in F. The annotation "[[xxx | yyy]]" is a proprietary MLMD syntax used in the NMT-CPT. Here, "xxx" stands for the Primary Term retrieved from ShennongKB, while "yyy" denotes its standardized translation in the target language.

## B Supplementary Tables

**Supplementary Table S1: Problems in the names of 616 NMMs in the *Chinese Pharmacopoeia: 2020 Edition: Volume I.***

Please refer to the attached Excel spreadsheet for specific table contents.

**Supplementary Table S2: ShennongAlpha Knowledge Base data counts.**

Data as of 2024-10-01. The up-to-date data counts are available on the ShennongAlpha Statistics page (https://shennongalpha.westlake.edu.cn/statistics).

| Type | Count | Description |
|---|---|---|
| NMM | 14,256 | Unique NMMs under the Systematic Nomenclature, with distinct NMM IDs, Systematic Names, and Generic Names |
| NMM knowledge | 14,256 | Structured, standardized knowledge of NMMs |
| NMM standardized translation | 14,256 | Standardized bilingual (Chinese-English) translations of NMMs |
| NMM text in ChP-2020 | 616 | NMM monographs and their text in the *Chinese Pharmacopoeia (2020 edition)* |
| NMM text in ChP-2015 | 618 | NMM monographs and their text in the *Chinese Pharmacopoeia (2015 edition)* |
| NMM synonym | 58,872 | Synonymous names of NMMs |
| Species origin | 7,125 | Relevant species origins of the curated NMMs |
| Medicinal part | 347 | Relevant medicinal parts of the curated NMMs |
| Processing method | 78 | Relevant processing methods of the curated NMMs |
| Ingredient | 21507 | Ingredients collected and standardized via the PubChem database |
| Target | 12933 | Targets collected and standardized via the NCBI Gene database |
| Disease | 3198 | Diseases collected and standardized via the MeSH database |

## Supplementary Text C: Systematic Nomenclature for Natural Medicinal Materials (SNNMM)

**Rule 1:** Two names one ID rule. Each Natural Medicinal Material (NMM) is assigned an NMM Systematic Name (NMMSN), an NMM Generic Name (NMMGN), and an NMM ID.

**Rule 2:** Uniqueness rule. The NMMSN, NMMGN, and NMM ID generated under SNNMM are all unique.

**Rule 3:** ANMM/PNMM distinct name rule. SNNMM classifies NMMs into 3 types: Raw NMMs (RNMMs), Agricultural NMMs (ANMMs), and Processed NMMs (PNMMs) (**Fig. 2b**). RNMMs are generally regulated as agricultural products, while PNMMs are generally regulated as drugs.

**Rule 4:** Legitimate character rule. To allow people from any country to easily input NMMSN or NMMGN using their standard keyboard, naming NMMSN and NMMGN can only use the following characters: a-zA-Z, and the hyphen "-".

**Rule 5:** Case-insensitive rule. In NMMSN or NMMGN, even though both uppercase and lowercase letters might be used, it's only for ease of reading. Different NMMs cannot be distinguished by different cases in the SNNMM.

**Rule 6:** Upright type rule. NMMSN or NMMGN should be written in upright type.

**Rule 7:** NMMSN rules.

**Rule 7.1:** Standard Chinese NMMSN correspondence rule. To facilitate communication in a Chinese context, for each NMM named with NMMSN, SNNMM will also provide a corresponding standard Chinese systematic name, i.e., NMMSN-zh.

**Rule 7.2:** NMMSN composition Rule. NMMSN consists of the following 4 name components (**Fig. 2a**): I. Species origin; II. Medicinal part; III. Special description; IV. Processing method.

**Rule 7.3:** NMMSN syntax parsing rule. Based on the principles of natural language processing and computational linguistics, the syntax parsing structure of NMMSN is as shown in **Supplementary Fig. S4**.

**Rule 7.4:** Minimal NMMSN composition rule. The NMMSN of ANMM must contain at least the first two name components: I and II; The NMMSN

of PNMM must contain at least three name components: I, II, and IV (**Supplementary Fig. S4**).

**Rule 7.5:** NMMSN order rule. The sequence of name components in an NMMSN must adhere to a specific order: I-II-III-IV. In the case of NMMSN-zh, which follows Chinese conventions, the components are ordered as IV-III-I-II.

**Rule 7.6:** Species origin naming rules.

**Rule 7.6.1:** Complete taxonomic name rule. Prioritize the use of the full Latin taxonomic name of a species when naming. When a specific species can be identified, do not just use the genus name or species epithet for naming.

**Ex.** ✓ NMMSN: Solidago decurrens Herb[61]; NMMSN-zh: 一枝黄花全草 (species origin: *Solidago decurrens*)

✗ Solidago Herb

**Ex.** ✓ NMMSN: Panax ginseng Rhizome and Root[62]; NMMSN-zh: 人参根茎与根 (species origin: *Panax ginseng*)

✗ Ginseng Rhizome and Root

**Rule 7.6.2:** Standard taxonomic name rule. When naming NMMSN, prioritize using the current standard taxonomic name. The standard taxonomic name should primarily refer to the following databases: “Catalogue of Life”[63] and “Species 2000 China Node”[34].

**Ex.** ✓ *Tetradium ruticarpum* ✗ *Euodia rutaecarpa* ✗ *Evodia ruticarpa var. officinalis* ✗ *Evodia ruticarpa var. bodinieri*

In the *Chinese Pharmacopoeia: 2020 Edition: Volume I* (hereinafter referred to as “ChP”)[23], the NMM “吴茱萸” is recorded as a multi-species NMM, based on *Euodia rutaecarpa* or *Evodia ruticarpa var. officinalis* or *Evodia ruticarpa var. bodinieri*. However, all three of these scientific names are non-standard. The standardized name for all of them is *Tetradium ruticarpum*. Therefore, “吴茱萸” is still actually a single-species NMM. Its correct NMMSN is:

NMMSN: Tetradium ruticarpum Fruit[64]; NMMSN-zh: 吴茱萸果实

**Rule 7.6.3:** Multiple species origins naming rule. When the same part of multiple species is used as the medicinal part of an NMM, and these species are interchangeable (i.e., in an “or” relationship), in order to clarify the specific species origins of the NMM, all species origins should be fully listed in the NMMSN. Taxonomic names of species are listed side by side, connected by the term “vel” (Latin conjunction meaning “or”) (In NMMSN-zh, the word “或” is used), and arranged in alphabetical order by the Latin names. When the genus name appears repeatedly among the multiple species, the genus name can be omitted the second time it appears.

**Ex.** Multiple species originate from the same genus. In ChP, “麻黄” has 3 species origins: *Ephedra sinica* or *Ephedra intermedia* or *Ephedra equisetina*. Thus, it should be named as:

Ephedra equisetina vel Ephedra intermedia vel Ephedra sinica Stem-herbaceous

However, because they share the same genus name, it should be further abbreviated as:

NMMSN: Ephedra equisetina vel intermedia vel sinica Stem-herbaceous[27]; NMMSN-zh: 木贼麻黄或中麻黄或草麻黄草质茎

**Ex.** Multiple species originate from different genera. For example:

Cremastra appendiculata vel Pleione bulbocodioides vel Pleione yunnanensis Pseudobulb

After abbreviating the genus name for the last two species, it becomes:

NMMSN: Cremastra appendiculata vel Pleione bulbocodioides vel yunnanensis Pseudobulb[65]; NMMSN-zh: 杜鹃兰或独蒜兰或云南独蒜兰假鳞茎

**Rule 7.6.4:** Refinement of species origin rule. When a more refined species origin can be specified, we should prioritize using this more refined species origin for referring to the NMM.

**Ex.** For “麻黄” with a more refined species origin, we can adopt the following names:

NMMSN: Ephedra sinica Stem-herbaceous[30]; NMMSN-zh: 草麻黄草质茎

NMMSN: Ephedra intermedia Stem-herbaceous[66]; NMMSN-zh: 中麻黄草质茎

NMMSN: Ephedra equisetina Stem-herbaceous[67]; NMMSN-zh: 木贼麻黄草质茎

**Rule 7.6.5:** Species inclusion rule. For a multi-species NMM, if the taxonomic levels of its species origins are in a hierarchical relationship, only the species with the highest hierarchical level is used for naming.

**Ex.** ChP records the species origins of “山楂” as *Crataegus pinnatifida* and *Crataegus pinnatifida var. major*. However, since *Crataegus pinnatifida var. major* is a variety of *Crataegus pinnatifida*, the latter includes the former, so we should not include this variety as a species origin of “山楂” in the naming.

✓ NMMSN: Crataegus pinnatifida Fruit[68]; NMMSN-zh: 山楂果实

× Crataegus pinnatifida vel pinnatifida var major Fruit

The NMM with more refined species origin can be named separately to clarify:

✓ NMMSN: Crataegus pinnatifida var major Fruit[69]; NMMSN-zh: 山里红果实

**Rule 7.6.6:** Naming rule for NMMs with uncertain specific species information. For NMMs where the specific species information is uncertain, the naming can be done using only the genus name, followed by “unspecified” (in NMMGN-zh,

add “未定种”) to indicate that the species information has yet to be clarified.

**Ex.** There are thousands of species under the genus *Taraxacum*[28], all of which could potentially be used as an NMM:

NMMSN: Taraxacum unspecified Herb[29]; NMMSN-zh: 蒲公英属未定种全草

**Rule 7.6.7:** Rule for omitting non-legitimate characters in taxonomic names. If the taxonomic name of the species origin of the NMM contains non-legitimate characters, these should be omitted.

**Ex.** ✓ NMMSN: Ziziphus jujuba var spinosa Seed[70]; NMMSN-zh: 酸枣种子

✗ Ziziphus jujuba var. spinosa Seed

**Rule 7.7:** Medicinal part naming rules.

**Rule 7.7.1:** Syntax rule. The medicinal part in NMMSN should be named using singular nouns or noun phrases in English.

**Ex.** ✓ NMMSN: Panax ginseng Leaf[71]; NMMSN-zh: 人参叶

✗ Panax ginseng Leaves

**Rule 7.7.2:** Capitalization and hyphenation rule. For readability, the first letter of the medicinal part should be capitalized. If the medicinal part consists of multiple words, they should be connected with a hyphen “-”.

**Ex.** NMMSN: Solidago decurrens Herb[61]; NMMSN-zh: 一枝黄花全草

**Ex.** NMMSN: Ephedra sinica Stem-herbaceous[30]; NMMSN-zh: 草麻黄草质茎

**Rule 7.7.3:** Multiple medicinal parts naming rule. If an NMM uses multiple different medicinal parts from the same species, they can be connected with “and” (for NMMSN-zh, using “与”). If an NMM can use multiple interchangeable medicinal parts from the same species, they can be connected with “or” (for

NMMSN-zh, using “或”). The order of medicinal parts is based on the alphabetical order.

**Ex.** NMMSN: Vincetoxicum pycnostelma Rhizome and Root[72]; NMMSN-zh: 徐长卿根茎与根

**Rule 7.7.4:** Refinement of medicinal part rule. When a more specific medicinal part can be identified, we should prioritize using the refined medicinal part to refer to the NMM. This naming rule helps us to clarify the medicinal part of the NMM further.

**Ex.** “Vincetoxicum pycnostelma Rhizome and Root” can be further refined as:

NMMSN: Vincetoxicum pycnostelma Rhizome[73]; NMMSN-zh: 徐长卿根茎

NMMSN: Vincetoxicum pycnostelma Root[74]; NMMSN-zh: 徐长卿根

**Rule 7.8:** Special description naming rules.

**Rule 7.8.1:** Syntax rule. Some NMMs require specific characteristics or need to undergo special initial preparation at the production site before they can be used as medicine. For these NMMs, adjectives, adjective phrases, nouns, or appositives can be used for naming in element III.

**Rule 7.8.2:** Capitalization and hyphenation rule. For readability, the first letter of the special description should be capitalized. If the description consist of multiple words, they should be connected with a hyphen “-”.

**Ex.** NMMSN: Zingiber officinale Rhizome[75]; NMMSN-zh: 姜根茎

NMMSN: Zingiber officinale Rhizome Fresh[76]; NMMSN-zh: 鲜姜根茎

**Ex.** NMMSN: Curcuma wenyujin Rhizome[77]; NMMSN-zh: 温郁金根茎

NMMSN: Curcuma wenyujin Rhizome Freshly-sliced[78]; NMMSN-zh: 鲜切片温郁金根茎

**Rule 7.8.3:** Rule for genuine regional NMMs (道地药材). For some NMMs that specifically need to be sourced from genuine regions, geographical nouns can be used as special descriptions. For genuine regional Chinese NMMs, the standard English names of the provincial capitals of the People's Republic of China are generally used, while in NMMSN-zh, the abbreviation of the provincial capital plus "产" (meaning produced in) is used.

**Ex.** NMMSN: Fritillaria thunbergii Bulb Zhejiang[79]; NMMSN-zh: 浙产浙贝母鳞茎

**Rule 7.9:** Processing method naming rules.

**Rule 7.9.1:** Syntax rule. PNMM must include a processing method. PNMM is named based on its corresponding ANMM by adding an English adjective, adjective phrase, appositive, or abbreviation related to the processing method in name element IV. The corresponding Chinese word for the processing method should end with "制".

**Ex.** ANMM: NMMSN: Artemisia annual Part-aerial[80]; NMMSN-zh: 黄花蒿地上部

PNMM: NMMSN: Artemisia annual Part-aerial Segmented[81]; NMMSN-zh: 段制黄花蒿地上部

**Rule 7.9.2:** Capitalization and hyphenation rule. For readability, the first letter of the processing method should be capitalized. If the processing method consists of multiple words, they should be connected using a hyphen "-".

**Rule 7.9.3:** Naming rule for NMM with multiple processing methods. For PNMMs that require multiple processing methods, they should be connected using "and", with the term for the later processing method placed after (In NMMSN-zh, processing methods, because they end in "制", do not need logical connectors; the term for the later processing method is placed before). The sequence of processing methods is related to the processing order, so the order of the words cannot be changed arbitrarily.

**Ex.** ✓ NMMSN: Ephedra sinica Stem-herbaceous Segmented and Aquafried-honey[26]; NMMSN-zh: 蜜炙制段制草麻黄草质茎

× Ephedra sinica Stem-herbaceous Aquafried-honey and Segmented

**Rule 7.9.4:** Naming rule for processing methods of Chinese NMMs. The processing methods for Chinese NMMs are based on “0231 General Rules for Processing” in the *Chinese Pharmacopoeia: 2020 Edition: Volume IV*[82]. When naming the processing method, if a specific type of processing can be determined, it should be used; if not, the general category can be used.

**Ex.** NMMSN: Crataegus pinnatifida Fruit Cleaned and Stirfried-golden[83]; NMMSN-zh: 炒黄制净制山楂果实

NMMSN: Crataegus pinnatifida Fruit Cleaned and Stirfried-charred[84]; NMMSN-zh: 炒焦制净制山楂果实

**Rule 7.9.5:** Classification rule for natural medicinal processing (炮制) methods of Chinese NMMs. There are three main categories of processing methods for Chinese NMMs: processing by cleaning (净制), processing by cutting (切制), and processing by preparing (备制). Chinese NMMs that have undergone processing by cutting are assumed to have been processed by cleaning. Any that needs processing by preparing must first go through processing by cleaning or cutting.

**Ex.** ✓ NMMSN: Ephedra sinica Stem-herbaceous Segmented and Aquafried-honey[26]; NMMSN-zh: 蜜炙制段制草麻黄草质茎

× Ephedra sinica Stem-herbaceous Aquafried-honey

**Ex.** ✓ NMMSN: Zingiber officinale Rhizome Cleaned and Stirfried-sand[85]; NMMSN-zh: 砂炒制净制姜根茎

× Zingiber officinale Rhizome Stirfried-sand

**Rule 7.10:** Naming rule for NMMs of non-species origin or with unclear species origin. If the NMM to be named is non-species or its species origin is hard to determine, its NMMSN can be named using an English common name.

**Ex.** NMMSN: Talc[86]; NMMSN-zh: 滑石

NMMSN: Talc Pulverized[87]; NMMSN-zh: 粉制滑石

**Rule 8:** NMMGN Rules.

**Rule 8.1:** Conciseness rule. Owing to the detailed naming requirements of NMMSN, which include specifying the species origin, medicinal part, special description, and processing method, NMMSN tends to be lengthy. To facilitate everyday usage and clinical prescription, each NMM is also assigned a corresponding shorter NMMGN.

**Rule 8.2:** Conventional naming rule for NMMGN-zh of Chinese NMMs. The NMMGN-zh of a Chinese NMM prefers the commonly used name. If the NMM is from the ChP, the Chinese name listed in the ChP is generally the NMMGN-zh. For NMMs originated from a refined single species, to prevent naming conflicts, the prefix “单”/“独” (meaning “single”) is added to the beginning of some NMMGN-zh to differentiate them.

**Rule 8.3:** Pinyin naming priority rule for NMMGN of Chinese NMM. To correspond with NMMGN-zh, we prefer the pinyin name of the NMMGN-zh when naming the NMMGN. The pinyin does not use tone marks or spaces but uses hyphens to connect the pinyin of different characters, capitalizes the first letter, and uses “v” instead of “ü”.

**Ex.** NMMSN: Erycibe obtusifolia Stem[88]; NMMSN-zh: 丁公藤茎; NMMGN: Dan-ding-gong-teng; NMMGN-zh: 单丁公藤 (To differentiate with “丁工藤”, add prefix “单”)

NMMSN: Erycibe obtusifolia vel schmidtii Stem[89]; NMMSN-zh: 丁公藤或光叶丁公藤茎; NMMGN: Ding-gong-teng; NMMGN-zh: 丁公藤

**Ex.** NMMSN: Ligustrum lucidum Fruit[90]; NMMSN-zh: 女贞果实; NMMGN: Nv-zhen-zi; NMMGN-zh: 女贞子

**Rule 8.4:** Minimum length rule for NMMGN-zh. NMMGN-zh must be named using two or more characters.

**Ex.** NMMSN: Prunus mume Fruit[91]; NMMSN-zh: 梅果实

NMMGN-zh: ✓ 乌梅 × 梅

**Rule 8.5:** First-come-first-served rule. Due to the brevity of NMMGN, naming conflicts might arise. In such cases, we adhere to the priority of the first-come NMMGN, and subsequent NMMGNs must incorporate additional information for differentiation.

**Ex.** Suppose we have already named the following NMM:

NMMSN: Ephedra sinica Stem-herbaceous[30]; NMMSN-zh: 草麻黄草质茎; NMMGN: Cao-ma-huang; NMMGN-zh: 草麻黄

In the aforementioned case, the NMMGN-zh of the NMM did not mention its medicinal part information since it was prioritized, and thus omitted. But if we were to further name the root of *Ephedra sinica*, its NMMGN, and NMMGN-zh would need appropriate differentiation:

NMMSN: Ephedra sinica Root[31]; NMMSN-zh: 草麻黄根; NMMGN: Cao-ma-huang-gen; NMMGN-zh: 草麻黄根

**Rule 9:** NMM ID rules. Each NMM is assigned a unique NMM ID. The encoding rule for NMM ID is: NMM-XXXX , where XXXX is a 4-digit number in base 36 (i.e., 0-9, A-Z), starting from 0001 and increasing to ZZZZ, encoding up to $36^4 - 1 = 1,679,615$ kinds of NMMs. NMM ID is case-insensitive, but for ease of reading, it is usually written in uppercase.

**Ex.** NMM-ID: NMM-0001[80]

NMMSN: Artemisia annua Part-aerial

NMMSN-zh: 黄花蒿地上部

NMMGN: Qing-hao

NMMGN-zh: 青蒿

**Ex.** NMM-ID: NMM-0002[81]

NMMSN: Artemisia annua Part-aerial Segmented

NMMSN-zh: 段制黄花蒿地上部

NMMGN: Qing-hao-duan

NMMGN-zh: 青蒿段

**Rule 10:** Standard referencing rules.

**Rule 10.1:** First complete appearance rule. To ensure that the NMM name in a scientific text accurately corresponds to the actual NMM it refers to, it is recommended to present the "NMMSN (NMM ID, NMMGN)" format when an NMM first appears in an independent text (such as a paper or encyclopedia). In subsequent appearances of the NMM within the text, only NMMSN, NMMGN, or NMM ID may be used for reference.

**Ex.** Artemisia annua Part-aerial (NMM-0001, Qing-hao) is a commonly used Chinese natural medicinal material. Artemisia annua Part-aerial has the effect of treating malaria.

**Ex.** Artemisia annua Part-aerial (NMM-0001, Qing-hao) is a commonly used Chinese natural medicinal material. Qing-hao has the effect of treating malaria.

**Ex.** Artemisia annua Part-aerial (NMM-0001, Qing-hao) is a commonly used Chinese natural medicinal material. NMM-0001 has the effect of treating malaria.

**Ex.** 黄花蒿地上部（NMM-0001，青蒿）是一种常用的中药材。黄花蒿地上部可用于治疗疟疾。

**Ex.** 黄花蒿地上部（NMM-0001，青蒿）是一种常用的中药材。青蒿可用于治疗疟疾。

**Ex.** 黄花蒿地上部（NMM-0001，青蒿）是一种常用的中药材。NMM-0001 可用于治疗疟疾。

**Rule 10.2:** Appendix complete information rule. If a study involves a large number of NMMs, presenting all NMMSN, NMMGN, and NMM ID in the main text can make it excessively lengthy. In such cases, only one of the NMMSN, NMMGN, or NMM ID can be used to refer to the NMM in the main text; however, in the appendix, a complete list in the format "NMM ID – NMMSN – NMMGN" should be provided.

## Supplementary Text D: Systematic Nomenclature for Natural Medicinal Materials (SNNMM, 天然药材系统命名法, Chinese version)

**规则 1:** 两名一 ID 规则。每个天然药材（Natural Medicinal Material, NMM）均被被赋予一个天然药材系统名（NMM Systematic Name, NMMSN）、一个天然药材通用名（NMM Generic Name, NMMGN）和一个天然药材 ID（NMM ID）。

**规则 2:** 唯一规则。在 SNNMM 下产生的 NMMSN、NMMGN 和 NMM ID 均唯一。

**规则 3:** ANMM/PNMM 不同名规则。SNNMM 将 NMMs 分为 3 类，原始天然药材（Raw NMMs, RNMMs）、农产天然药材（Agricultural NMMs, ANMMs）和炮制天然药材（Processed NMMs, PNMMs）（**Fig. 2b**）。RNMMs 一般按照农产品进行管理，PNMMs 一般按照药品进行管理。

**规则 4:** 合法字符规则。为便于世界上任何一个国家的人们都能够通过他们国家的标准键盘简单的输入 NMMSN 或 NMMGN，NMMSN 和 NMMGN 的命名只允许使用以下字符：大小写拉丁字母 a-zA-Z、连词符“-”。

**规则 5:** 大小写不敏感规则。在 NMMSN 或 NMMGN 中，尽管会有大小写字母同时使用的情况，但这仅仅是为了便于 NMMSN 和 NMMGN 的阅读的便利。SNNMM 中，不得通过采用不同大小写的方式以区分不同的 NMM。

**规则 6:** 正体规则：NMMSN 或 NMMGN 采用正体书写。

**规则 7:** NMMSN 规则。

**规则 7.1:** NMMSN 标准中文对应名规则。为便于中文语境下的交流，每个 NMM 在命名 NMMSN 的同时，SNNMM 也会给出 NMMSN 的对应的标准中文对应名，即天然药材系统中文名（NMMSN-zh）。

**规则 7.2:** NMMSN 构词规则。NMMSN 由以下 4 种命名组件构成（**Fig. 2a**）：I. 物种基源（Species origin）、II. 药用部位（Medicinal part）、III. 特殊形容（Special description）、IV. 炮制方法（Processing method）。

**规则 7.3:** NMMSN 语法分析规则。根据自然语言处理和计算语言学原理，NMMSN 的语法分析（Parsing）结构如 **Supplementary Fig. S4** 所示。

**规则 7.4:** NMMSN 最小构词规则。ANMM 的 NMMSN 至少包含以下 2 种命名组件：I 和 II；PNMM 的 NMMSN 至少包含以下 3 种命名组件：I、II 和 IV（**Supplementary Fig. S4**）。

**规则 7.5:** NMMSN 语序规则。NMMSN 命名组件的语序不可改变，语序为：I-II-III-IV；对于 NMMSN-zh，命名组件为符合中文习惯，语序为：IV-III-I-II。

**规则 7.6:** 物种基源命名规则。

**规则 7.6.1:** 完整物种学名规则。命名时尽可能优先使用物种的完整的拉丁文物种学名。在可以明确具体物种时，不得仅采用物种的属名或种加词进行命名。

**Ex.** ✓ NMMSN: Solidago decurrens Herb[61]; NMMSN-zh: 一枝黄花全草（物种基源：*Solidago decurrens*）

✗ Solidago Herb

**Ex.** ✓ NMMSN: Panax ginseng Rhizome and Root[62]; NMMSN-zh: 人参根茎与根（物种基源：*Panax ginseng*）

✗ Ginseng Rhizome and Root

**规则 7.6.2:** 标准物种学名规则。NMMSN 命名时优先使用现行的标准物种学名。标准物种学名优先参考以下数据库：“Catalogue of Life”[63]、“物种 2000 中国节点”[34]。

**Ex.** ✓ *Tetradium ruticarpum* ✗ *Euodia rutaecarpa* ✗ *Evodia ruticarpa var. officinalis* ✗ *Evodia ruticarpa var. bodinieri*

在《中国药典 • 2020 年版 • 一部》（下简称“ChP”）[23] 中记载 NMM “吴茱萸”为多物种基源 NMM：基于 *Euodia rutaecarpa* 或 *Evodia ruticarpa var. officinalis* 或 *Evodia ruticarpa var. bodinieri*。然而，这三个物种学名均非标准物种学名，其正名均为 *Tetradium ruticarpum*。因此，“吴茱萸”实际仍然为单物种基源 NMM。其正确 NMMSN 为：

NMMSN: Tetradium ruticarpum Fruit[64]; NMMSN-zh: 吴茱萸果实

**规则 7.6.3:** 多物种基源命名规则。多种物种的相同部位用作一个 NMM 的药用部位，且这些物种互为可替代关系（即“或”关系）时，为了明确 NMM 的具体物种基源，NMMSN 命

名时要完整列出所有的物种基源。物种学名并列，中间采用“vel”（拉丁文连词，意为“或”）连接（NMMSN-zh 中使用“或”连接），排序时以拉丁字母为顺序。当多物种中出现重复的属名时，属名第二次出现时可省略。

**Ex.** 多物种基源来自相同属物种。ChP 中，“麻黄”有 3 种物种基源：*Ephedra sinica* 或 *Ephedra intermedia* 或 *Ephedra equisetina*。因此应当命名为：

Ephedra equisetina vel Ephedra intermedia vel Ephedra sinica Stem-herbaceous

但由于属名一致，须进一步缩写为：

NMMSN: Ephedra equisetina vel intermedia vel sinica Stem-herbaceous[27]; NMMSN-zh: 木贼麻黄或中麻黄或草麻黄草质茎

**Ex.** 多物种基源来自不同属物种。如：

Cremastra appendiculata vel Pleione bulbocodioides vel Pleione yunnanensis Pseudobulb

须进一步缩写后两种物种的属名：

NMMSN: Cremastra appendiculata vel Pleione bulbocodioides vel yunnanensis Pseudobulb[65]; NMMSN-zh: 杜鹃兰或独蒜兰或云南独蒜兰假鳞茎

**规则 7.6.4:** 细化物种基源规则。当能够明确更细化的物种基源时，我们应当优先使用更细化的物种基源对 NMM 进行指代。

**Ex.** 我们可以对具有更细化的物种基源的“麻黄”采取以下命名：

NMMSN: Ephedra sinica Stem-herbaceous[30]; NMMSN-zh: 草麻黄草质茎

NMMSN: Ephedra intermedia Stem-herbaceous[66]; NMMSN-zh: 中麻黄草质茎

NMMSN: Ephedra equisetina Stem-herbaceous[67]; NMMSN-zh: 木贼麻黄草质茎

**规则 7.6.5:** 物种包含规则。对于一个多物种基源 NMM，若其物种基源的物种分类等级存在包含关系，则仅使用具有最高包含等级的物种进行命名。

**Ex.** ChP 记载“山楂”的物种基源为 *Crataegus pinnatifida* 和 *Crataegus pinnatifida var. major*。但由于 *Crataegus pinnatifida var. major* 是 *Crataegus pinnatifida* 的变种，后者对前者是包含关系，因此我们在命名时不应该包含此变种作为“山楂”的物种基源。

✓ NMMSN: Crataegus pinnatifida Fruit[68]; NMMSN-zh: 山楂果实

✗ Crataegus pinnatifida vel pinnatifida var major Fruit

具有更细化物种基源的 NMM 可以单列，以明确：

✓ NMMSN: Crataegus pinnatifida var major Fruit[69]; NMMSN-zh: 山里红果实

**规则 7.6.6:** 具体物种信息不确定的 NMM 的命名规则。对于具体物种信息不确定的 NMM，可以仅用属名进行命名，并在属名后加“unspecified”（NMMGN-zh 中添加“未定种”）以提示物种信息尚未被明确。

**Ex.** 蒲公英属下有数千种物种 [28]，均可潜在用作 NMM：

NMMSN: Taraxacum unspecified Herb[29]; NMMSN-zh: 蒲公英属未定种全草

**规则 7.6.7:** 物种学名非合法字符省略规则。如果 NMM 的物种基源的物种学名含有非合法字符，则省略。

**Ex.** ✓ NMMSN: Ziziphus jujuba var spinosa Seed[70]; NMMSN-zh: 酸枣种子

✗ Ziziphus jujuba var. spinosa Seed

**规则 7.7:** 药用部位命名规则。

**规则 7.7.1:** 语法规则。NMMSN 药用部位使用英文单数名词或名词短语进行命名。

**Ex.** ✓ NMMSN: Panax ginseng Leaf[71]; NMMSN-zh: 人参叶

✖ Panax ginseng Leaves

**规则 7.7.2:** 首字母大写和连词符规则。为便于阅读，药用部位的首字母应当大写；药用部位由多个词组成时，中间需要使用连词符“-”连接。

**Ex.** NMMSN: Solidago decurrens Herb[61]; NMMSN-zh: 一枝黄花全草

**Ex.** NMMSN: Ephedra sinica Stem-herbaceous[30]; NMMSN-zh: 草麻黄草质茎

**规则 7.7.3:** 多药用部位命名规则。如果一个 NMM 同时使用同一物种的多种不同的药用部位入药，药用部位间可以使用“and”（“与”）连接。如果一个 NMM 可以使用同一物种的多种不同的药用部位入药，且互为可替代关系，则药用部位间可以使用“or”（“或”）连接。药用部位的排序根据拉丁字母顺序。

**Ex.** NMMSN: Vincetoxicum pycnostelma Rhizome and Root[72]; NMMSN-zh: 徐长卿根茎与根

**规则 7.7.4:** 细化药用部位规则。当能够明确更细化的药用部位时，我们应当优先使用更细化的药用部位对 NMM 进行指代。该命名规则有助于我们进一步明确 NMM 的药用部位。

**Ex.** “Vincetoxicum pycnostelma Rhizome and Root”可以进一步细化为：

NMMSN: Vincetoxicum pycnostelma Rhizome[73]; NMMSN-zh: 徐长卿根茎

NMMSN: Vincetoxicum pycnostelma Root[74]; NMMSN-zh: 徐长卿根

**规则 7.8:** 特殊形容命名规则。

**规则 7.8.1:** 语法规则。一些 NMM 须具有某种特有的性状特征或经过某些特殊的产地初加工后方可入药，对于这些 NMM，可以在命名组件 III 中构词部分使用英文形容词、形容词短语、名词或同位语进行命名。

**规则 7.8.2:** 首字母大写和连词符规则。为便于阅读，特殊形容的首字母应当大写；特殊形容由多个词组成时，中间需要使用连词符“-”连接。

**Ex.** NMMSN: Zingiber officinale Rhizome[75]; NMMSN-zh: 姜根茎

NMMSN: Zingiber officinale Rhizome Fresh[76]; NMMSN-zh: 鲜姜根茎

**Ex.** NMMSN: Curcuma wenyujin Rhizome[77]; NMMSN-zh: 温郁金根茎

NMMSN: Curcuma wenyujin Rhizome Freshly-sliced[78]; NMMSN-zh: 鲜切片温郁金根茎

**规则 7.8.3:** 道地药材规则。某些 NMM 需要特别明确道地产区时，可以使用地理名词作为特殊形容。中国产道地 NMM 的产地名通常使用中华人民共和国省会名称的标准英文名，NMMSN-zh 中使用“省会标准缩写+产”作为其中文对应词。

**Ex.** NMMSN: Fritillaria thunbergii Bulb Zhejiang[79]; NMMSN-zh: 浙产浙贝母鳞茎

**规则 7.9:** 炮制方法命名规则。

**规则 7.9.1:** 语法规则。PNMM 必须包含炮制方法。PNMM 基于其对应的 ANMM 进行命名，通过在命名组件 IV 额外添加炮制方法所对应的英文形容词、形容词短语、同位语或炮制方法的英文缩写词（词组）进行命名。炮制方法的中文对应词必须以“制”结尾。

**Ex.** ANMM: NMMSN: Artemisia annual Part-aerial[80]; NMMSN-zh: 黄花蒿地上部

PNMM: NMMSN: Artemisia annual Part-aerial Segmented[81]; NMMSN-zh: 段制黄花蒿地上部

**规则 7.9.2:** 首字母大写和连词符规则。为便于阅读，炮制方法的首字母应当大写；炮制方法由多个词组成时，中间需要使用连词符“-”连接。

**规则 7.9.3:** 多重炮制的 NMM 命名规则。如果是多重炮制的 PNMM，需要使用多个炮制方法，其炮制方法间使用“and”连接，居于更晚炮制过程的炮制方法的词序居后（NMMSN-zh 的炮制方法由于有“制”作为词尾，因而无须使用逻辑连词；居于更晚炮制过程的炮制方法的词序居前）。炮制方法和炮制顺序相关，因此炮制方法的词序不可随意改变。

**Ex.** ✓ NMMSN: Ephedra sinica Stem-herbaceous Segmented and Aquafried-honey[26]; NMMSN-zh: 蜜炙制段制草麻黄草质茎

✗ Ephedra sinica Stem-herbaceous Aquafried-honey and Segmented

**规则 7.9.4:** 中国天然药材（中药材）炮制方法命名规则。中药材的炮制方法以《中国药典 •2020 年版 •四部》“0231 炮制通则”[82] 为基础。炮制方法命名时，如果能明确炮制细类，则优先采用炮制细类进行命名；在不能明确炮制细类时，可以采用炮制大类进行命名。

**Ex.** NMMSN: Crataegus pinnatifida Fruit Cleaned and Stirfried-golden[83]; NMMSN-zh: 炒黄制净制山楂果实

NMMSN: Crataegus pinnatifida Fruit Cleaned and Stirfried-charred[84]; NMMSN-zh: 炒焦制净制山楂果实

**规则 7.9.5:** 中药材炮制分类规则。中药材炮制（natural medicinal processing）分 3 大类：净制（processing by cleaning）、切制（processing by cutting）、备制（processing by preparing）。经过切制的中药材默认已经经过净制。凡需要备制的，其必须首先经过净制或切制。

**Ex.** ✓ NMMSN: Ephedra sinica Stem-herbaceous Segmented and Aquafried-honey[26]; NMMSN-zh: 蜜炙制段制草麻黄草质茎

✗ Ephedra sinica Stem-herbaceous Aquafried-honey

**Ex.** ✓ NMMSN: Zingiber officinale Rhizome Cleaned and Stirfried-sand[85]; NMMSN-zh: 砂炒制净制姜根茎

✗ Zingiber officinale Rhizome Stirfried-sand

**规则 7.10:** 非物种类或物种基源难以确定的 NMM 的命名规则。若待命名 NMM 为非物种类或物种基源难以明确，其 NMMSN 可采用英语习称进行命名。

**Ex.** NMMSN: Talc[86]; NMMSN-zh: 滑石

NMMSN: Talc Pulverized[87]; NMMSN-zh: 粉制滑石

**规则 8:** NMMGN 规则。

**规则 8.1:** 简洁规则。NMMSN 由于命名时需要明确 NMM 的物种基源、药用部位、特殊描述、炮制方法等信息，因此 NMMSN 通常较长。为了便于日常使用和临床处方时的便利，每个 NMM 也均有一个对应的较简短的 NMMGN。

**规则 8.2:** 中药材 NMMGN-zh 的惯常规则。中药材的 NMMGN-zh 优先采用已经惯用的中药材名。如果中药材出自 ChP，ChP 收录的中药材的中文名通常即为 NMMGN-zh。对于细化物种基源后的单物种基源中药材，为了防止命名冲突，个别中药材的 NMMGN-zh 开头添加“单”/“独”以区分。

**规则 8.3:** 中药材 NMMGN 的拼音名优先规则。为了使得中药材的 NMMGN 和 NMMGN-zh 呼应，我们在命名中药材的 NMMGN 时，优先使用中药材 NMMGN-zh 的拼音名。拼音名不使用声调和空格，使用连词符“-”连接不同汉字的拼音，首字母大写，并使用“v”代替“ü”。

**Ex.** NMMSN: Erycibe obtusifolia Stem[88]; NMMSN-zh: 丁公藤茎; NMMGN: Dan-ding-gong-teng; NMMGN-zh: 单丁公藤（为和“丁工藤”区分，添加“单”字。）

NMMSN: Erycibe obtusifolia vel schmidtii Stem[89]; NMMSN-zh: 丁公藤或光叶丁公藤茎; NMMGN: Ding-gong-teng; NMMGN-zh: 丁公藤

**Ex.** NMMSN: Ligustrum lucidum Fruit[90]; NMMSN-zh: 女贞果实; NMMGN: Nv-zhen-zi; NMMGN-zh: 女贞子

**规则 8.4:** 最短 NMMGN-zh 规则。NMMGN-zh 必须使用 2 个及以上的汉字进行命名。

**Ex.** NMMSN: Prunus mume Fruit[91]; NMMSN-zh: 梅果实

NMMGN-zh: ✓ 乌梅 × 梅

**规则 8.5:** 先到先得规则。由于 NMMGN 较短，在其命名时，难免遇到命名冲突的情况。在这种情况下，我们需要遵循先到的 NMMGN 优先，对于后到的 NMMGN，其须加一些额外信息以区分。

**Ex.** 假设我们已经对以下 NMM 进行命名：

NMMSN: Ephedra sinica Stem-herbaceous[30]; NMMSN-zh: 草麻黄草质茎; NMMGN: Cao-ma-huang; NMMGN-zh: 草麻黄

上述案例中该 NMM 的 NMMGN-zh 并未提及其药用部位信息，因为其是优先命名的，所以省略。但假设我们要进一步将 *Ephedra sinica* 的根用药，其 NMMGN 和 NMMGN-zh 就要适当做出区分：

NMMSN: Ephedra sinica Root[31]; NMMSN-zh: 草麻黄根; NMMGN: Cao-ma-huang-gen; NMMGN-zh: 草麻黄根

**规则 9:** NMM ID 规则。每种 NMM 被赋予唯一的 NMM ID。NMM ID 的编码规则为：NMM-XXXX，其中 XXXX 为 4 位 36 进制的数字（即 0-9，A-Z），从 0001 开始递增，止于 ZZZZ，至多可编码 $36^4 - 1 = 1{,}679{,}615$ 种 NMM。NMM ID 大小写不敏感，但在书写时为了方便阅读通常使用全大写。

**Ex.** NMM-ID: NMM-0001[80]

NMMSN: Artemisia annua Part-aerial

NMMSN-zh: 黄花蒿地上部

NMMGN: Qing-hao

NMMGN-zh: 青蒿

**Ex.** NMM-ID: NMM-0002[81]

NMMSN: Artemisia annua Part-aerial Segmented

NMMSN-zh: 段制黄花蒿地上部

NMMGN: Qing-hao-duan

NMMGN-zh: 青蒿段

**规则 10:** 标准指代规则。

**规则 10.1:** 首次完整出现规则。为了保证科学文本中的 NMM 名称和其实际指代的 NMM 准确对应，推荐在每个独立文本（如一篇论文、百科等）中首次出现某 NMM 时，以“NMMSN (NMM ID, NMMGN)”格式给出 NMM 的两名一 ID。在后续文本中 NMM 第二次出现时，允许只使用 NMMSN、NMMGN 或 NMM ID 进行指代。

**Ex.** Artemisia annua Part-aerial (NMM-0001, Qing-hao) is a commonly used Chinese natural medicinal material. Artemisia annua Part-aerial has the effect of treating malaria.

**Ex.** Artemisia annua Part-aerial (NMM-0001, Qing-hao) is a commonly used Chinese natural medicinal material. Qing-hao has the effect of treating malaria.

**Ex.** Artemisia annua Part-aerial (NMM-0001, Qing-hao) is a commonly used Chinese natural medicinal material. NMM-0001 has the effect of treating malaria.

**Ex.** 黄花蒿地上部（NMM-0001，青蒿）是一种常用的中药材。黄花蒿地上部可用于治疗疟疾。

**Ex.** 黄花蒿地上部（NMM-0001，青蒿）是一种常用的中药材。青蒿可用于治疗疟疾。

**Ex.** 黄花蒿地上部（NMM-0001，青蒿）是一种常用的中药材。NMM-0001 可用于治疗疟疾。

**规则 10.2:** 附录完整信息规则。如果某研究中涉及大量 NMM，在正文文本中一一给出所有的 NMMSN、NMMGN 和 NMM ID 可能导致正文文本过长。这种情况下，可以在正文中仅使用 NMMSN 或 NMMGN 或 NMM ID 中的一种来指代 NMM；但在附录中，需要给出“NMM ID – NMMSN – NMMGN”的完整列表。

## Supplementary Method E: SNNMM Algorithm (SNNMMA)

The algorithm and code of SNNMMA has been open-sourced and is available on GitHub (https://github.com/shennong-program/shennongname). The relevant Python package has been published and released on PyPI (https://pypi.org/project/shennongname).

This supplementary method primarily elucidates the fundamental principles of the SNNMMA, emphasizing its data input and output aspects. For detailed code implementation, readers are referred to the aforementioned repository.

The input to SNNMMA is a JSON Object. To elucidate, we illustrate with a common natural medicinal material, Mi-ma-huang (蜜麻黄) (**Supplementary Code S1**).

```
{
    "nmm_type": "processed",
    "species_origins": [["Ephedra sinica", "草麻黄"], "or", ["Ephedra intermedia", "中麻黄"], "or",
["Ephedra equisetina", "木贼麻黄"]],
    "medicinal_parts": [["stem herbaceous", "草质茎"]],
    "special_descriptions": [],
    "processing_methods": [["segmented", "段制"], "and", ["aquafried honey", "蜜炙制"]]
}
```

**Supplementary Code S1: Example of data structure for SNNMMA input.**

Within the aforementioned input data structure, users of SNNMMA are required to provide information pertaining to the NMM type along with the associated details for the four kinds of name elements. It is noteworthy that these four types of name elements are collectively stored utilizing a data structure denominated as NmmsnNeData.

The basic structure is as follows, exemplified by species_origins (**Supplementary Code S2**):

```
[
    ["Ephedra sinica", "草麻黄"],
    "or",
    ["Ephedra intermedia", "中麻黄"],
    "or",
```

```
    ["Ephedra equisetina", "木贼麻黄"]
]
```


**Supplementary Code S2: Example of data structure for NmmsnNeData in SNNMMA.**

The list encompassed in NmmsnNeData permits the incorporation of multiple name element pairs, each with a data substructure: ["name element in English or Latin", "name element in Chinese"], which can be interconnected by the logical operator strings "or" or "and".

Subsequently, when NmmsnNeData is conveyed to SNNMMA, the algorithm autonomously executes a series of processes for each name element type of NmmsnNeData. This includes string data verification, deduplication, sorting, character transformation, and more. Ultimately, the algorithm calculates and derives the NMM Systematic Name (NMMSN) and NMM Systematic Chinese Name (NMMSN-zh), presenting the results as another JSON Object (**Supplementary Code S3**).


```
{
    "success": true,
    "error_msg": "Pipe: construct_nmmsn_spe_ori. Status: warning. Reason: Multiple species origins detected.",
    "error_msg_en_zh": {
        "en": "Multiple species origins detected.",
        "zh": "检测到多个物种基源。"
    },
    "nmmsn": {
        "nmmsn": "Ephedra equisetina vel intermedia vel sinica Stem-herbaceous Segmented and Aquafried-honey",
        "nmmsn_zh": {
            "zh": "蜜炙制段制木贼麻黄或中麻黄或草麻黄草质茎",
            "pinyin": "mì zhì zhì duàn zhì mù zéi má huáng huò zhōng má huáng huò cǎo má huáng cǎo zhì jīng"
        },
        "nmmsn_name_element": {
            "nmm_type": "processed",
            "species_origins": [["Ephedra equisetina", "木贼麻黄"], "or", ["Ephedra intermedia", "中麻黄"], "or", ["Ephedra sinica", "草麻黄"]],
            "medicinal_parts": [["stem herbaceous", "草质茎"]],
            "special_descriptions": [],
            "processing_methods": [["segmented", "段制"], "and", ["aquafried honey", "蜜炙制"]]
```

```
            },
            "nmmsn_seq": [["Ephedra equisetina vel intermedia vel sinica", "木贼麻黄或中麻黄或草麻黄
"], ["Stem-herbaceous", "草质茎"], ["", ""], ["Segmented and Aquafried-honey", "蜜炙制段制"]]
        }
}
```

**Supplementary Code S3: Example of data structure for SNNMMA output (NMMSN construction successful)**

The output of the SNNMMA displays the following characteristics:

Once user data is successfully processed by SNNMMA to construct an NMMSN, the value of success will be set to true. Moreover, the resultant information post-NMMSN construction by SNNMMA will be stored under the nmmsn key.

The specific meanings of each hierarchical key in the SNNMMA output are described as follows:

- error_msg: In the SNNMM framework, there exist certain valid yet non-preferred rules. For instance, using multiple species origins for systematic naming of NMM is not recommended. The SNNMMA can automatically detect such anomalies during NMMSN construction. Consequently, there might be instances where the NMMSN is successfully constructed, yet the error_msg remains populated, recording any issues encountered during the process. These error messages adhere to a standardized format: Pipe: xxx. Status: xxx. Reason: xxx. and are stored within error_msg.

- error_msg_en_zh: To enhance the user experience for both English and Chinese users, error messages in SNNMMA have been localized. Information pertaining to the Reason in error_msg is processed and stored in both English and Chinese within error_msg_en_zh.en and error_msg_en_zh.zh respectively. This ensures that even users with programming or language barriers can clearly understand any issues encountered during NMMSN construction by SNNMMA.

- nmmsn: This key houses all information directly related to NMMSN.

- nmmsn.nmmsn: Represents the successfully constructed NMM Systematic Name.

- nmmsn.nmmsn_zh: nmmsn.nmmsn_zh.zh denotes the successfully constructed NMM Systematic Chinese Name, while nmmsn.nmmsn_zh.pinyin represents the corresponding pinyin.

- nmmsn.nmmsn_name_element: The data structure of this key mirrors the structure of input data (**Supplementary Code S1**). However, the order of elements within the NmmsnNeData data structure might be adjusted or reordered based on the SNNMM rules. For example, the order in species_origins might change from [["Ephedra sinica", "草麻黄"], "or", ["Ephedra intermedia", "中麻黄"], "or", ["Ephedra equisetina", "木贼麻黄"]] to [["Ephedra equisetina", "木贼麻黄"], "or", ["Ephedra intermedia", "中麻黄"], "or", ["Ephedra sinica", "草麻黄"]] due to the alphabetical ordering being e -> i -> s among the three species origins.

- nmmsn.nmmsn_seq: Given that NMMSN comprises four name elements, this key stores the NMMSN corresponding to each name element. This sequenced NMMSN, within ShennongName, can be utilized to distinctively display each name element in a unique color, enhancing user-friendliness.

If SNNMMA encounters issues during the NMMSN construction process and fails, the output from SNNMMA will be strikingly similar to that shown in **Supplementary Code S3**. However, the value of success will be set to false, and the resulting JSON Object will not include the nmmsn key and its corresponding value (**Supplementary Code S4**).

```
{
    "success": false,
    "error_msg": "...",
    "error_msg_en_zh": {
        "en": "...",
        "zh": "..."
    }
}
```

**Supplementary Code S4: Example of data structure for SNNMMA output (NMMSN construction failed).**

## Supplementary Method F: Multilingual Markdown (MLMD)

The source code for the MLMD parser has been open-sourced and can be accessed on GitHub: https://github.com/shennong-program/mlmd.

The associated TypeScript package has been released on npm: https://www.npmjs.com/package/mlmd.

This Supplementary Method primarily introduces the core design principles, essential syntax, and usage methods of MLMD. Please refer to the repository above for the detailed syntax and parsing code of MLMD.

### F.1 Core design principles

Multilingual Markdown (MLMD) is a newly designed lightweight markup language explicitly tailored for managing multilingual text. The syntax of MLMD integrates the strengths of Markdown[92] while being specially designed for multilingual parallel corpora.

The primary objective of MLMD is to allow users to write and manage multilingual parallel text in an easy-to-read and easy-to-write plain text format.

Thanks to the MLMD syntax that can manage multilingual content in a single document, it can be widely applied in the following scenarios:

- Multilingual text writing, content creation
- Unified/Structured management, storage of multilingual texts
- Multilingual display of multilingual texts
- Cross-language text annotation, entity annotation
- Cross-language translation, proofreading
- Cross-language machine translation
- Text analysis/mining of multilingual texts
- Natural language processing of multilingual texts

- ...

## F.2 Writing, storage, and file extension

The content of MLMD is in plain text, making it possible to compose using any plain text editor.

When saving MLMD content as an independent file, the .mlmd extension should be used. For instance, if we save MLMD text in a file named abc.mlmd, then abc represents the filename and .mlmd is the standard extension for MLMD.

Since MLMD text is plain text, an independent MLMD text can technically be embedded/stored as a single string within other file formats or databases such as JSON, SQL, MongoDB, etc.

For example, consider the following MLMD text (**Supplementary Code S5**):

```
{{langs|zh|en}}

你好，世界！
Hello, world!
```

**Supplementary Code S5: MLMD example: Hello, World!**

It can be stored as a single string in a JSON structure (**Supplementary Code S6**):

```
{
    "mlmd_str": "{{langs|zh|en}}\n\n 你好，世界！ \nHello, world!"
}
```

**Supplementary Code S6: MLMD stored as a string inside a JSON structure.**

## F.3 Essential syntax

### F.3.1 MLMD language header

Since MLMD can be used to store parallel corpora of any number of languages, it is required to explicitly define the languages of the text stored in MLMD in the first line of every MLMD file, using the format `{{langs|<language_code_1>|<language_code_2>|...}}`. Following the syntax of MLMD, MLMD documents can manage parallel corpora of any number of languages. Thus, non-repeating language codes can be used in the MLMD language header.

It is noteworthy that the first language code in the MLMD language header corresponds to the primary language of that particular MLMD document.

For instance, the language header of a bilingual MLMD document in Chinese and English is as follows (**Supplementary Code S7**):

```
{{langs|zh|en}}
```

**Supplementary Code S7: MLMD language header example: Chinese-English.**

Whereas the language header of a trilingual MLMD document in Chinese, English, and Latin is as follows (**Code S8**):

```
{{langs|zh|en|la}}
```

**Supplementary Code S8: MLMD language header example: Chinese-English-Latin.**

### F.3.2 Multilingual parallel paragraphs

In MLMD, multilingual parallel corpora are managed at the paragraph level. Each multilingual paragraph is treated as a Block within MLMD. Within each Block, the paragraphs of text in different languages are arranged in the order of language codes and are separated by a line break (when stored as strings, the newline character `\n` is used). Different Blocks are separated by a blank line (when stored as strings, two newline characters `\n\n` are used). For examples (**Supplementary Codes S9-S10**):

```
{{langs|zh|en}}
```

```
这是第 1 段中文。
This is the 1st paragraph in English.

这是第 2 段中文。
This is the 2nd paragraph in English.
```

**Supplementary Code S9: MLMD multilingual parallel paragraphs example: Chinese-English.**

```
{{langs|zh|en|la}}

这是第 1 段中文。
This is the 1st paragraph in English.
Hic est paragraphus Latinus primus.

这是第 2 段中文。
This is the 2nd paragraph in English.
Hic est paragraphus Latinus secundus.
```

**Supplementary Code S10: MLMD multilingual parallel paragraphs example: Chinese-English-Latin.**

Incidentally, the concept of a Block is one of the cores of MLMD, as when we wish to store data of different structures (such as links, images, etc.) within MLMD, they are essentially treated as individual Blocks. Blocks' management approach of Blocks allows MLMD to be conveniently parsed. The Blocks corresponding to multilingual parallel paragraphs are abbreviated as "Multi".

### F.3.3 Language-invariant paragraphs

In multilingual texts, certain text paragraphs remain the same across all languages. We refer to such text as language-invariant paragraphs. When a Block in our saved MLMD document contains only one paragraph of text, it is automatically regarded as a language-invariant text. This type of Block is abbreviated as "Mono". For example (**Supplementary Code S11**):

```
{{langs|zh|en}}

```

```
这是第 1 个 Block (Multi)中的中文段落。
This is an English paragraph in the 1st Block (Multi).

This is a language-invariant paragraph in English in the 2nd Block (Mono).

这是第 3 个 Block (Mono)中的一段中文的跨语言不变段落。

这是第 4 个 Block (Multi)中的中文段落。
This is an English paragraph in the 4th Block (Multi).
```

**Supplementary Code S11: MLMD language-invariant paragraphs example.**

### F.3.4 Emphasis

In MLMD, text can be emphasized by wrapping with ** for bold emphasis and * for italic emphasis. For example (**Supplementary Code S12**):

```
{{langs|zh|en}}

第 1 个 Block (Multi)中的中文**加粗**段落。
A **bold** paragraph in English within the 1st Block (Multi).

A **bold** and *italic* language-invariant paragraph in English in the 2nd Block (Mono).

第 3 个 Block (Mono)中的一段中文的**加粗**和*斜体*的跨语言不变段落。

第 4 个 Block (Multi)中的中文*斜体*段落。
An *italic* paragraph in English within the 4th Block (Multi).
```

**Supplementary Code S12: MLMD emphasis example.**

### F.3.5 Headings

MLMD supports up to six levels of headings. Headings are denoted using the # symbol. By prefixing a paragraph with 1-6 of the # symbols, you can designate the heading levels 1-6, respectively. To separate multilingual headings, use the | symbol. If there is no |, the heading content is treated as Mono. For example (**Supplementary Code S13**):

```
{{langs|zh|en}}

# 一级标题 | Heading Level-1

## 二级标题 | Heading Level-2

### 三级标题 | Heading Level-3

#### 四级标题 | Heading Level-4

##### 五级标题 | Heading Level-5

###### 六级标题 | Heading Level-6

# 一级标题

## Heading Level-2
```

**Supplementary Code S13: MLMD headings example.**

### F.3.6 Coreference annotation

The central linguistic principle of this syntax is "coreference consistency". It recognizes that different terms or phrases can refer to the same inherent concept or entity in multilingual or monolingual texts. This capacity to discern that, despite variations in form, the essence of the reference remains unchanged underpins this principle. In MLMD, terms with this shared referential quality are described as having "coreference consistency".

Consider this illustrative example (**Supplementary Code S14**):

```
{{langs|zh|en}}

神农被中国人认为是医药学的始祖。
Shennong is considered the progenitor of medicine and pharmacy by the Chinese.

炎帝被中国人认为是医药学的始祖。
Yan Emperor is considered the progenitor of medicine and pharmacy by the Chinese.
```

**Supplementary Code S14: MLMD coreference consistency example (not annotated).**

To those familiar with the historical background, irrespective of the terms “神农”, “炎帝”, “Shennong”, or “Yan Emperor” being used, they understand these allude to the same historical figure, specifically “神农” (Shennong). However, these could appear as four separate concepts for those without this knowledge. To convey this textual coreference consistency within the content, MLMD introduces a dedicated coreference annotation syntax. This facilitates easier textual annotations of coreferential relationships and enhances machine translation and AI natural language processing capabilities in understanding coreference consistency. The specific syntax is as follows:

The double bracket notation `[[...]]` is utilized for coreference annotation. No spaces must exist between `[[` and `]]`.

The primary coreference annotations are of two types:

1. When the term’s expression aligns with its reference: `[[term's reference]]`
2. When there’s a divergence between the term’s expression and its reference: `[[term's reference|term's expression]]`

By employing this approach, the sentences mentioned above can be annotated as (**Supplementary Code S15**):

```
{{langs|zh|en}}

[[神农]]被中国人认为是医药学的始祖。
[[神农|Shennong]] is considered the progenitor of medicine and pharmacy by the Chinese.

[[神农|炎帝]]被中国人认为是医药学的始祖。
[[神农|Yan Emperor]] is considered the progenitor of medicine and pharmacy by the Chinese.
```

**Supplementary Code S15: MLMD coreference consistency example (annotated).**

In this framework, “神农” stands as the Primary Term denoting the concept, while “炎帝”, “Shennong”, and “Yan Emperor” are its coreferential terms. Hence, with the above coreference annotations, even those without the specific historical context can infer that these sentences all point to the same person, specifically “神农”.

### F.3.7 Entity annotation

In natural language texts, certain terms often represent specific entities. We can achieve a unified annotation by utilizing the coreference annotation syntax to corefer a term to its corresponding entity. In practice, terms can be coreferred to their corresponding entity ID.

For instance, both “青蒿” and “Qing-hao” correspond to the NMM ID “NMM-0001”. Hence, we can annotate the entity as follows (**Supplementary Code S16**):

```
{{langs|zh|en}}

[[NMM-0001|青蒿]]是一种天然药材。
[[NMM-0001|Qing-hao]] is a kind of Natural Medicinal Material.
```

**Supplementary Code S16: MLMD entity annotation example.**

### F.3.8 Comments

MLMD uses <!-- ... --> for comments. For example (**Supplementary Code S17**):

```
{{langs|zh|en}}

第 1 个 Block (Multi)中的中文**加粗**段落。 <!-- 单行注释 -->
A **bold** paragraph in English within the 1st Block (Multi). <!-- Single-line comment -->

A **bold** and *italic* language-invariant paragraph in English in the 2nd Block (Mono). <!-- Single-line comment -->

<!-- 多行注释
Multi-line comment
-->

第 3 个 Block (Mono)中的一段中文的**加粗**和*斜体*的跨语言不变段落。 <!-- 单行注释 -->

第 4 个 Block (Multi)中的中文*斜体*段落。
An *italic* paragraph in English within the 4th Block (Multi).
```

**Supplementary Code S17: MLMD comment example.**

#### F.3.9 Templates

For texts with specific functionalities, MLMD employs the template syntax {{...}} for annotation. No spaces are allowed between the double curly brackets {{ and }}. For instance, the MLMD language header is a type of specialized template.

MLMD can be extended to cater to various unique template functional requirements. Detailed template syntax can be found in the MLMD repository. Here, we emphasize the citation template directly related to this paper.

#### F.3.10 Citations

Users can conveniently add citations to the MLMD text using the citation template: {{ref|@<ref_id>}}. This makes MLMD suitable for rigorous academic texts. For detailed information related to the ref_id, one can store it in BibTeX format using {{ref-citation|bibtex|<ref_citation_info>}}. The ref_id must match with the ref_id in the BibTeX within ref_citation_info. For example (**Supplementary Code S18**):

```
{{langs|zh|en}}

"AI 介导的五阶段科学革命"，这一理论在《AI 成为主脑科学家》这篇论文中被首次提出
{{ref|@ai_masterbrain}}。
The theory of the "Five Stages of AI-involved Scientific Revolution" was first introduced in the paper
titled "AI Becomes a Masterbrain Scientist" {{ref|@ai_masterbrain}}.

{{ref-citation|bibtex|
@article{ai_masterbrain,
  title={AI becomes a masterbrain scientist},
  author={YANG, Zijie and WANG, Yukai and ZHANG, Lijing},
  journal={bioRxiv},
  pages={2023--04},
  year={2023},
  publisher={Cold Spring Harbor Laboratory}
}
}}
```

**Supplementary Code S18: MLMD citation example.**

On the ShennongAlpha website, all references are stored in the ShennongKB reference collection. Each reference is assigned a globally unique ID in the format sna-ref-xxx. Hence, in ShennongAlpha, one can employ a special reference annotation like {{ref|[[sna-ref-1]]}}. Here, [[sna-ref-1]] serves as the @<ref_id>. Since sna-ref-1 is encapsulated within [[]], it signifies that [[sna-ref-1]] inherently is a coreference related to the citation. Therefore, ShennongAlpha parses MLMD automatically fetches the reference information about sna-ref-1 from ShennongKB. In this context, there's no further need to supplement the specific reference details through {{ref-citation|bibtex}}.

### F.4 Machine Translation Compatibility

MLMD natively supports a novel machine translation approach introduced in this paper: Neural Machine Translation based on Coreference Primary Term (NMT-CPT).

Using coreference annotation to denote the relationships between standard Primary Terms and their translations, NMT-CPT offers users a streamlined and interactive presentation of translation results through the ShennongTranslate user interface. This approach significantly enhances the interpretability of machine translations.

### F.5 HTML Parsing

Through a parsing flow: MLMD → Abstract Syntax Tree (AST)[93] → HTML, MLMD can be rendered into HTML with rich text formatting, enhancing the user experience while reading its content, ShennongAlpha natively supports HTML rendering of MLMD, offering the bilingual content presentation in four modes: "Chinese-English", "English-Chinese", "Chinese", and "English" (**Supplementary Fig. S9**).

## Supplementary Method G: NMM knowledge curation

To manage standardized knowledge of NMMs in ShennongAlpha, we implemented a comprehensive knowledge curation process comprising the following steps:

**Step 1: Collection of NMM text information.**

We gathered authoritative textual information on NMMs from authoritative sources, especially the 2020 and 2015 editions of the *Chinese Pharmacopoeia*[23,32] and the Chinese Medicinal Information Platform[33]. This provided a robust foundation for subsequent data processing.

**Step 2: Extraction and standardization of key NMM naming components.**

We employed a combination of programming and manual review methods to extract and standardize information related to the four components of the NMM Systematic Name: species origin, medicinal part, special description, and processing method.

**Species origin:**

Due to historical naming conventions, a single plant/species may have multiple scientific names. Without standardizing species names, the Systematic Names derived from these names may result in synonyms for the same entity. Therefore, it is essential to ensure that the species names used for the Systematic Names are standardized.

To ensure we always use standardized species names, we adopted the following approach:

1. **Automated verification:** We developed a Python-based, automated program (which is open-sourced; the relevant code is available on GitHub: https://github.com/shennong-program/shennongname) to verify each NMM-related species name against the authoritative species database "SP2000 China Node"[34].

2. **Manual review:** If the species name recorded in the literature differs from that in SP2000 China Node (i.e., if the species name is a synonym), we conducted a further manual review to determine whether the retrieved standardized species name was indeed the correct species related to the NMM, thereby ensuring the accuracy of the information.

3. **Data integration:** When incorporating the relevant NMM information into the ShennongKB, we included both the original recorded species name (to allow users to check the original information) and the standardized species name. We always

used the standardized species names to name a Systematic Name in the Systematic Nomenclature (**Supplementary Text C, Rule 7.6.2**).

For example, the NMM “Wu-zhu-yu” (吴茱萸) in the *Chinese Pharmacopoeia: 2020 Edition: Volume I*[23] is recorded as a multi-species-origin NMM (derived from *Euodia rutaecarpa*, *Evodia ruticarpa var. officinalis*, or *Evodia ruticarpa var. bodinieri*). However, these names are synonyms, and the correct standardized name is *Tetradium ruticarpum*. Therefore, “Wu-zhu-yu” is actually a single-species-origin NMM, and its correct Systematic Name is “Tetradium ruticarpum Fruit”[64].

To verify the species names of the NMM “Wu-zhu-yu,” we used the following automated program. For example, inputting “Evodia ruticarpa var. bodinieri” (**Supplementary Code S19**):

```
from shennongname.utils.sp2000_china import standardize_species_scientific_name

output = standardize_species_scientific_name(
    species_scientific_name="Evodia ruticarpa var. bodinieri",
    sp2000_china_api_key="<your_sp2000_china_api_key>",
)
```

**Supplementary Code S19: Using the automated species name standardization program to query and standardize “Evodia ruticarpa var. bodinieri” (which is a non-standard species name).**

The program automatically matches the species name in the SP2000 China Node database and returns the matched species name ("Evodia ruticarpa var. bodinieri") and its corresponding Name Code ("T20171000067168"; the Name Code is the ID corresponding to this species in SP2000 China Node). It also checks whether the matched species name is a standard name. If it is not standard ("name_status": "synonym"), the program finds the standardized species name ("Tetradium ruticarpum") and its Name Code ("T20171000067157"). The results are returned in the Python dictionary output (**Supplementary Code S20**):

```
{
    "matched_a_species": True,
    "matched_species_scientific_name": "Evodia ruticarpa var. bodinieri",
    "matched_species_name_code": "T20171000067168",
    "name_status": "synonym",
```

```
    "standardized_species_scientific_name": "Tetradium ruticarpum",
    "standardized_species_name_code": "T20171000067157",
}
```


**Supplementary Code S20: Species name standardization result from Supplementary Code S19.**

If querying an already standardized name like “Tetradium ruticarpum” (**Supplementary Code S21**):

```
from shennongname.utils.sp2000_china import standardize_species_scientific_name

output = standardize_species_scientific_name(
    species_scientific_name="Tetradium ruticarpum",
    sp2000_china_api_key="<your_sp2000_china_api_key>",
)
```

**Supplementary Code S21: Using the automated species name standardization program to query and standardize “Tetradium ruticarpum” (which is a standardized species name).**

The program returns (**Supplementary Code S22**):


```
{
    "matched_a_species": True,
    "matched_species_scientific_name": " Tetradium ruticarpum",
    "matched_species_name_code": "T20171000067157",
    "name_status": "accepted name",
    "standardized_species_scientific_name": "Tetradium ruticarpum",
    "standardized_species_name_code": "T20171000067157",
}
```


**Supplementary Code S22: Species name standardization result from Supplementary Code S21.**

Note that "name_status" as "accepted name" indicates that it is the current standardized species name.

Based on the results of the automated program, we further confirmed through manual review that "Tetradium ruticarpum" is the standardized species name corresponding to "Evodia ruticarpa var. bodinieri". Therefore, when naming "Wu-zhu-yu," we used "Tetradium ruticarpum" instead of "Evodia ruticarpa var. bodinieri" as its species origin. Through these efforts, we ensure that the species information is always authoritative and accurate.

**Medicinal part, special description, and processing method:**

Since these components lacked prior standardization, we manually annotated them and constructed standardized term lists. Users can access these lists via hyperlinks on the ShennongName interface (**Fig. 3** ②). Processing methods primarily reference the natural medicinal processing methods documented in the *Chinese Pharmacopoeia: 2020 Edition: Volume IV*[82].

**Step 3: Automated NMM naming using ShennongName.**

With standardized information for each NMM's four name components, we employed the automated SNNMM Algorithm (**Supplementary Method E**) within the ShennongName Python package to generate the NMM Systematic Name and NMM Systematic Chinese Name.

We then assigned the NMM Generic Name. Each NMM's Generic Chinese Name utilizes its conventional Chinese name (**Supplementary Text C, Rule 8.2**), which is transliterated into Pinyin for the NMM Generic Name (**Supplementary Text C, Rule 8.3**). For example, "青蒿" becomes "Qing-hao". To resolve naming conflicts due to homophones in Pinyin, we adjusted some Generic Names to ensure global uniqueness (**Supplementary Text C, Rule 8.5**).

Finally, we encoded each NMM to generate a unique NMM ID. This structured nomenclature data was updated in ShennongKB and displayed in the "Systematic Nomenclature for Natural Medicinal Materials" section on the ShennongAlpha knowledge page (**Fig. 4** ⑦**; Supplementary Fig. S7**).

**Step 4: Annotation of hierarchical relationships between NMMs.**

To construct a more interconnected knowledge base, we annotated hierarchical parent-child relationships between NMMs:

**1. Processed NMMs derived from Agricultural NMMs:**

Processed NMMs are often obtained from Agricultural NMMs through specific natural medicinal processing methods (**Supplementary Text C, Rule 7.9.1**). For example, "Artemisia annua Part-aerial Segmented"[81] is produced by segmenting "Artemisia

annua Part-aerial”[80]. Therefore, “Artemisia annua Part-aerial” is the parent NMM of “Artemisia annua Part-aerial Segmented” .

**2. Specific NMMs derived from ambiguous NMMs:**

Some NMMs with ambiguous name components (such as species origins) can be further specified. For instance, “Ephedra equisetina vel intermedia vel sinica Stem-herbaceous (NMM-0006, Ma-huang)”[27] has a species origin that could be *Ephedra sinica*, *Ephedra intermedia*, or *Ephedra equisetina*. This NMM can be further specified as:

- Ephedra sinica Stem-herbaceous (NMM-0003, Cao-ma-huang)[30]
- Ephedra intermedia Stem-herbaceous (NMM-0004, Zhong-ma-huang)[66]
- Ephedra equisetina Stem-herbaceous (NMM-0005, Mu-zei-ma-huang)[67]

Here, NMM-0006 serves as the parent NMM for NMM-0003, NMM-0004, and NMM-0005.

These relationships were imported into ShennongKB and are displayed in the “Systematic Nomenclature for Natural Medicinal Materials” section on ShennongAlpha (**Supplementary Fig. S7 ⑬, ⑭**). Users can view parent and child NMMs along with their NMM IDs. When users hover over an NMM ID (**Supplementary Fig. S7 ⑮**), a tooltip appears, providing a summary of the knowledge related to that NMM (**Supplementary Fig. S7 ⑮’**). Users can also click on the NMM ID or the tooltip to navigate directly to the NMM’s knowledge page. This interactive feature enables users to quickly explore and understand related NMMs on the ShennongAlpha platform.

**Step 5: Translation into English using ShennongTranslate and MLMD formatting.**

We utilized ShennongTranslate to automatically translate collected NMM textual information (primarily in Chinese) into English. The original and translated texts were organized into MLMD format (**Supplementary Method F**) and uploaded to ShennongKB. This enables the platform to display bilingual text based on user language preferences (**Fig. 4 ⑨**), such as zh-en, en-zh, zh, or en (**Supplementary Fig. S9**).

**Step 6: Collection of NMM-associated information.**

To enrich the Knowledge Base of each NMM with multidimensional data, we collected information on their ingredients, related targets, and diseases from external traditional Chinese medicine databases, including HERB[35] and HIT[36].

Subsequently, we standardized the collected data by cross-referencing each ingredient, related target, and disease with their corresponding authoritative external databases (such as PubMed, NCBI, and MeSH). This standardization enabled us to present the information in a user-friendly manner on the ShennongAlpha NMM knowledge pages. Specifically:

- **Ingredients:** We consulted the authoritative compound database PubChem[94] to obtain standardized PubChem IDs, PubChem Names, CAS Registry Numbers, and Canonical SMILES for each ingredient. We designed an "Ingredients" section on the ShennongAlpha NMM knowledge pages to display this information (**Supplementary Fig. S8 ①**). The PubChem IDs and CAS Registry Numbers are hyperlinked to their respective PubChem and CAS database entries (**Supplementary Fig. S8 ⑦, ⑧**), allowing users to quickly access detailed information about each compound.

- **Related targets:** We referenced the authoritative gene database NCBI[95] to retrieve the NCBI Gene ID, Gene Official Symbol, and Gene Official Full Name for each target. This information is presented in the "Related Targets" section of the ShennongAlpha knowledge pages (**Supplementary Fig. S8 ⑨**). Users can click on the hyperlinked Gene IDs (**Supplementary Fig. S8 ⑬**) to navigate directly to the corresponding NCBI gene pages, facilitating rapid access to detailed gene target information.

- **Related diseases:** We standardized each disease using the authoritative medical terminology database MeSH[96] to obtain the MeSH ID and MeSH Heading. The information on diseases associated with each NMM is displayed in the "Related Diseases" section of the ShennongAlpha knowledge pages (**Supplementary Fig. S8 ⑭**). Users can click on the hyperlinked MeSH IDs (**Supplementary Fig. S8 ⑰**) to be directed to the corresponding MeSH term pages, aiding in a quick understanding of the specific definitions and details about the diseases. For both related targets and diseases, we also provide the PubMed PMIDs, which are linked to the PubMed database[97] (**Supplementary Fig. S8 ⑲**), to help users further understand and verify the literature evidence supporting these relationships.

Additionally, we have provided download buttons on the ShennongAlpha knowledge pages for the Ingredients, Related Targets, and Related Diseases sections. Users can download the related structured data as CSV files (**Supplementary Fig. S8 ⑳**), enabling convenient further analysis of the information.

**Step 7: Manual review of key information.**

We conducted manual reviews of key information on NMM knowledge pages. Reviewed sections display the reviewer's name (**Supplementary Fig. S7 ⑰**), enhancing credibility assessment and acknowledging contributions.

In summary, through this systematic approach combining automated processing and manual curation, we effectively managed standardized NMM knowledge within ShennongAlpha.

# Supplementary Method H: Coreference-based Graph Search (CGS)

## H.1 Foundational CGS

To elucidate the process of CGS, we illustrate it with a straightforward case:

Consider the set of nodes:

$$A, B, C, D, E, F$$

The coreference relationships are given as follows:

$$A \rightarrow B$$
$$B \rightarrow \boldsymbol{C}$$
$$D \rightarrow B$$
$$E \rightarrow \boldsymbol{F}$$

Here, $\boldsymbol{C}, \boldsymbol{F}$ are manually designated as Primary Terms.

The Coreference Primary Term Graph (CPTG) can be constructed from the above nodes and directed edges. This CPTG must satisfy the following conditions:

1. It should be a Directed Acyclic Graph (DAG).
2. The out-degree of any Primary Term in the graph should be zero.
3. The out-degree of any node should be less than or equal to one, indicating that no branching nodes are present.

The graph constructed from the given example is illustrated below:

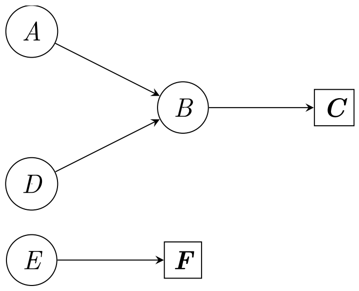


Once a CPTG is constructed, preliminary graph computations can be conducted to optimize queries to identify the ultimate Primary Term corresponding to any term node.

The results of these computations are stored in a newly created dictionary. Notably, within this dictionary, relationships of directed edges from a Primary Term to itself (e.g., $\boldsymbol{C} \rightarrow \boldsymbol{C}$) are also included. This feature allows the queried term to be recognized as a Primary Term.

$$A \rightarrow \boldsymbol{C}$$
$$B \rightarrow \boldsymbol{C}$$
$$D \rightarrow \boldsymbol{C}$$
$$E \rightarrow \boldsymbol{F}$$
$$\boldsymbol{C} \rightarrow \boldsymbol{C}$$
$$\boldsymbol{F} \rightarrow \boldsymbol{F}$$

Thus, by searching this dictionary, it can be swiftly determined whether a corresponding Primary Term exists for any given term, including Primary Terms, by verifying its presence in the dictionary. If found, the value corresponding to this term will be its associated Primary Term.

The pseudocode for the Foundational CGS Algorithm is as follows:

**Algorithm 1** Foundational CGS Algorithm

```
Input: Set of nodes N, Coreference relationships R
Output: Dictionary D storing the ultimate Primary Terms
Initialize the dictionary D
Construct the Coreference Primary Term Graph (CPTG) G from the given N
  and R
for each node n in G do
   if n is not a Primary Term then
      Find the ultimate Primary Term p for n
      if p exists then
         Add the mapping n → p to D
      end if
   else
      Add the self-referencing mapping n → n to D
   end if
end for
For searching:
Given any term t, query D to ascertain whether a corresponding Primary Term
  exists
if t exists in D then
   Retrieve the corresponding Primary Term p from D
end if
```

### H.2 Weighted CGS

During the construction of the CPTG, directed edges are typically extracted automatically from databases. Consequently, we often cannot satisfy the third requirement of CPTG (i.e., no branching nodes). We can assign a weight to each directed edge based on the Foundational CGS and conduct a weighted graph search in such instances.

Let's illustrate this with a simple example:

Consider the set of nodes:

$$A, B, C, D, E$$

The coreference relationships are given as follows:

$$A \xrightarrow{w_{AB}=1} B$$

$$B \xrightarrow{w_{BC}=2} \boldsymbol{C}$$

$$D \xrightarrow{w_{DB}=1} B$$

$$B \xrightarrow{w_{BE}=1} \boldsymbol{E}$$

Here, $\boldsymbol{C}$ and $\boldsymbol{E}$ are manually designated as Primary Terms. Each coreference relationship is assigned a weight to signify the importance of that relationship.

A CPTG can be constructed from the above nodes and directed edges. This CPTG meets the first two requirements of a CPTG in Foundational CGS but does not satisfy the third requirement.

The graph constructed from the given example would be as follows:

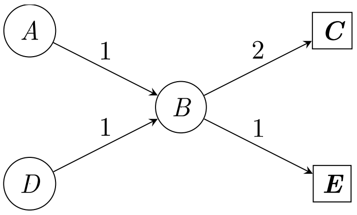


In this CPTG, we observe an anomalous branching node $B$, which points to both $\boldsymbol{C}$ and $\boldsymbol{E}$ simultaneously. Therefore, under these circumstances, when conducting preliminary graph computations to search for the ultimate Primary Term of all nodes, we proceed as follows: For each given node, we acquire all its downstream directed edges. By evaluating the weights of these edges, we select the downstream node corresponding to the edge with the highest weight as the next node. We repeat this process until any Primary Term is reached.

For instance, when searching for the ultimate Primary Term corresponding to node $A$, we navigate from $A$ to its sole downstream node $B$. However, $B$ actually has two downstream nodes, $\boldsymbol{C}$ and $\boldsymbol{E}$. At this juncture, since the weight of $B \to \boldsymbol{C}(2)$ is greater than that of $B \to \boldsymbol{E}(1)$, we further select $\boldsymbol{C}$ as $B$'s downstream node. As $\boldsymbol{C}$ is already a Primary Term, we conclude our graph search, determining that the ultimate Primary Term for $A$ is $\boldsymbol{C}$, with the graph search path being $A \to B \to \boldsymbol{C}$, rather than $A \to B \to \boldsymbol{E}$.

Through this method, we can still acquire a dictionary consistent with the Foundational CGS, as illustrated below:

$$A \to \boldsymbol{C}$$
$$B \to \boldsymbol{C}$$
$$D \to \boldsymbol{C}$$
$$\boldsymbol{C} \to \boldsymbol{C}$$
$$\boldsymbol{E} \to \boldsymbol{E}$$

The pseudocode for the Weighted CGS Algorithm is as follows:

**Algorithm 2** Weighted CGS Algorithm

```
Input: Set of nodes N, Coreference relationships R, Weights W of directed edges
Output: Dictionary D storing the ultimate Primary Terms
Initialize the dictionary D
Construct the Coreference Primary Term Graph (CPTG) G from the given N, R, and W
for each node n in G do
   if n is not a Primary Term then
      Find the ultimate Primary Term p for n using weighted graph search
      if p exists then
         Add the mapping n → p to D
      end if
   else
      Add the self-referencing mapping n → n to D
   end if
end for
For searching:
Given any term t, query D to ascertain whether a corresponding Primary Term exists
if t exists in D then
   Retrieve the corresponding Primary Term p from D
end if
```

### H.3 Primary Term Extractor

When we have a dictionary *D* that maps terms to their primary terms, efficiently identifying all potential terms from *D* within any given string *S* becomes a significant challenge. This is especially true when *D* contains a large number of terms with high similarity. For example, consider the terms “Ma-huang” (NMM ID: NMM-0006)[27] and “Ma-huang-duan” (NMM ID: NMM-000A)[98], both of which are Generic Names of NMMs recorded in ShennongKB and included in our Primary Term Dictionary.

Suppose a user provides the following two strings:

```
String 1: What is the NMM ID of Ma-huang?
String 2: What is the NMM ID of Ma-huang-duan?
```

If we use a simple string matching approach, for String 2, we might match both “Ma-huang” and “Ma-huang-duan” from *D*. This overlap can interfere with subsequent knowledge extraction, leading to ambiguity. Therefore, we need a specialized algorithm to accurately extract terms without such conflicts.

To address this issue, we introduced the Primary Term Extractor algorithm within our CGS framework. This algorithm enables us to efficiently and accurately extract all potential terms from *D* that are present in any given string *S*. Specifically, the Primary Term Extractor employs a Trie data structure[99] and a longest-match strategy. The algorithm is detailed as follows:

**Algorithm 3** Primary Term Extractor

**Input:** Dictionary $D$ mapping terms to their primary terms. Text string $S$
**Output:** Mapping $M$ from terms found in $S$ to their primary terms
Build a Trie $T$ from the keys of $D$
Initialize an empty mapping $M$
Let $n \leftarrow$ length of $S$
Let index $i \leftarrow 0$
**while** $i < n$ **do**
  $Candidates \leftarrow$ all prefixes of $S[i:]$ that are in Trie $T$
  **if** $Candidates$ is not empty **then**
    $longest\_match \leftarrow$ the longest string in $Candidates$
    $primary\_term \leftarrow D[longest\_match]$
    Add mapping $longest\_match \rightarrow primary\_term$ to $M$
    $i \leftarrow i + \text{length}(longest\_match)$
  **else**
    $i \leftarrow i + 1$
  **end If**
**end while**

By constructing a Primary Term Dictionary of NMM-related terms using CGS and employing the Primary Term Extractor algorithm, we can efficiently extract potential NMM terms from any text and rapidly identify their corresponding primary terms (i.e., NMM IDs). We tested this approach on a dataset containing 40,000 texts, which included both Chinese and English texts with various NMM terms in both languages, and found that it achieved a 100% detection rate. This performance significantly surpasses that of commonly used methods based on Jieba tokenization[46]. In the latter approach, NMM-related terms were added to the Jieba tokenizer, and potential NMM terms were identified by traversing the tokenized words; however, this method achieved a detection rate of only 75% (**Supplementary Fig. S14**).

The CGS algorithms described above have been implemented in Python and are available as open-source software. Users can access them via GitHub (https://github.com/shennong-program/pycgs) or PyPI (https://pypi.org/project/pycgs). The datasets and code for the comparative tests between CGS and Jieba in extracting NMM terms from texts are also available on GitHub (https://github.com/shennong-program/pycgs).

# I Supplementary References


23 Chinese Pharmacopoeia Commission. *Pharmacopoeia of the People's Republic of China: 2020 Edition: Volume I.* (China Medical Science Press, 2020).

26 ShennongAlpha. *ShennongAlpha Knowledge: NMM-000B, Ephedra sinica Stem-herbaceous Segmented and Aquafried-honey*, <https://shennongalpha.westlake.edu.cn/en-zh/knowledge/nmm-000b> (2024).

27 ShennongAlpha. *ShennongAlpha Knowledge: NMM-0006, Ephedra equisetina vel intermedia vel sinica Stem-herbaceous*, <https://shennongalpha.westlake.edu.cn/en-zh/knowledge/nmm-0006> (2024).

28 Catalogue of Life. *Taraxacum*, <https://www.catalogueoflife.org/data/taxon/7SSF> (2024).

29 ShennongAlpha. *ShennongAlpha Knowledge: NMM-01YF, Taraxacum unspecified Herb*, <https://shennongalpha.westlake.edu.cn/en-zh/knowledge/nmm-01yf> (2024).

30 ShennongAlpha. *ShennongAlpha Knowledge: NMM-0003, Ephedra sinica Stem-herbaceous*, <https://shennongalpha.westlake.edu.cn/en-zh/knowledge/nmm-0003> (2024).

31 ShennongAlpha. *ShennongAlpha Knowledge: NMM-000G, Ephedra sinica Root*, <https://shennongalpha.westlake.edu.cn/en-zh/knowledge/nmm-000g> (2024).

32 Chinese Pharmacopoeia Commission. *Pharmacopoeia of the People's Republic of China: 2015 Edition: Volume I.* (China Medical Science Press, 2015).

33 China Medical Information Platform. *China Medical Information Platform*, <https://www.dayi.org.cn/> (2024).

34 Species 2000 China Node. *Species 2000 China Node*, <http://www.sp2000.org.cn/> (2024).

35 Fang, S. *et al.* HERB: a high-throughput experiment-and reference-guided database of traditional Chinese medicine. *Nucleic Acids Research* **49**, D1197-D1206 (2021).

36 Yan, D. *et al.* HIT 2.0: an enhanced platform for Herbal Ingredients' Targets. *Nucleic acids research* **50**, D1238-D1243 (2022).

46 Jieba Developers. *Jieba*, <https://github.com/fxsjy/jieba> (2024).

61 ShennongAlpha. *ShennongAlpha Knowledge: NMM-0020, Solidago decurrens Herb*, <https://shennongalpha.westlake.edu.cn/en-zh/knowledge/nmm-0020> (2024).

62 ShennongAlpha. *ShennongAlpha Knowledge: NMM-001L, Panax ginseng Rhizome and Root*, <https://shennongalpha.westlake.edu.cn/en-zh/knowledge/nmm-001l> (2024).

63 Catalogue of Life. *Catalogue of Life*, <https://www.catalogueoflife.org/> (2024).

64 ShennongAlpha. *ShennongAlpha Knowledge: NMM-012W, Tetradium ruticarpum Fruit*, <https://shennongalpha.westlake.edu.cn/en-

zh/knowledge/nmm-012w> (2024).

65 ShennongAlpha. *ShennongAlpha Knowledge: NMM-00AC, Cremastra appendiculata vel Pleione bulbocodioides vel yunnanensis Pseudobulb*, <https://shennongalpha.westlake.edu.cn/en-zh/knowledge/nmm-00ac> (2024).

66 ShennongAlpha. *ShennongAlpha Knowledge: NMM-0004, Ephedra intermedia Stem-herbaceous*, <https://shennongalpha.westlake.edu.cn/en-zh/knowledge/nmm-0004> (2024).

67 ShennongAlpha. *ShennongAlpha Knowledge: NMM-0005, Ephedra equisetina Stem-herbaceous*, <https://shennongalpha.westlake.edu.cn/en-zh/knowledge/nmm-0005> (2024).

68 ShennongAlpha. *ShennongAlpha Knowledge: NMM-009X, Crataegus pinnatifida Fruit*, <https://shennongalpha.westlake.edu.cn/en-zh/knowledge/nmm-009x> (2024).

69 ShennongAlpha. *ShennongAlpha Knowledge: NMM-009Y, Crataegus pinnatifida var major Fruit*, <https://shennongalpha.westlake.edu.cn/en-zh/knowledge/nmm-009y> (2024).

70 ShennongAlpha. *ShennongAlpha Knowledge: NMM-005X, Ziziphus jujuba var spinosa Seed*, <https://shennongalpha.westlake.edu.cn/en-zh/knowledge/nmm-005x> (2024).

71 ShennongAlpha. *ShennongAlpha Knowledge: NMM-001S, Panax ginseng Leaf*, <https://shennongalpha.westlake.edu.cn/en-zh/knowledge/nmm-001s> (2024).

72 ShennongAlpha. *ShennongAlpha Knowledge: NMM-01MZ, Vincetoxicum pycnostelma Rhizome and Root*, <https://shennongalpha.westlake.edu.cn/en-zh/knowledge/nmm-01mz> (2024).

73 ShennongAlpha. *ShennongAlpha Knowledge: NMM-01MX, Vincetoxicum pycnostelma Rhizome*, <https://shennongalpha.westlake.edu.cn/en-zh/knowledge/nmm-01mx> (2024).

74 ShennongAlpha. *ShennongAlpha Knowledge: NMM-01MY, Vincetoxicum pycnostelma Root*, <https://shennongalpha.westlake.edu.cn/en-zh/knowledge/nmm-01my> (2024).

75 ShennongAlpha. *ShennongAlpha Knowledge: NMM-003G, Zingiber officinale Rhizome*, <https://shennongalpha.westlake.edu.cn/en-zh/knowledge/nmm-003g> (2024).

76 ShennongAlpha. *ShennongAlpha Knowledge: NMM-003M, Zingiber officinale Rhizome Fresh*, <https://shennongalpha.westlake.edu.cn/en-zh/knowledge/nmm-003m> (2024).

77 ShennongAlpha. *ShennongAlpha Knowledge: NMM-000T, Curcuma wenyujin Rhizome*, <https://shennongalpha.westlake.edu.cn/en-zh/knowledge/nmm-000t> (2024).

78 ShennongAlpha. *ShennongAlpha Knowledge: NMM-0015, Curcuma wenyujin Rhizome Freshly-sliced*, <https://shennongalpha.westlake.edu.cn/en-zh/knowledge/nmm-0015> (2024).

79 ShennongAlpha. *ShennongAlpha Knowledge: NMM-024C, Fritillaria thunbergii Bulb Zhejiang*, <https://shennongalpha.westlake.edu.cn/en-

zh/knowledge/nmm-024c> (2024).

80 ShennongAlpha. *ShennongAlpha Knowledge: NMM-0001, Artemisia annua Part-aerial*, <https://shennongalpha.westlake.edu.cn/en-zh/knowledge/nmm-0001> (2024).

81 ShennongAlpha. *ShennongAlpha Knowledge: NMM-0002, Artemisia annua Part-aerial Segmented*, <https://shennongalpha.westlake.edu.cn/en-zh/knowledge/nmm-0002> (2024).

82 Chinese Pharmacopoeia Commission. *Pharmacopoeia of the People's Republic of China: 2020 Edition: Volume IV*. (China Medical Science Press, 2020).

83 ShennongAlpha. *ShennongAlpha Knowledge: NMM-00A1, Crataegus pinnatifida Fruit Cleaned and Stirfried-golden*, <https://shennongalpha.westlake.edu.cn/en-zh/knowledge/nmm-00a1> (2024).

84 ShennongAlpha. *ShennongAlpha Knowledge: NMM-00A3, Crataegus pinnatifida Fruit Cleaned and Stirfried-charred*, <https://shennongalpha.westlake.edu.cn/en-zh/knowledge/nmm-00a3> (2024).

85 ShennongAlpha. *ShennongAlpha Knowledge: NMM-003L, Zingiber officinale Rhizome Cleaned and Stirfried-sand*, <https://shennongalpha.westlake.edu.cn/en-zh/knowledge/nmm-003l> (2024).

86 ShennongAlpha. *ShennongAlpha Knowledge: NMM-01XX, Talc*, <https://shennongalpha.westlake.edu.cn/en-zh/knowledge/nmm-01xx> (2024).

87 ShennongAlpha. *ShennongAlpha Knowledge: NMM-01XZ, Talc Pulverized*, <https://shennongalpha.westlake.edu.cn/en-zh/knowledge/nmm-01xz> (2024).

88 ShennongAlpha. *ShennongAlpha Knowledge: NMM-0022, Erycibe obtusifolia Stem*, <https://shennongalpha.westlake.edu.cn/en-zh/knowledge/nmm-0022> (2024).

89 ShennongAlpha. *ShennongAlpha Knowledge: NMM-0024, Erycibe obtusifolia vel schmidtii Stem*, <https://shennongalpha.westlake.edu.cn/en-zh/knowledge/nmm-0024> (2024).

90 ShennongAlpha. *ShennongAlpha Knowledge: NMM-00D7, Ligustrum lucidum Fruit*, <https://shennongalpha.westlake.edu.cn/en-zh/knowledge/nmm-00d7> (2024).

91 ShennongAlpha. *ShennongAlpha Knowledge: NMM-008W, Prunus mume Fruit*, <https://shennongalpha.westlake.edu.cn/en-zh/knowledge/nmm-008w> (2024).

92 CommonMark. *CommonMark*, <https://commonmark.org/> (2024).

93 mdast Developers. *mdast: Markdown Abstract Syntax Tree*, <https://github.com/syntax-tree/mdast> (2024).

94 National Library of Medicine. *PubChem*, <https://pubchem.ncbi.nlm.nih.gov/> (2024).

95 National Library of Medicine. *NCBI Gene*, <https://www.ncbi.nlm.nih.gov/gene> (2024).

96 National Library of Medicine. *MeSH*, <https://meshb.nlm.nih.gov/> (2024).

97 National Library of Medicine. *PubMed*, <https://pubmed.ncbi.nlm.nih.gov/> (2024).

98 ShennongAlpha. *ShennongAlpha Knowledge: NMM-000A, Ephedra equisetina*

*vel intermedia vel sinica Stem-herbaceous Segmented*, <https://shennongalpha.westlake.edu.cn/en-zh/knowledge/nmm-000a> (2024).

99 Wikipedia contributors. *Trie — Wikipedia, The Free Encyclopedia*, <https://en.wikipedia.org/w/index.php?title=Trie&oldid=1250000773> (2024).
